# Neuroprotective effects of *Platycladus orientalis* ethyl acetate fraction on retinal Müller cells: modulation of AKT1/mTOR and Raf-1/MEK1/2 pathways via AMPK activation

**DOI:** 10.3389/fmolb.2026.1802106

**Published:** 2026-05-22

**Authors:** Jing Xian Lim, Phaik Har Yong, Siew Huah Lim, Zhi Xiang Ng

**Affiliations:** 1 School of Biological and Environmental Sciences, Faculty of Science and Engineering, University of Nottingham Malaysia, Selangor, Malaysia; 2 School of Bioscience, Faculty of Pharmacy and Biomedical Sciences, MAHSA University, Selangor, Malaysia; 3 Department of Chemistry, Faculty of Science, University of Malaya, Kuala Lumpur, Malaysia

**Keywords:** apoptosis, diabetic retinopathy, medicinal plant, neurodegeneration, phytochemical, toxicity

## Abstract

**Background:**

*P. orientalis* has previously demonstrated protective effects against diabetic microvascular complications but its specific role in mitigating neurodegenerative events in diabetic retinopathy remains unknown. This study investigates the neuroprotective effects *of Platycladus orientalis* ethyl acetate fraction (EAPO) on retinal Müller cells (rMc-1) under different glucose stress.

**Methods:**

Bioactive compounds in the EAPO were identified using liquid chromatography-mass spectrometry, while its cytotoxicity was assessed *in vitro*. The expression of gene and protein biomarkers related to neuronal survival like mammalian target of rapamycin (mTOR), mitogen-activated extracellular signal-regulated kinase 1/2 (MEK1/2), rapid accelerated fibrosarcoma-1 (Raf-1), serine/threonine kinase 1 (AKT1), vascular endothelial growth factor (VEGF), and VEGF receptor 2 (VEGFR2) in EAPO- treated rMc-1 were compared with control group under different glucose concentrations.

**Results:**

Phytochemical analysis of EAPO revealed the presence of diterpenoids, monoterpene esters, phenolic glycosides, and saturated fatty acids. EAPO demonstrated low cytotoxicity (IC_50_ = 0.32 mg/mL) in rMc-1 under high-glucose stress conditions. Mechanistic study showed that EAPO treatment significantly mitigated glucose-induced cytotoxicity and neurodegeneration by downregulating the expression of VEGF, VEGFR2, mTOR, Raf-1, and MEK1/2, while enhancing phosphorylation of AKT1. Co-treatment with AICAR, an activator of AMP- activated protein kinase (AMPK), further amplified the neuroprotective effects of EAPO. In contrast, inhibition of AMPK using compound C exacerbated glucose-induced cytotoxicity and neurodegenerative signaling. The combination of EAPO and AICAR synergistically inhibited VEGF/VEGFR2 signaling and its downstream pathways (mTOR and Raf- 1/MEK1/2) while promoting AKT1-mediated neuronal survival.

**Conclusion:**

This study provides new mechanistic insight into the neuroprotective effects of *P. orientalis* on rMc-1 under diabetic stress. EAPO could attenuate high-glucose-induced neuronal toxicity in rMc-1 by modulating the AKT1/mTOR and Raf-1/MEK1/2 pathways through AMPK activation.

## 1 Introduction

Diabetic retinopathy (DR) is a common microvascular complication of diabetes and a leading cause of blindness among working-aged adults (Ng et al., 2012). It is increasingly recognized as a neurovascular disorder closely associated with neuronal toxicity (Simó et al., 2022). Retinal neurodegeneration occurs early in DR and is characterized by impaired neuronal function, glutamate-induced neurotoxicity, and diabetes-driven apoptosis, often accompanied by glutamate accumulation and reactive gliosis (Pillar et al., 2020; Viganò et al., 2025). Retinal Müller cells (rMc-1), the principal macroglia of the retina, play a critical role in maintaining retinal homeostasis by regulating glutamate metabolism and supporting neuronal function (Luo et al., 2021; Yang et al., 2024). Under hyperglycemic conditions, rMc-1 dysfunction induces gliosis, leading to glutamate excitotoxicity and activation of vascular endothelial growth factor (VEGF) signaling, which further contributes to neuronal damage and disease progression (Quincozes-Santos et al., 2021).

Both neurotoxicity and glial activation are regulated by the downstream mammalian target of rapamycin-serine/threonine kinase 1 (mTOR/AKT1) and rapid accelerated fibrosarcoma-1/mitogen-activated extracellular signal-regulated kinase 1/2 (Raf-1/MEK1/2) signaling pathways. Activation of the mTOR/AKT1 axis is linked to both enhanced neuronal survival and increased release of pro-inflammatory cytokines (Fakhri et al., 2021). Conversely, dysregulation of the Raf-1/MEK1/2 cascade contributes to pathological neuronal apoptosis and abnormal glial activation in DR (Chun et al., 2022). Adenosine monophosphate-activated protein kinase (AMPK), a central metabolic sensor, plays a pivotal role in mitigating DR by regulating downstream targets involved in mitochondrial function and energy homeostasis (Song et al., 2021). Within the retina, AMPK activation enhances autophagic degradation and suppresses neurotoxicity, thereby maintaining cellular integrity and conferring neuroprotection (Zhao et al., 2020). AMPK activation has been shown to attenuate neurodegeneration in DR, particularly through the preservation of Müller cell function (Shukal et al., 2022).

Although substantial progress has been made in the clinical management of DR, existing therapeutic modalities, including laser photocoagulation, vitrectomy, and intravitreal corticosteroid administration remain constrained by significant limitations (Ng et al., 2013). Conventional interventions often carry adverse effects (Wang et al., 2024), driving interest in alternative therapies, particularly medicinal plants, as potential treatments for the multifactorial pathogenesis of DR (Yong et al., 2025). *Platycladus orientalis* (L.) Franco, an evergreen conifer under the Cupressaceae family, has been used in traditional Chinese medicine for over 2,000 years (Shan et al., 2018). According to the classical text Shen Nong Ben Cao Jing, *Platycladus orientalis* is regarded as a top-grade medicinal herb traditionally used to alleviate paralysis, reduce palpitations, and promote longevity (Darwish et al., 2024). Recent pharmacological studies indicate that *P. orientalis* exerts protective effects against aging, diabetes, and neurodegenerative disorders (Sun et al., 2021; Yan et al., 2020). Specifically, leaf extracts exhibit neuroprotective effects by attenuating glutamate-induced neurotoxicity and suppressing mitochondrial-mediated neuronal apoptosis (Li X. et al., 2023). Collectively, these findings highlight the therapeutic potential of *P. orientalis* as a natural candidate for DR management.

Lim et al. (2025) recently reported that *P. orientalis* inhibits angiogenesis in human retinal endothelial cells via the Raf-1/MEK1/2 and AKT1/mTOR pathways. Building on this, the present study aims to investigate the role of AMPK as a central regulatory pathway and explores its interaction with VEGF-associated signaling, including mTOR/AKT1 and Raf-1/MEK1/2, in rMc-1. Unlike the previous focus on angiogenesis, this work provides new mechanistic insight into the neuroprotective effects of the ethyl acetate fraction of *P. orientalis* (EAPO) under diabetic-mimicking conditions. Specifically, this study evaluates its cytotoxicity and examines how AMPK modulation contributes to its neuroprotective and anti-neurodegenerative effects. This study hypothesizes that EAPO could mitigate high-glucose–induced cytotoxicity and enhance neuroprotection in rMc-1 via AMPK activation.

## 2 Materials and methods

### 2.1 Chemicals

The chemical reagents used in this study are listed in the Supplementary Material, supplement 1 - 3.

### 2.2 Plant sample collection and extraction

*P. orientalis* (L.) Franco has been taxonomically validated with International Plant Index (2022) on 11th January 2022. Plant authentication was conducted by a qualified botanist, and voucher specimen (KLU50129) was deposited at the Rimba Ilmu Botanic Garden Herbarium in University of Malaya. The leaves samples collected from Selangor, Malaysia, were rinsed with distilled water prior to shade dry at room temperature. The dried plant samples were ground into coarse powders and subsequently subjected to solvent partitioning as described by Ho et al. (2022). Briefly, sample coarse powders were macerated with absolute methanol in a solid to solvent ratio (w/v) of 1:5 for 3 days, followed by filtration and solvent removal under vacuum. The methanolic crude extract of *P. orientalis* was reconstituted in distilled water and sequentially partitioned using solvents of increasing polarity: hexane, chloroform, ethyl acetate, n-butanol, and distilled water in a 1:1 (v/v) ratio. Among the six resulting fractions, the EAPO was selected for the present mechanistic investigation, based on our previous findings demonstrating its potent *in vitro* anti-diabetic, anti-glycation, and anti-inflammatory activities relevant to DR (Ho et al., 2022).

### 2.3 Phytochemical identification via liquid chromatography-quadrupole/time-of-flight mass spectrometry (LC-QToF-MS)

The phytochemical profiling of EAPO was performed using an Agilent 1290 Infinity LC system coupled with an Agilent 6520 Accurate-Mass QToF mass spectrometer, operating in both positive and negative electrospray ionization (ESI) modes. Polar metabolites were further characterized via liquid chromatography–quadrupole time-of-flight mass spectrometry (LC-QToF-MS) using the same LC platform. Full-scan data were acquired in the m/z range of 100–3,200, with a fragmentation voltage of 125 V and an ion source temperature of 300 °C. A 0.2 µL sample volume was injected onto an Agilent ZORBAX Eclipse XDB-C18 narrow-bore column (150 mm × 2.1 mm, 3.5 µm; P/N 930,990-902), maintained at 25 °C, with the autosampler set at 4 °C. Chromatographic separation was achieved using a binary gradient of water (solvent A) and acetonitrile (solvent B), both containing 0.1% formic acid, at a flow rate of 0.5 mL/min over 30 min. The gradient began with 5% B for the first 20 min, followed by a linear increase to 100% B from 20 to 30 min. Data acquisition and processing were conducted using Agilent MassHunter software (vB.07.00), and metabolite identification was confirmed by comparison with entries in the METLIN database. Compound identification was performed using database (Db) and Molecular Formula Generator (MFG) scores, with only candidates scoring close to 100 and exhibiting mass errors within −2 to +2 ppm selected for further analysis. A mass accuracy threshold of ±5 ppm and a signal-to-noise ratio (S/N) ≥ 10 were applied. Isotopic distributions and MS/MS fragmentation patterns were matched against theoretical values to support compound assignment. Relative quantification was based on extracted ion chromatogram peak areas and is reported as semi-quantitative due to the absence of internal standards.

### 2.4 rMc-1 culture and cytotoxicity study

Immortalized rat retinal Müller cells (rMc-1; Catalog No. ENW001) were acquired from Kerafast (Boston, Massachusetts, United States). The cells were grown in a T75 flask with Dulbecco’s Modified Eagle Medium (DMEM) containing 5.5 mM glucose with 2.2 mM L-Glutamine, 10% fetal bovine serum and 1% penicillin-streptomycin at 37 °C under a humidified atmosphere of 5% carbon dioxide. The rMc-1 were routinely screened using PCR-based assays to confirm that they were free of *mycoplasma* contamination. The rMc-1 at passage 10 to 12 were utilized and serum-starved in serum-free media for 24 h prior to the cytotoxicity evaluation. To determine the half-maximal inhibitory concentration (IC_50_, mg/mL) and the maximum tolerable concentration of EAPO for subsequent experiments, rMc-1 were exposed to a series of ten concentrations of EAPO. 3-(4,5-dimethylthiazol-2-yl)-2,5-diphenyl tetrazolium bromide (MTT) assay was used to evaluate the viability of rMc-1 (Ho et al., 2022). The IC_50_ value expressed in mg/mL, was derived from the percentage of cell viability and cell death. The cell viability (%) and cell death (%) were calculated as follows:

$$\left(i\right)\text{ Cell viability }\%=\frac{{Absorbance}_{sampletreatedgroup}}{{Absorbance}_{untreatedcontrolgroup}}\times 100$$

$$\left(\text{ii}\right)\text{ Cell death }\%=\frac{\left({Absorbance}_{untreatedcontrolgroup}\;‐\;{Absorbance}_{sampletreatedgroup}\right)}{{Absorbance}_{untreatedcontrolgroup}}\times 100$$

Absorbance _sample treated group_ and Absorbance _untreated control group_ refer to respective absorbance of the EAPO-treated group and negative control group.

### 2.5 *In vitro* rMc-1 treatment

Based on the maximum tolerable concentration identified from the MTT assay, rMc-1 control and treatment groups were exposed to three concentrations of EAPO to evaluate the dose-response effect. Briefly, rMc-1 (2.0 × 10^4^ cells) were seeded and serum-starved in glucose-containing serum-free media at concentrations of 5.5 mM, 25 mM, and 50 mM for 24 h. Following this, the cells were treated with serial dilutions of EAPO (0.03125, 0.0625, and 0.125 mg/mL) for either 24 or 48 h. In a separate experimental setup, another set of rMc-1 were pre-treated with either an AMPK inhibitor (compound C, 10 μmol/L) or an AMPK activator (AICAR, 1 mmol/L; Sigma-Aldrich) for 1 h (Chen et al., 2018), followed by co-incubation with EAPO and varying glucose concentrations for 48 h.

### 2.6 Gene expression

#### 2.6.1 Ribonucleic acid (RNA) extraction

The RNA in rMc-1 was extracted with TRIzol reagent according to the manufacturer’s protocol (T9424, Sigma-Aldrich). The extracted RNA samples (purity: A260/A230 and A260/A280 > 2.0) were treated with deoxyribonuclease (M6101, Promega, U.S) and stored at −80 °C until further analysis.

#### 2.6.2 Reverse transcription-quantitative polymerase chain reaction

Complementary deoxyribonucleic acids (cDNA) were synthesized from the extracted RNA samples with High-Capacity cDNA reverse transcription kit (4,368,814, Thermo Fisher Scientific). The gene expression of *VEGF, VEGR2, mTOR, AKT1, Raf-1, MEK1* and *MEK 2* were quantified with 1 µg cDNA template, respective gene-specific forward and reverse primers (Supplementary Material, Supplement 2) and SYBR green master mix reagent kit (BIO-98005, Meridian). The relative expression level of each gene marker was derived through a 2^−ΔΔCT^ method and normalized with a housekeeping gene, β-actin.

### 2.7 Protein expression

#### 2.7.1 Whole cell protein lysate

The extraction of whole cell protein lysate from rMc-1 was conducted according to the method by Shatzkes et al. (2014) with minor modifications. Briefly, rMc-1 at 80%–90% confluence were harvested by scraping and centrifuged at 4 °C for 15 min. The cell pellets were lysed with ice-cold lysis buffer containing 70 mM Tris (pH 7.4), 160 mM sodium chloride, 1 mM ethylenediaminetetraacetic acid (pH 8.0), 1% Triton X-100 and 1X protease inhibitor (11,697,498,001, Roche, Switzerland). Supernatant containing the whole cell lysate protein was stored at −80 °C until further analysis.

#### 2.7.2 Cytoplasmic and nuclear fraction

The protein in the nucleus and cytoplasm of rMc-1 were extracted according to the protocol of NE-PER™ Nuclear and Cytoplasmic Extraction kit (78,833, Thermo Fisher Scientific, U.S.). The secreted proteins of rMc-1 were isolated with cytoplasmic extraction reagent I and II. Insoluble (pellet) fractions containing the nuclei were extracted with ice-cold nuclear extraction reagent for the nuclei fractions. The extracted protein supernatants were then stored at −80 °C until further analysis.

#### 2.7.3 Protein quantification

The total protein content in the whole cell lysate, cytoplasmic and nuclear fractions of rMc-1 were quantified with bicinchoninic acid method according to the manufacturer’s manual (23,225, Thermo Fisher Scientific, US). Bovine serum albumin was used as the standard (0–2000 μg/mL). The final concentration of protein was expressed as microgram per milliliter.

#### 2.7.4 Sodium dodecyl sulfate–polyacrylamide gel electrophoresis (SDS-PAGE) and Western blot

The proteins of whole cell lysate, cytoplasmic and nuclear fractions for rMc-1 (30 μg each) were separated by SDS-PAGE according to Nowakowski et al. (2014) with minor modifications. Briefly, protein samples were denatured by Orange G loading dye (928-40004, LI-COR, U.S.) supplemented with 10% β-mercaptoethanol by heating at 95 °C and subsequently separated on a 12% resolving gel. Following gel electrophoresis, proteins were then transferred onto a 0.45 µm polyvinylidene difluoride membrane (Immobilon, Millipore). The membranes were blocked for 1 h in PBS containing 5% non-fat dry milk and 0.1% Tween® 20 at room temperature. The blocked membranes were incubated overnight at 4 °C with appropriate primary antibodies (Supplementary Material, Supplement 3). In the following day, all membranes were further incubated with horseradish peroxidase (HRP)-conjugated secondary antibodies. Protein bands were visualized with chemiluminescent substrates under signal accumulation mode in a Bio-Rad ChemiDoc MP imaging system (California, United States). Densitometric analysis of gel bands was performed using ImageJ software (National Institute of Health, Maryland, United States). The relative expression levels of each protein marker were quantified as fold changes normalized to the internal controls, β-actin and glyceraldehyde-3-phosphate dehydrogenase.

### 2.8 Statistical analysis

All quantitative experimental data were presented as mean ± standard deviation (n = 3). Different alphabetical letters denote statistically significant differences (p < 0.05) among experimental groups, as determined by one-way ANOVA followed by Tukey’s *post hoc* test. Groups labeled with the same alphabetical letters are not significantly different from each other. A p-value of less than 0.05 was considered statistically significant. All statistical analysis was performed with GraphPad Prism® (version 9.5.1) software.

## 3 Results

### 3.1 Phytochemicals in the EAPO

LC–QToF–MS analysis of EAPO, based on chromatograms obtained under both positive and negative ionization modes, revealed distinct peaks corresponding to five categories of bioactive metabolites: monoterpene esters, diterpenoids, saturated fatty acids, phenolic glycosides, and chlorophyll (Table 1; Supplementary Material, supplement 4, 5). Monoterpene ester was represented by 4Z-decenyl acetate, which constituted the highest proportion (1.88%) among the identified compounds. Diterpenoids formed another major phytochemical group, including ferruginol (1.31%), isosteviol methyl ester (0.97%), and thunbergol (0.35%). In addition, saturated fatty acids such as 2R-hydroxylauric acid (0.97%) and the phenolic glycoside sphalleroside A (0.78%) were detected in EAPO. A minor fraction of chlorophyll derivative, 2,6-nonadien-1-ol (0.44%), was also present. Collectively, these results indicated that EAPO was enriched with structurally diverse secondary metabolites, particularly diterpenoids and phenolic glycosides, which may contribute to the anti-neurodegenerative action of *P. orientalis*.

**TABLE 1:** The phytochemicals of *Platycladus orientalis* ethyl acetate fraction identified by LC-QToF-MS.

Phytochemical category	Bioactive compounds	Composition (%)
Chlorophyll	2,6-Nonadien-1-ol	0.44
Phenolic glycoside	Sphalleroside A	0.78
Saturated fatty acid	2R-hydroxylauric acid	0.97
Diterpenoids	Thunbergol Isosteviol methyl ester Ferruginol	0.35 0.97 1.31
Monoterpene ester	4Z-decenyl acetate	1.88

### 3.2 Cytoprotective effect of EAPO on rMc-1 against glucose stress

**FIGURE 1:**
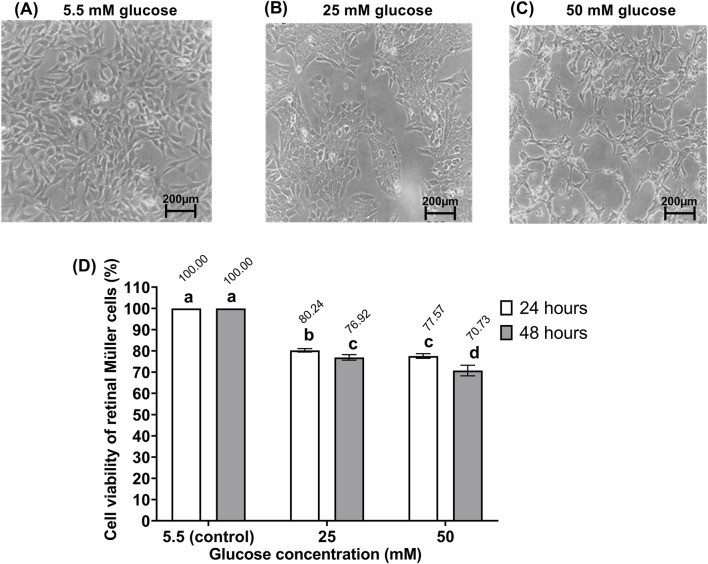
Morphology of retinal Müller cells (rMc-1) after 48 h of exposure to **(A)** 5.5 mM, **(B)** 25 mM, and **(C)** 50 mM glucose. Magnification power ×20. **(D)** Cell viability of rMc-1 cultured in 5.5 mM, 25 mM, and 50 mM glucose for 24 and 48 h, presented as mean ± standard deviation from three independent experiments (n = 3). Different letters indicate statistically significant differences (p < 0.05) among the experimental groups (24 h and 48 h), as determined by one-way ANOVA followed by Tukey’s *post hoc* test. Groups that share the same letter are not significantly different.

Figure 1 illustrates the morphological changes and viability of rMc-1 exposed to three different glucose concentrations (5.5, 25, 50 mM) over 24 and 48 h. Under normal glucose (5.5 mM), rMc-1 retained their typical spindle-shaped or polygonal morphology with centrally located nuclei and showed no significant change in viability between 24 and 48 h (Figure 1A). In contrast, high-glucose exposure induced both morphological abnormalities and time-dependent reductions in cell viability. At 25 mM glucose, rMc-1 displayed disintegration and a disordered arrangement (Figure 1B), with viability declining from 24 to 48 h, reaching 76.92% at 48 h. At 50 mM glucose, cell bodies became enlarged, fused into block-like structures, and exhibited marked misalignment (Figure 1C), accompanied by a further viability reduction to 70.73% at 48 h when compared to the normal glucose (5.5 mM) control.

**FIGURE 2:**
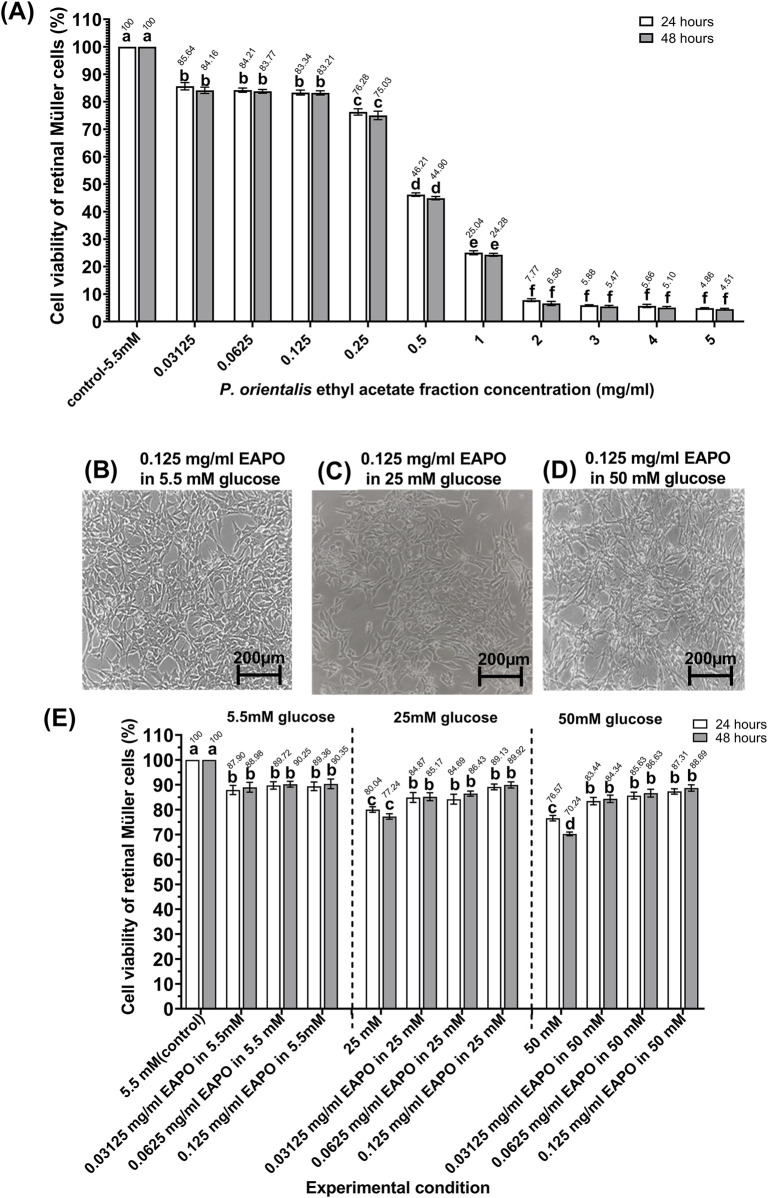
**(A)** Cell viability of rMc-1 treated with ten concentrations of EAPO under 5.5 mM glucose. **(B–D)** Cell morphology of rMc-1 after 48 h of EAPO treatment under **(B)** 5.5 mM, **(C)** 25 mM, and **(D)** 50 mM glucose. **(E)** Cell viability of rMc-1 treated with three serial dilutions of EAPO under 5.5 mM, 25 mM, and 50 mM glucose for 24 and 48 h. Data were expressed as mean ± standard deviation of three independent determinations (n = 3). Different letters indicate statistically significant differences (p < 0.05) among experimental groups (24 h and 48 h), based on one-way ANOVA followed by Tukey’s *post hoc* test; groups sharing the same letter are not significantly different. EAPO, ethyl acetate fraction of *Platycladus orientalis*. † The IC_50_ for standard potassium dichromate were 0.336 mg/mL and 0.292 mg/mL after respective 24-h and 48-h treatments.

Cytotoxicity analysis showed that the IC_50_ of EAPO in rMc-1 under normal glucose (5.5 mM) at 48 h was 0.323 mg/mL (Figure 2A). No significant differences in viability were observed between 24 and 48 h across the tested concentrations. However, treatment with 0.25 mg/mL EAPO reduced the rMc-1 viability to below 80%, while 0.5 mg/mL further decreased rMc-1 viability to below 50% (Figure 2A). The MTT assay identified 0.125 mg/mL as the maximum non-cytotoxic concentration, maintaining rMc-1 viability above 80%. Therefore, subsequent experiments under normal, high- and severely high-glucose conditions were conducted using serially diluted, non-cytotoxic concentrations of 0.03125, 0.0625, and 0.125 mg/mL EAPO.

Figures 2B–E illustrates the morphological and dose-dependent cytoprotective effects of EAPO on rMc-1 exposed to high-glucose stress. Under normal glucose, EAPO treatment maintained rMc-1 viability at nearly 90%. (Figures 2B,E). After 48 h of treatment, EAPO maintained rMc-1 viability above 80% under both 25 mM and 50 mM glucose conditions, relative to the normal glucose (5.5 mM) control (Figure 2E). At the lowest concentration (0.03125 mg/mL), EAPO improved rMc-1 viability by 10% and 20% when compared with untreated controls at 25 mM and 50 mM glucose, respectively (Figure 2E). At the highest concentration tested (0.125 mg/mL), rMc-1 viability was restored to 89.9% under 25 mM glucose and 88.7% under 50 mM glucose (Figure 2E), demonstrating the robust neuroprotective activity of EAPO against glucose-induced cytotoxicity and reactive gliosis.

### 3.3 Effects of glucose stress and EAPO on neurodegenerative gene marker expression in rMc-1

**FIGURE 3:**
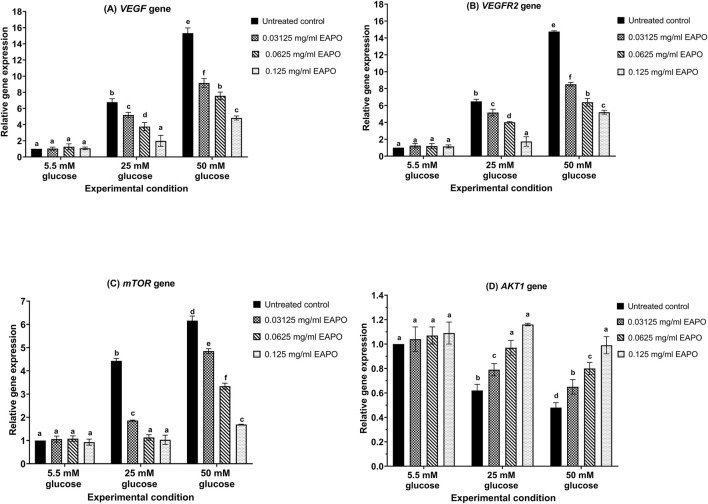
The gene expression of **(A)** *VEGF*, **(B)** *VEGFR2*, **(C)** *mTOR* and **(D)** *AKT1* in retinal Müller cells (rMc-1) treated with EAPO under 5.5 mM, 25 mM, and 50 mM glucose for 48 h. Data were expressed as mean ± standard deviation of three independent determinations (n = 3). *AKT,* serine/threonine kinase 1; *mTOR,* mammalian target of rapamycin; EAPO, ethyl acetate fraction of *Platycladus orientalis*; *VEGF,* vascular endothelial growth factor; *VEGFR2*, vascular endothelial growth factor receptor 2. *AKT1*, serine/threonine kinase 1; *mTOR,* mammalian target of rapamycin; EAPO, ethyl acetate fraction of *Platycladus orientalis*; *VEGF,* vascular endothelial growth factor; *VEGFR2*, vascular endothelial growth factor receptor 2.

**FIGURE 4:**
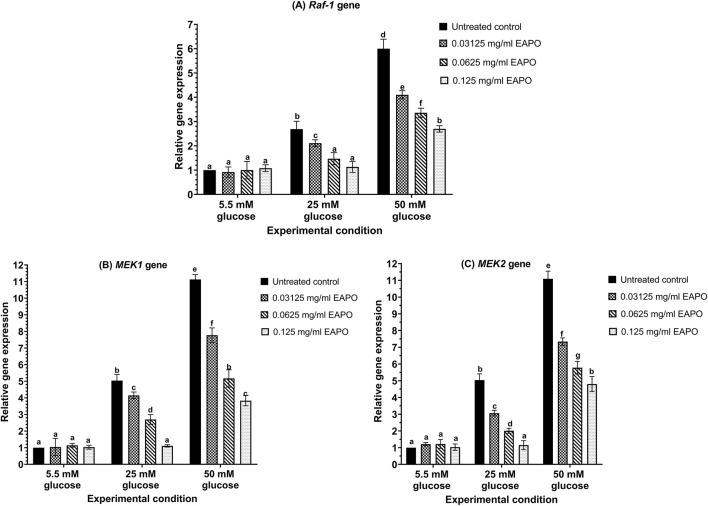
The gene expression of **(A)** *Raf-1,* **(B)** *MEK1* and **(C)** *MEK2* in retinal Müller cells (rMc-1) treated with EAPO under 5.5 mM, 25 mM, and 50 mM glucose for 48 h. Data were expressed as mean ± standard deviation of three independent determinations (n = 3). *MEK*, mitogen-activated extracellular kinase; EAPO, ethyl acetate fraction of *Platycladus orientalis*; *Raf-1*, rapidly accelerated fibrosarcoma-1.

High-glucose stress markedly altered the expression of neurodegeneration-related genes in rMc-1 (Figures 3, 4). *VEGF* expression increased 6.78-fold at 25 mM glucose and further to 15.32-fold at 50 mM when compared to the normal glucose (5.5 mM) control (Figure 3A). A similar trend was observed for *VEGFR2*, which rose 6.49-fold and 14.75-fold under 25 mM and 50 mM glucose, respectively (Figure 3B). The expression of *mTOR* was also upregulated, showing a 4.43-fold increase at 25 mM and a 6.16-fold increase at 50 mM (Figure 3C). In contrast, *AKT1* expression was progressively downregulated, declining to 0.62-fold at 25 mM and 0.48-fold at 50 mM (Figure 3D). *Raf-1* expression increased by 2.69-fold under 25 mM glucose and 6.00-fold under 50 mM glucose (Figure 4A). Similarly, *MEK1* and *MEK2* showed pronounced upregulation, with *MEK1* rising from 5.29-fold to 11.13-fold and *MEK2* from 5.04-fold to 11.09-fold across the two glucose concentrations (Figures 4B,C).

The gene expression of neurodegeneration-associated biomarkers in rMc-1 exposed to high (25 mM) and severely high (50 mM) glucose was modulated by EAPO in a concentration-dependent manner (Figures 3, 4). At 0.125 mg/mL, EAPO significantly downregulated *VEGF, VEGFR2, MEK1, and MEK2* at 25 mM glucose (Figures 3A,B, 4B,C), restoring their expression to levels comparable to the normal glucose (5.5 mM) control. Even at 0.03125 mg/mL, EAPO markedly suppressed gene expression at 50 mM glucose, reducing *VEGF* and *VEGFR2* by 40% and 42% (Figures 3A,B), as well as *MEK1* and *MEK2* by 30% and 34% (Figures 3A,B, 4B,C), respectively. At 0.0625 mg/mL, EAPO effectively decreased *mTOR* and *Raf-1* expression (Figures 3C, 4A) under 25 mM glucose and restored *AKT1* expression (Figure 3D) to baseline levels. Notably, EAPO treatment did not affect the expression of *VEGF, VEGFR2, AKT1, mTOR, Raf-1,* or *MEK1/2* in rMc-1 maintained under normal glucose condition (Figures 3, 4).

### 3.4 Effects of glucose stress and EAPO treatment on neurodegenerative protein marker expression in rMc-1

**FIGURE 5:**
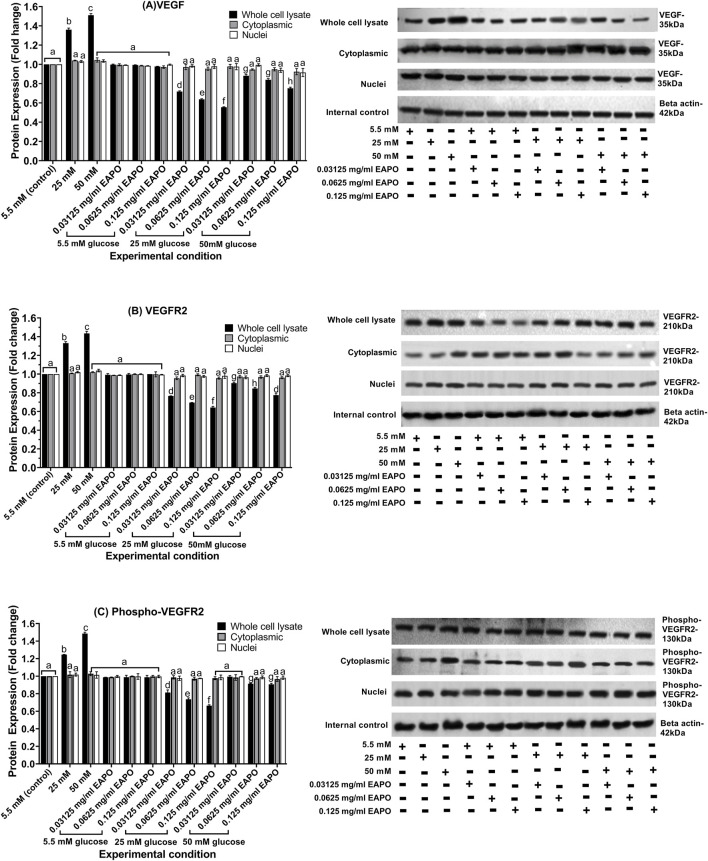
Representative Western blots showing the expression of **(A)** VEGF, **(B)** VEGFR2, and **(C)** phospho-VEGFR2 in retinal Müller cells (rMc-1) treated with EAPO under 5.5 mM, 25 mM, and 50 mM glucose for 48 h. Graphical data are presented as fold changes in protein expression and expressed as mean ± standard deviation (n = 3). EAPO, ethyl acetate fraction of *Platycladus orientalis*; VEGF, vascular endothelial growth factor; VEGFR2, vascular endothelial growth factor receptor 2.

Consistent with the gene expression findings, Western blot analysis confirmed a significant increase in VEGF protein levels in rMc-1 exposed to glucose stress, rising 1.37-fold at 25 mM glucose and 1.52-fold at 50 mM glucose when compared to the normal glucose (5.5 mM) control (Figure 5A). Similarly, VEGFR2 expression increased to 1.33-fold and 1.44-fold under 25 mM and 50 mM glucose, respectively (Figure 5B). Phosphorylated VEGFR2 (phospho-VEGFR2) also showed a marked elevation, increasing from 1.25-fold at 25 mM glucose to 1.44-fold at 50 mM glucose (Figure 5C). EAPO treatment markedly attenuated these elevations in a dose-dependent manner. At the lowest concentration (0.03125 mg/mL), both VEGF and VEGFR2 expression were reduced to <0.75-fold under 25 mM glucose and <0.90-fold under 50 mM glucose. At 0.125 mg/mL, EAPO decreased VEGF protein expression by 59% and 50% and VEGFR2 by 52% and 46% under 25 mM and 50 mM glucose, respectively (Figures 5A,B). A similar trend was observed for phospho-VEGFR2, with reductions of 47% (25 mM glucose) and 39% (50 mM glucose) at 0.125 mg/mL EAPO. Even at 0.03125 mg/mL, phospho-VEGFR2 expression was suppressed below baseline levels, reaching <0.85-fold under 25 mM and <1.0-fold under 50 mM glucose (Figure 5C).

**FIGURE 6:**
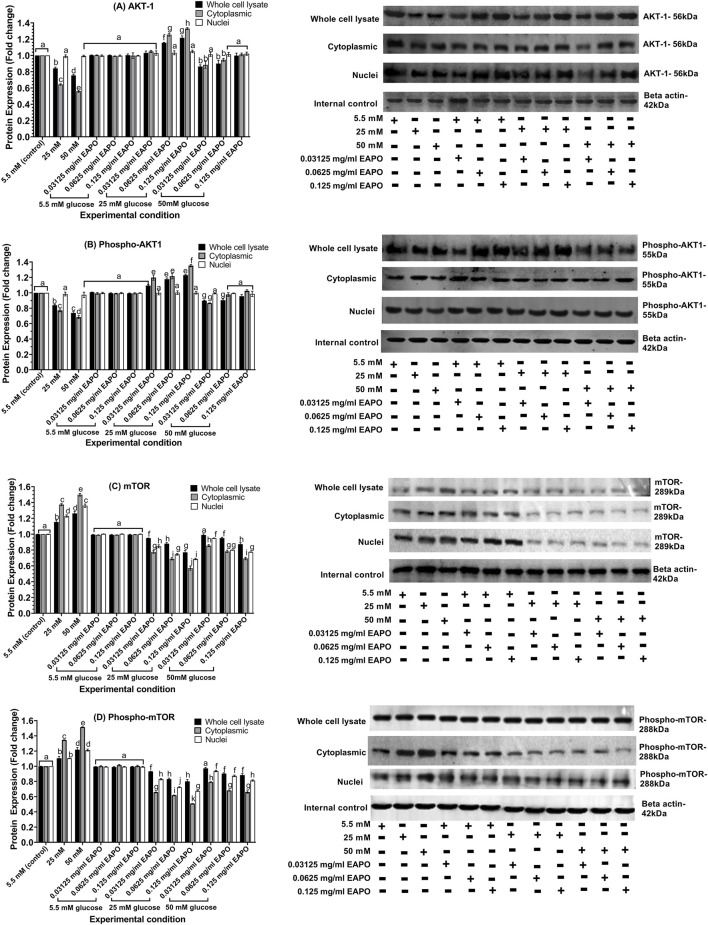
Representative Western blots showing the expression of **(A)** AKT1, **(B)** phospho-AKT1, **(C)** mTOR and **(D)** phospho-mTOR in retinal Müller cells (rMc-1) treated with EAPO under 5.5 mM, 25 mM, and 50 mM glucose for 48 h. Graphical data are presented as fold changes in protein expression and expressed as mean ± standard deviation (n = 3). AKT1, serine/threonine kinase 1; EAPO, ethyl acetate fraction of *Platycladus orientalis*; mTOR, mammalian target of rapamycin.

AKT1 protein expression in rMc-1 was markedly reduced by glucose stress, with more pronounced decreased in the cytoplasmic fraction (0.64- and 0.56-fold at 25 mM and 50 mM glucose, respectively) when compared to the whole cell lysates (0.84- and 0.75-fold) (Figure 6A). Phosphorylated AKT1 (phospho-AKT1) showed a similar pattern, declining to 0.77- and 0.69-fold in the cytoplasmic fraction and to 0.84- and 0.79-fold in whole cell lysates under high glucose concentration (Figure 6B). These findings indicated that hyperglycemic stress suppressed AKT1 signaling alongside elevated VEGF activation, potentially driving gliotic responses in rMc-1.

EAPO treatment restored AKT1 expression in a concentration-dependent manner. At 25 mM glucose, even the lowest concentration (0.03125 mg/mL) was sufficient to normalize AKT1 and phospho-AKT1 levels in both cytoplasmic and whole-cell fractions. Higher EAPO concentrations (0.0625–0.125 mg/mL) further enhanced AKT1 expression, increasing it to 1.16–1.22-fold in whole-cell lysates and 1.25–1.33-fold in cytoplasmic fractions. In contrast, the restorative effect of EAPO was limited at 50 mM glucose, with only the highest concentration (0.125 mg/mL) significantly restoring phospho-AKT1 levels in both cytoplasmic and whole-cell fractions (Figures 6A,B).

Glucose stress markedly increased mTOR protein expression in rMC-1 cells, with the most pronounced elevation observed in the cytoplasmic fraction. Cytoplasmic mTOR levels rose to 1.37-fold under 25 mM glucose and further to 1.50-fold under 50 mM glucose compared with the normal glucose (5.5 mM) control. In contrast, nuclear mTOR expression showed a more moderate increase, reaching 1.23-fold and 1.36-fold at 25 mM and 50 mM glucose, respectively, while whole-cell lysates exhibited only slight increases (1.16–1.26-fold) (Figure 6C). Phosphorylated mTOR (phospho-mTOR) followed a similar glucose-dependent pattern. Cytoplasmic phospho-mTOR increased from 1.35-fold at 25 mM glucose to 1.51-fold at 50 mM glucose whereas nuclear fractions showed only modest elevations (1.10–1.21-fold). Whole-cell lysates displayed a comparable minor increase in phospho-mTOR across both glucose concentrations (Figure 6D).

**FIGURE 7:**
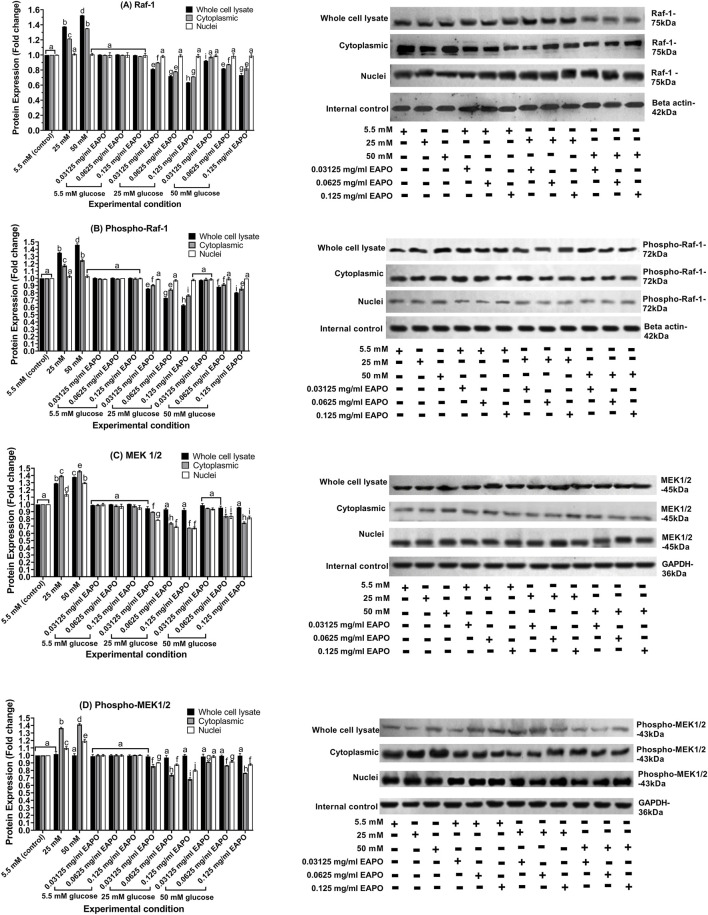
Representative Western blots showing the expression of **(A)** Raf-1, **(B)** phospho-Raf-1, **(C)** MEK1/2 and **(D)** phospho-MEK1/2 in retinal Müller cells (rMc-1) treated with EAPO under 5.5 mM, 25 mM, and 50 mM glucose for 48 h. Graphical data are presented as fold changes in protein expression and expressed as mean ± standard deviation (n = 3). EAPO, ethyl acetate fraction of *Platycladus orientalis*; MEK, mitogen-activated extracellular kinase; Raf-1, rapidly accelerated fibrosarcoma-1.

EAPO treatment differentially regulated mTOR and phospho-mTOR proteins across cellular compartments. Cytoplasmic mTOR was the most sensitive to inhibition, with 0.125 mg/mL EAPO reducing levels to 0.57–0.69-fold, compared with 0.69–0.76-fold in the nucleus and 0.77–0.87-fold in whole-cell lysates (Figure 6C). Even the lowest concentration (0.03125 mg/mL) suppressed cytoplasmic mTOR to below 0.78-fold under 25 mM glucose, whereas higher concentrations (0.0625–0.125 mg/mL) were required to achieve comparable suppression in the nuclear and whole-cell fractions. For phospho-mTOR, 0.0625 mg/mL EAPO effectively reduced expression under 25 mM glucose to below 0.65-fold in the nucleus, 0.75-fold in the cytoplasm, and 0.85-fold in whole-cell lysates (Figure 6D). Under 50 mM glucose, cytoplasmic and whole-cell phospho-mTOR levels were similarly reduced at 0.0625 mg/mL and 0.125 mg/mL EAPO, whereas nuclear suppression plateaued between 0.03125 and 0.0625 mg/mL. Glucose stress increased the levels of Raf-1 and its phosphorylated form (phospho-Raf-1) in both cytoplasmic and whole-cell compartments. In whole-cell lysates, Raf-1 expression rose to 1.38-fold at 25 mM glucose and 1.52-fold at 50 mM glucose. In the cytoplasmic fraction, it increased to 1.22-fold and 1.35-fold, respectively (Figure 7A). Phospho-Raf-1 showed a more modest rise in the cytoplasm (1.17–1.25-fold) but stronger upregulation in whole-cell lysates (1.35–1.46-fold) (Figure 7B). EAPO treatment significantly attenuated these effects in a dose-dependent manner. At 0.125 mg/mL, Raf-1 expression was reduced by 54% and 51% in whole-cell lysates and by 42% and 39% in cytoplasmic fractions at 25 mM and 50 mM glucose, respectively. Likewise, phospho-Raf-1 levels decreased by 40%–49% in whole-cell lysates and by 31%–35% in cytoplasmic fractions under both glucose conditions.

High and severely high glucose stress substantially increased MEK1/2 and phosphorylated MEK1/2 (phospho-MEK1/2) protein levels in all rMC-1 cellular compartments (Figures 7C,D). In the cytoplasm, MEK1/2 and phospho-MEK1/2 rose to 1.39- and 1.37-fold at 25 mM glucose, increasing further to 1.46- and 1.42-fold at 50 mM glucose. Whole-cell lysates showed moderate increases, with MEK1/2 reaching 1.29- and 1.38-fold and phospho-MEK1/2 rising slightly to 1.02- and 1.03-fold at 25 mM and 50 mM glucose, respectively. The nuclear fractions exhibited the smallest changes, with MEK1/2 increasing to 1.14- and 1.30-fold and phospho-MEK1/2 to 1.10- and 1.19-fold under the same conditions. These results indicated that glucose stress activated the MEK/ERK and mTOR pathways in a dose- and compartment-specific manner. EAPO treatment reduced these glucose-induced increases in a concentration-dependent manner. In the cytoplasm, MEK1/2 expression decreased by 36% (0.03125 mg/mL), 47% (0.0625 mg/mL), and 52% (0.125 mg/mL) under 25 mM glucose, and by 35%, 42%, and 48% under 50 mM glucose, with similar dose-dependent reductions observed for phospho-MEK1/2 (37%–50% at 25 mM and 35%–45% at 50 mM). Nuclear MEK1/2 and phospho-MEK1/2 were significantly reduced only at concentrations ≥0.0625 mg/mL, while whole-cell lysate levels were restored close to those of the normal glucose (5.5 mM) controls across all tested dose. Importantly, EAPO did not alter Raf-1, MEK1/2, phospho-Raf-1, or phospho-MEK1/2 expression under normal glucose (5.5 mM), underscoring its selective activity under hyperglycemic stress.

### 3.5 Neurodegeneration markers in rMc-1 following the AMPK activation or inhibition under different glucose and EAPO treatment

**FIGURE 8:**
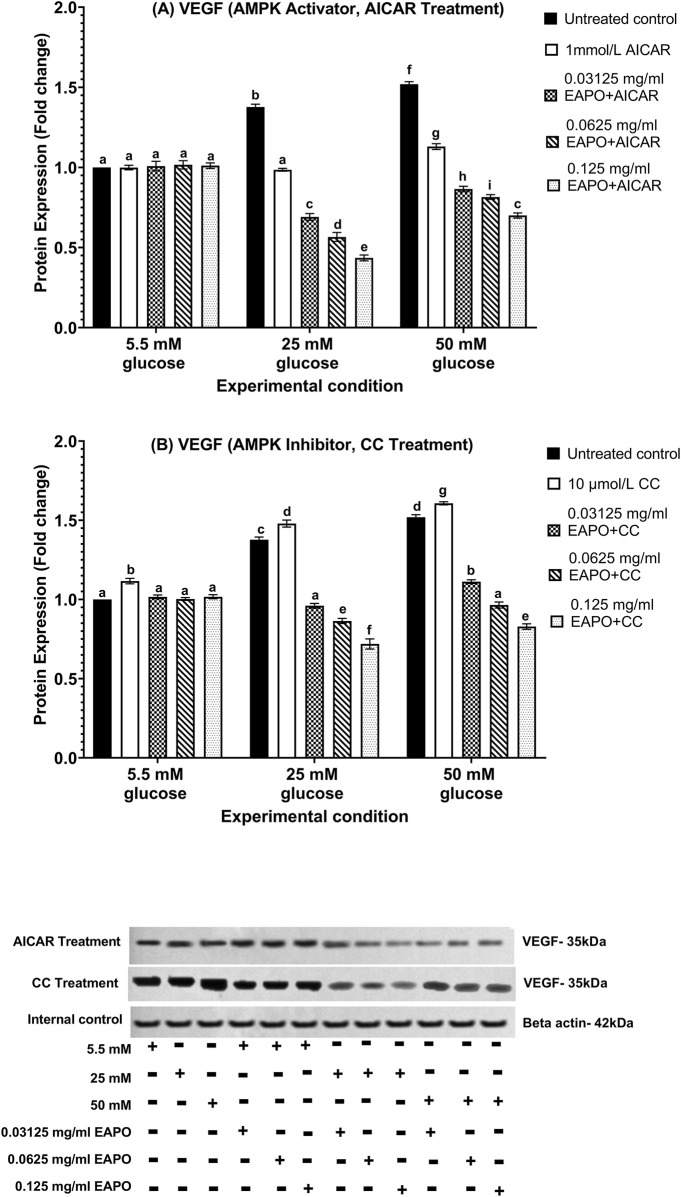
Representative Western blots showing VEGF protein expression in retinal Müller cells (rMc-1) treated with EAPO in combination with **(A)** the AMPK activator AICAR and **(B)** the AMPK inhibitor Compound C under 5.5 mM, 25 mM, and 50 mM glucose concentrations for 48 h. Untreated control refers to rMc-1 cultured under the indicated glucose concentration without EAPO or AMPK modulators (AICAR or CC). The (+/−) symbols beneath the blots denote the presence (+) or absence (−) of each experimental variable, including glucose level (5.5, 25, or 50 mM), and EAPO concentration. Graphical data are presented as fold changes in protein expression and expressed as mean ± standard deviation (n = 3). CC, compound C; EAPO, ethyl acetate fraction of *Platycladus orientalis*; VEGF, vascular endothelial growth factor.

**FIGURE 9:**
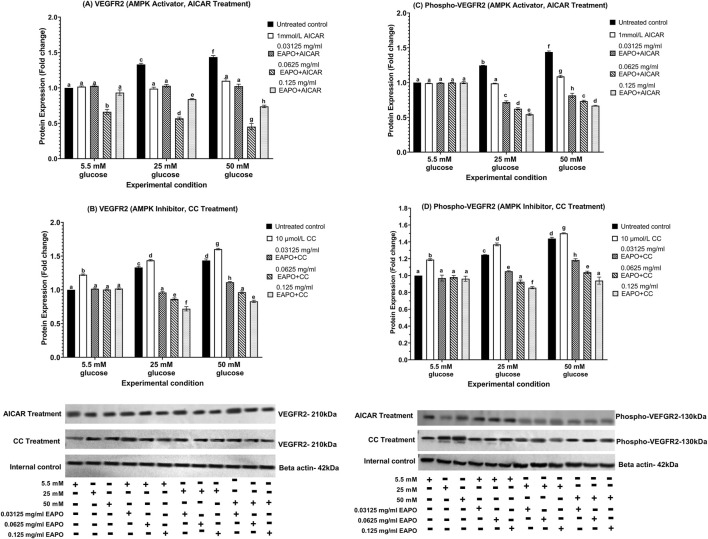
Representative Western blots showing rMc-1 expression of **(A)** VEGFR2 in cells treated with EAPO in combination with the AMPK activator, AICAR, **(B)** VEGFR2 in cells treated with EAPO in combination with the AMPK inhibitor, Compound C, **(C)** phospho-VEGFR2 in cells treated with EAPO and AICAR, and **(D)** phospho-VEGFR2 in cells treated with EAPO and Compound C under 5.5 mM, 25 mM, and 50 mM glucose for 48 h. Untreated control refers to rMc-1 cultured under the indicated glucose concentration without EAPO or AMPK modulators (AICAR or CC). The (+/−) symbols beneath the blots denote the presence (+) or absence (−) of each experimental variable, including glucose level (5.5, 25, or 50 mM), and EAPO concentration. Graphical data are presented as fold changes in protein expression and expressed as mean ± standard deviation (n = 3). CC, compound C, EAPO, ethyl acetate fraction of *Platycladus orientalis*; VEGFR2, vascular endothelial growth factor receptor 2.

**FIGURE 10:**
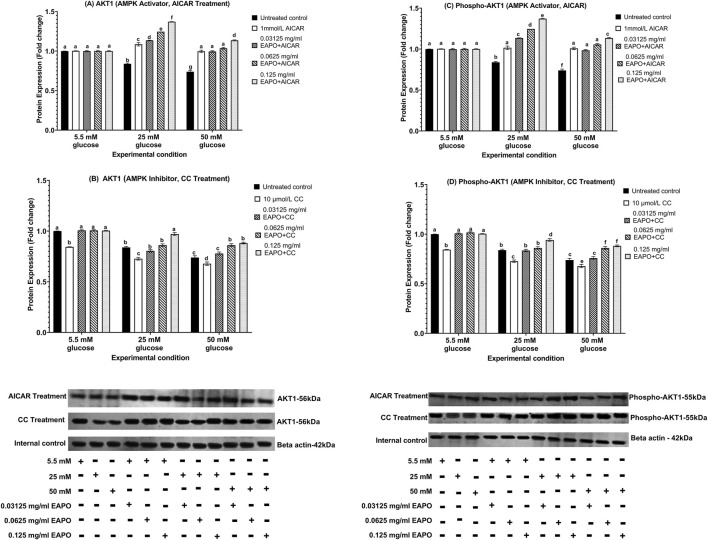
Representative Western blots showing rMc-1 expression of **(A)** AKT1 in cells treated with EAPO in combination with the AMPK activator, AICAR, **(B)** AKT1 in cells treated with EAPO in combination with the AMPK inhibitor, Compound C, **(C)** phospho-AKT1 in cells treated with EAPO and AICAR, and **(D)** phospho-AKT1 in cells treated with EAPO and Compound C under 5.5 mM, 25 mM, and 50 mM glucose for 48 h. Untreated control refers to rMc-1 cultured under the indicated glucose concentration without EAPO or AMPK modulators (AICAR or CC). The (+/−) symbols beneath the blots denote the presence (+) or absence (−) of each experimental variable, including glucose level (5.5, 25, or 50 mM), and EAPO concentration. Graphical data are presented as fold changes in protein expression and expressed as mean ± standard deviation (n = 3). CC, compound C; EAPO, ethyl acetate fraction of *Platycladus orientalis*; AKT1, serine/threonine kinase 1.

**FIGURE 11:**
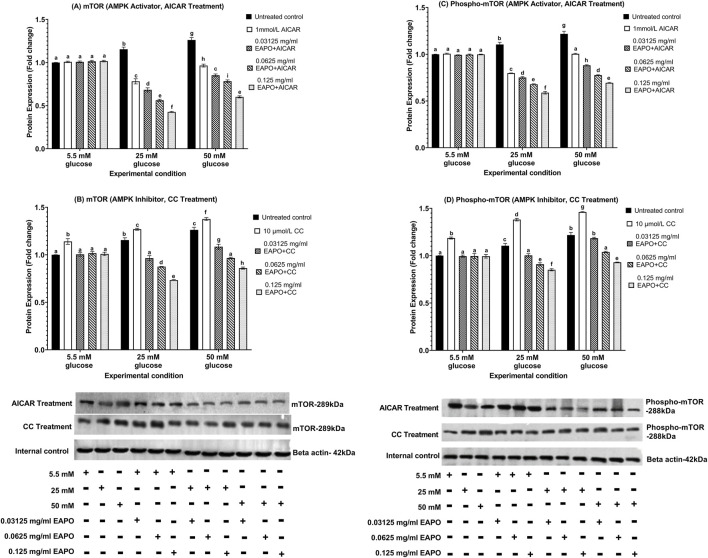
Representative Western blots showing rMc-1 expression of **(A)** mTOR in cells treated with EAPO in combination with the AMPK activator, AICAR, **(B)** mTOR in cells treated with EAPO in combination with the AMPK inhibitor, Compound C, **(C)** phospho-mTOR in cells treated with EAPO and AICAR, and **(D)** phospho-mTOR in cells treated with EAPO and Compound C under 5.5 mM, 25 mM, and 50 mM glucose for 48 h. Untreated control refers to rMc-1 cultured under the indicated glucose concentration without EAPO or AMPK modulators (AICAR or CC). The (+/−) symbols beneath the blots denote the presence (+) or absence (−) of each experimental variable, including glucose level (5.5, 25, or 50 mM), and EAPO concentration. Graphical data are presented as fold changes in protein expression and expressed as mean ± standard deviation (n = 3). CC, compound C; EAPO, ethyl acetate fraction of *Platycladus orientalis*; mTOR, mammalian target of rapamycin.

**FIGURE 12:**
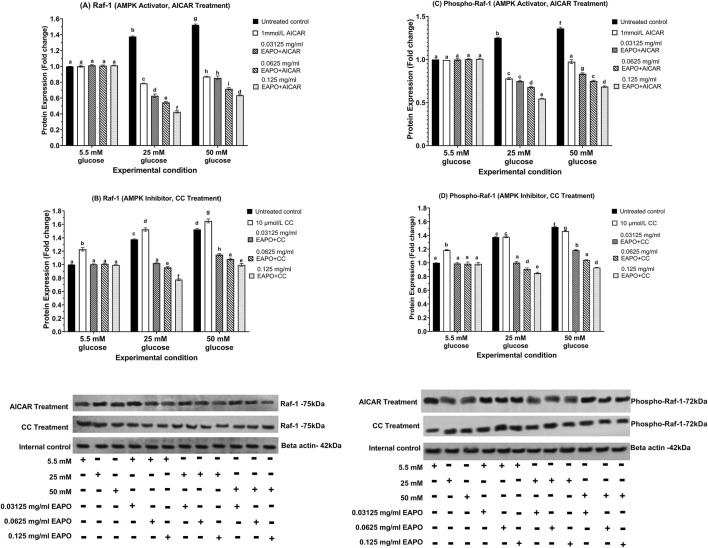
Representative Western blots showing rMc-1 expression of **(A)** Raf-1 in cells treated with EAPO in combination with the AMPK activator, AICAR, **(B)** Raf-1 in cells treated with EAPO in combination with the AMPK inhibitor, Compound C, **(C)** phospho–Raf-1 in cells treated with EAPO and AICAR, and **(D)** phospho–Raf-1 in cells treated with EAPO and Compound C under 5.5 mM, 25 mM, and 50 mM glucose for 48 h. Untreated control refers to rMc-1 cultured under the indicated glucose concentration without EAPO or AMPK modulators (AICAR or CC). The (+/−) symbols beneath the blots denote the presence (+) or absence (−) of each experimental variable, including glucose level (5.5, 25, or 50 mM), and EAPO concentration. Graphical data are presented as fold changes in protein expression and expressed as mean ± standard deviation (n = 3). CC, compound C; EAPO, ethyl acetate fraction of *Platycladus orientalis*; Raf-1, rapidly accelerated fibrosarcoma-1.

**FIGURE 13:**
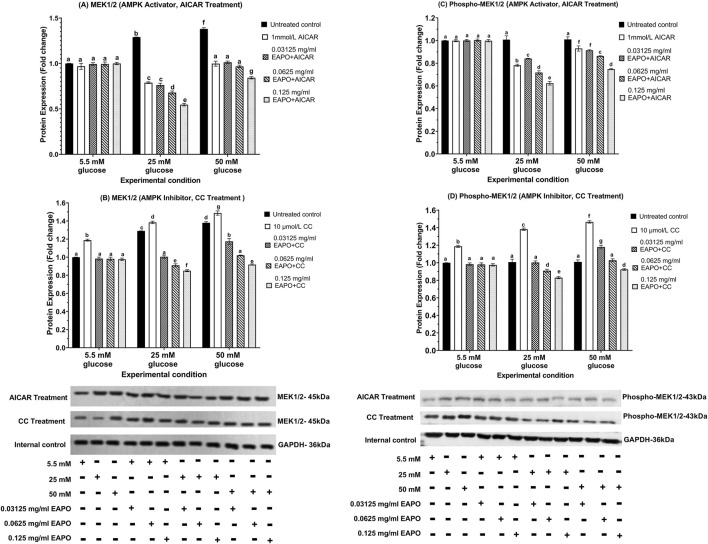
Representative Western blots showing rMc-1 expression of **(A)** MEK1/2 in cells treated with EAPO in combination with the AMPK activator, AICAR, **(B)** MEK1/2 in cells treated with EAPO in combination with the AMPK inhibitor, Compound C, **(C)** phospho-MEK1/2 in cells treated with EAPO and AICAR, and **(D)** phospho-MEK1/2 in cells treated with EAPO and Compound C under 5.5 mM, 25 mM, and 50 mM glucose for 48 h. Untreated control refers to rMc-1 cultured under the indicated glucose concentration without EAPO or AMPK modulators (AICAR or CC). The (+/−) symbols beneath the blots denote the presence (+) or absence (−) of each experimental variable, including glucose level (5.5, 25, or 50 mM), and EAPO concentration. Graphical data are presented as fold changes in protein expression and expressed as mean ± standard deviation (n = 3). CC, compound C; EAPO, ethyl acetate fraction of *Platycladus orientalis*; MEK, mitogen-activated extracellular kinase.

To investigate the role of AMPK in rMc-1 under glucose stress, cells were pretreated with AICAR (AMPK activator) or compound C (AMPK inhibitor), alone or in combination with EAPO, under normal (5.5 mM), high (25 mM), and severely high (50 mM) glucose conditions (Figures 8–13). AICAR consistently suppressed VEGF, VEGFR2, mTOR, Raf-1, and MEK1/2 protein expression and phosphorylation, while enhancing AKT1 and phospho-AKT1 across all glucose concentrations. Importantly, no significant changes were observed under normoglycemia. In contrast, compound C exerted the opposite effect, elevating VEGF, VEGFR2, mTOR, Raf-1, and MEK1/2 expression and phosphorylation, while further reducing AKT1 and phospho-AKT1 even under normal glucose.

At 25 mM glucose, AICAR reduced VEGF, VEGFR2, and phospho-VEGFR2 close to baseline, with further suppression at 50 mM glucose (1.13–1.09-fold) relative to untreated 50 mM control. Conversely, compound C elevated these markers to >1.45-fold under 25 mM glucose and >1.5-fold under 50 mM glucose (Figures 8B, 9B,D). Co-treatment with AICAR and 0.125 mg/mL EAPO further enhanced VEGF/VEGFR2 suppression, achieving reductions of >20% beyond that of EAPO alone at 25 mM glucose, and additional 7%–10% reductions at 50 mM glucose. Notably, phospho-VEGFR2 reached 0.54-fold (25 mM glucose) and 0.67-fold (50 mM glucose) with co-treatment when compared with 0.66- and 0.91-fold for EAPO alone. Under compound C effect, EAPO still reduced VEGF/VEGFR2 expression in a dose-dependent manner, though higher concentrations were required for phospho-VEGFR2 suppression (Figures 8A,B, 9A–D).

For AKT1 signaling, AICAR restored AKT1 and phospho-AKT1 to 5.5 mM control levels for all high glucose conditions (Figures 10A,C). Compound C further suppressed these proteins to ≤0.75-fold at 25 mM glucose and ≤0.68-fold at 50 mM glucose. Remarkably, co-treatment of AICAR with the lowest EAPO dose (0.03125 mg/mL) fully restored AKT1 and phospho-AKT1, whereas EAPO alone required 0.125 mg/mL. By contrast, compound C abolished the restorative effect of low-dose EAPO at 50 mM glucose, though high-dose EAPO (0.125 mg/mL) partially counteracted this inhibition under 25 mM glucose (Figures 10A–D). AICAR also significantly suppressed mTOR, Raf-1, and MEK1/2 and their phosphorylated forms under glucose stress (Figures 11A, 12A, 13A). At 25 mM glucose, these proteins were reduced below 0.8-fold, while at 50 mM glucose, they were restored close to 5.5 mM control levels. Co-treatment with AICAR and 0.125 mg/mL EAPO produced stronger inhibition, reducing mTOR and Raf-1 to <0.45-fold (25 mM glucose) and <0.65-fold (50 mM glucose), with corresponding suppression of phospho-mTOR and phospho-Raf-1 to <0.70-fold (Figures 11A,B, 12A,B). MEK1/2 and phospho-MEK1/2 were also reduced in a dose-dependent manner, with 0.03125 mg/mL EAPO and AICAR sufficient to restore baseline levels at 50 mM glucose, and higher doses providing additional suppression (Figures 13A,C).

Conversely, compound C markedly elevated mTOR, Raf-1, and MEK1/2 expression under 5.5 mM normal glucose (≥1.14-fold), with further increased under high glucose stress, peaking at 1.38–1.65-fold (Figures 11B, 12B, 13B). Their phosphorylated counterparts were similarly upregulated (≥1.36-fold). However, EAPO co-treatment counteracted these effects in a concentration-dependent manner. For instance, under compound C effect at 25 mM glucose, Raf-1 and MEK1/2 were progressively reduced by 33%–48% across increasing EAPO doses, while phospho-Raf-1 and phospho-MEK1/2 required higher EAPO concentrations for baseline restoration at 50 mM glucose (Figures 12B,D, 13B,D) Collectively, these findings demonstrated that AMPK activation suppressed, whereas AMPK inhibition amplified, glucose-induced activation of VEGF and MEK/ERK–mTOR pathways in rMc-1. EAPO enhanced the protective effects of AICAR and mitigates the detrimental effects of compound C, with efficacy dependent on both glucose concentration and EAPO dose (Figures 8–13).

## 4 Discussion

*P. orientalis* is a traditional Chinese medicinal plant historically used to enhance cognitive function and delay aging (Xie et al., 2025). Past pharmacological investigations have demonstrated its therapeutic potential in neurodegenerative disorder such as Alzheimer’s disease, as well as in ocular pathologies (Xu et al., 2022). In this study, the phytochemical profile of EAPO was consistent with previous reports by Khammassi et al. (2022) and Abdallah and Elsharkawy (2019), although comparatively lower levels of diterpenoids and diterpenes were detected, reflecting the differences in plant parts and extraction procedures. Among the five identified phytochemical categories, three diterpenoids, namely, the ferruginol, isosteviol methyl ester, and thunbergol, have been previously linked to antidiabetic, neuroprotective, and anti-inflammatory activities (Khammassi et al., 2022). In addition, flavonoids such as quercitrin, afzelin, amentoflavone, and hinokiflavone are well recognized for their anti-inflammatory and anti-glycation effects, with amentoflavone specifically shown to suppress VEGF expression (Shan et al., 2018). Several non-polar constituents, including α-humulene, α-pinene, sabinene, limonene, α-terpinolene, and α-terpinyl acetate, have also been reported to exert anti-inflammatory and anti-angiogenic actions (Lim et al., 2025; Seo et al., 2019). Taken together, these bioactive compounds likely act in concert to support the therapeutic potential of *P. orientalis*, particularly in mitigating diabetes-induced cytotoxicity and neurodegeneration.

rMc-1 are the major glial cells of the vertebrate retina, responsible for maintaining homeostatic and providing essential metabolic support to neurons (Mecchia et al., 2022). A central component of neurovascular unit, rMc-1 mediate the interactions between neurons and blood vessels, thereby preserving structural function integrity of the retina (Xu et al., 2022). As illustrated in Figure 1, rMc-1 exhibited a radial, elongated morphology with small, indistinct nuclei, characteristics that are essential for maintaining neuronal stability and physiological function (Gad et al., 2024). During the progression of DR, the supportive functions of rMc-1 are markedly impaired. Glucose stress is a well-established inducer of rMc-1 activation, a defining feature of reactive retinal gliosis (Xu et al., 2022). Exposure to high glucose concentrations (25 and 50 mM) induced pronounced morphological changes, including loss of the typical elongated shape, cell body hypertrophy, irregular borders, and retraction of cellular processes. These alterations, together with reduced cell viability, may be partly attributed to osmotic stress caused by elevated extracellular glucose, and are often accompanied by apoptotic degeneration, indicating a diminished capacity of rMc-1 cells to tolerate and adapt to glucose-induced cytotoxicity (Gad et al., 2024). In addition, elevated glucose impairs rMc-1 viability and weakens their antioxidant defenses, collectively promoting neuronal dysfunction and accelerating neurodegenerative processes (Güngör Kobat, 2020).

The IC_50_ of EAPO in rMc-1 was determined to be 0.323 mg/mL. For comparison, plant fractions with an IC_50_ below 0.1 mg/mL are generally classified as cytotoxic to normal cells (Mbugua et al., 2022). The MTT assay demonstrated that EAPO at 0.125 mg/mL or higher significantly reduced rMc-1 viability to below 70%. At concentrations above 0.5 mg/mL, rMc-1’s viability dropped below 50%, indicating that the IC_50_ threshold had been exceeded. Exposure to EAPO concentrations above 0.125 mg/mL induced hypertonic stress in rMc-1, manifested as a slight reduction in cytoplasmic volume due to osmotic imbalance. Such hyperosmolar conditions may disrupt ion homeostasis and compromise cellular integrity, thereby contributing to stress responses (Zayani et al., 2025). These findings suggest that at higher concentrations, EAPO may exert cytotoxic effects not only through osmotic stress but also by potentially inducing cell cycle arrest. In the early stages of DR, glucose-induced stress impairs rMc-1 glutamate uptake, leading to gliosis and ultimately contributing to retinal neurodegeneration (Balzamino et al., 2024). The present study showed a significant reduction in rMc-1 viability under high glucose stress when compared to the normal glucose control group (Figure 2). However, treatment with EAPO restored the rMc-1 viability back to >80% under glucose stress. At 0.125 mg/mL, EAPO restored rMc-1 viability to nearly 90% under high-glucose conditions (25 and 50 mM), suggesting that this EAPO concentration exerts a maximal protective effect. The observed neuroprotection was likely mediated by the major bioactive constituents, particularly ferruginol, thunbergol, and isosteviol methyl ester (Table 1).

Glucose stress–induced cytotoxicity and neurodegeneration drive rMc-1 gliosis, a key event in the progression of DR (Mecchia et al., 2022). Upon activation, rMc-1 markedly upregulated VEGF and its receptor VEGFR2, representing a canonical response to neurodegenerative insults. Enhanced VEGF/VEGFR2 signaling subsequently activates the downstream AKT1/mTOR and Raf-1/MEK1/2 pathways, thereby promoting neuronal apoptosis and degeneration (Yang et al., 2022). In the present study, high-glucose exposure significantly upregulated both gene and protein expression of VEGF and VEGFR2 in rMc-1, consistent with previous reports by Vellanki et al. (2016) and Mecchia et al. (2022), whom demonstrated that VEGF/VEGFR2 overactivation disrupted neurotrophin production and compromised neuronal integrity. Mechanistically, VEGFR2 contains two critical autophosphorylation sites (Tyr1054 and Tyr1059) within its kinase domain, whose phosphorylation upon VEGF binding initiates VEGFR2 signaling cascades (Wang et al., 2020). This activation suppresses the production of neurotrophic factors such as brain-derived neurotrophic factor and glial cell-derived neurotrophic factor (Guo et al., 2024). Prolonged VEGF–VEGFR2 activation further impairs glutamate receptor function, particularly N-methyl-D-aspartate receptor–mediated uptake, resulting in glutamate accumulation (Le, 2017; Shah et al., 2025).

Sustained VEGF–VEGFR2 activation under glucose stress upregulated downstream AKT1/mTOR signaling, thereby promoting neurodegeneration (Wang et al., 2020). In this study, persistent glucose stress markedly suppressed AKT1 expression and reduced phospho-AKT1 levels in both cytoplasmic fractions and whole-cell lysates (Figures 6A,B), consistent with earlier findings (Rai et al., 2019; Hassan et al., 2024). This impairment disrupted AKT1-mediated survival signaling, including suppression of insulin-like growth factor I (IGF-I), and facilitated glycogen synthase kinase-3 (GSK-3) activation, ultimately driving neuronal apoptosis (Li X. et al., 2023). These findings demonstrated that glucose-induced inhibition of AKT1 signaling compromised essential pro-survival pathways, accelerating early neurodegenerative events in DR (Hassan et al., 2024; Rai et al., 2019). On the other hand, mTOR is localized to multiple subcellular compartments, including the plasma membrane, cytosol, and nucleus where it exerted context-dependent regulatory functions (Giguère, 2018). In the current study, glucose stress induced robust upregulation of mTOR across all three compartments in rMc-1 (Figure 6C). As a master regulator of protein synthesis and cellular metabolism, mTOR displays maladaptive activation under hyperglycemic stress (Panwar et al., 2023). In the cytoplasmic and whole-cell fractions, increased mTOR activation (Figure 6C) was associated with enhanced protein synthesis, exacerbating cellular stress and organelle dysfunction (Marafie et al., 2024). The nuclear accumulation of mTOR (Figure 6C) suggested transcriptional reprogramming, which was characterized by the downregulation of neuroprotective factors and stress-response genes (Zhao et al., 2024). Notably, the pathological impact was strongly dependent on phosphorylation at Ser2448 (phospho-mTOR), which was markedly elevated under glucose stress (Figure 6D). Persistent phospho-mTOR activation impaired clearance of damaged mitochondria and misfolded proteins while amplifying pro-apoptotic signaling (Panwar et al., 2023), thereby aggravating rMc-1 dysfunction.

Another key signaling cascade implicated in neurodegeneration is the Raf-1/MEK1/2 pathway, which functions downstream of VEGF–VEGFR2 activation (Bahar et al., 2023). In this study, glucose stress significantly upregulated Raf-1 expression, with more pronounced activation at the plasma membrane when compared to the cytoplasm (Figure 7A), consistent with its well-established membrane-associated regulation (Bahar et al., 2023). Phosphorylation of Raf-1 at Tyr340/341 facilitated its activation, which in turn phosphorylated the MEK1/2, as reflected by the increased levels of phospho-MEK1/2 at Ser218/222 (Gravandi et al., 2023). This sequential Raf-1/MEK1/2 signaling cascade drives MAPK activation, thereby amplifying neuroinflammatory responses through Müller cell gliosis and increased release of pro-inflammatory cytokines (Shin et al., 2024). Sustained activation of this pathway perpetuates glial reactivity and accelerates retinal neurodegeneration. Collectively, these findings highlight MEK1/2 as critical downstream effectors of Raf-1 in mediating glucose stress–induced neurodegenerative signaling in rMc-1.

EAPO demonstrated strong neuroprotective potential by attenuating VEGF and VEGFR2 expression in rMc-1 under glucose stress. In addition to reducing protein expression, EAPO suppressed VEGFR2 autophosphorylation at Tyr1054 and Tyr1059, thereby inhibiting VEGF/VEGFR2 signaling (Figures 5A,B). This suppression was likely to mitigate extracellular glutamate accumulation and receptor overactivation, ultimately protecting against neuronal apoptosis (Wu et al., 2025). According to Varbanov et al. (2023) and Farzeen et al. (2026**)**, ferruginol, a diterpenoid identified in EAPO, has been reported to exert neuroprotective effects under hyperglycemic conditions by modulating VEGF expression via the stabilization of hypoxia-inducible factor-1 alpha and regulation of MAPK cascade (Varbanov et al., 2023; Farzeen et al., 2026). This is particularly relevant to the current findings, as AMPK activation is known to negatively regulate hypoxia-inducible factor-1 alpha and downstream VEGF signaling, suggesting that ferruginol may contribute to the observed suppression of VEGF-related pathways through AMPK-dependent mechanisms (Farzeen et al., 2026). Notably, at 0.03125 mg/mL, EAPO reactivated AKT1 in the whole-cell lysates and cytoplasmic fractions, while restoring its phosphorylated form (phospho-AKT1 at Ser473) in the cytoplasm and nucleus (Figures 6A,B). This restoration may enhance neuronal survival by supporting IGF-I–mediated pro-survival signaling and inhibiting GSK-3–driven apoptotic pathways (Li J. et al., 2023). EAPO also exerted a strong inhibitory effect on mTOR and its phosphorylated form (p-mTOR) under glucose stress (Figures 6C,D). Cytoplasmic mTOR was particularly sensitive to EAPO-mediated suppression when compared to the nuclear and whole-cell compartments (Figure 6C), suggesting that EAPO preferentially disrupts cytoplasmic mTOR signaling, thereby reducing maladaptive processes such as dysregulated protein synthesis (Torres and Holz, 2021). In contrast, higher EAPO concentrations were required to suppress nuclear mTOR and its regulatory complexes (Figure 6C), potentially contributing to neuroprotection through enhanced clearance of aggregated proteins (Amin et al., 2024).

Both EAPO treatment and AMPK activation resulted in the suppression of the Raf-1/MEK pathway, a key mediator of glucose-induced neuroinflammation. EAPO significantly reduced Raf-1 expression and phosphorylation at Tyr340/341, along with downstream MEK1/2 and phospho-MEK1/2 (Ser218/222) (Figures 12, 13). The dose-dependent effects observed in both whole-cell and cytoplasmic compartments likely attenuated ERK activation, thereby suppressing Müller cell activation, gliotic marker expression, and pro-inflammatory cytokine release (Gravandi et al., 2023; Shin et al., 2024). Similarly, AMPK activation by AICAR increased the AMP/ATP ratio under high-glucose conditions, leading to inhibition of VEGF–VEGFR2, mTOR, and Raf-1/MEK1/2 signaling (Marín-Aguilar et al., 2017). This effect was abolished by the AMPK inhibitor compound C, confirming the regulatory role of AMPK (Figures 8–13). Importantly, AMPK activation enhanced AKT1 phosphorylation at Ser473, restoring IGF-1–mediated pro-survival signaling while suppressing GSK-3–driven apoptosis (Li J. et al., 2023). In parallel, AMPK inhibited mTOR and phospho-mTOR, reducing protein synthesis while promoting autophagy to clear damaged organelles and aggregated proteins (Višnjić et al., 2021). Collectively, these findings showed that EAPO and AMPK activation act through complementary mechanisms to disrupt the Raf-1/MEK axis and attenuate neuroinflammation, thereby protecting Müller cells from glucose-induced cytotoxicity and degeneration (Saito et al., 2019). Ullah et al. (2019) previously demonstrated that isosteviol methyl ester suppressed ERK activity by inhibiting NF-κB activation and reducing pro-inflammatory cytokine production. Considering that AMPK activation is closely linked to anti-inflammatory responses and can suppress NF-κB signaling, the isosteviol derivatives in EAPO may synergistically enhance AMPK-mediated neuroprotection by mitigating ERK/NF-κB-driven neuroinflammatory pathways. Combined EAPO and AICAR treatment synergistically suppressed VEGF/VEGFR2 expression (Figures 8A, 9A), indicating cooperative reinforcement of AMPK signaling to counter glucose-induced cytotoxicity and neurodegeneration (Marín-Aguilar et al., 2017). This synergistic activation promoted AKT1 phosphorylation at Ser473, restored IGF-1–dependent survival pathways, and enhanced mTOR-dependent autophagic clearance of damaged mitochondria and protein aggregates (Chadha and Meador-Woodruff, 2020; Li J. et al., 2023). Concomitantly, the combined treatments have been demonstrated to attenuate mTOR overactivation and suppressed Raf-1/MEK1/2–mediated neuroinflammation, thereby preserving Müller cell viability (Shin et al., 2024). In contrast, AMPK inhibition with compound C abolished these protective effects, leading to elevated VEGF/VEGFR2 expression (Figures 8B, 9B), persistent mTOR activation (Figure 10B), impaired autophagy, glutamate accumulation, and enhanced Raf-1/MEK1/2 signaling, which collectively exacerbated oxidative stress, apoptosis, and gliosis (Wu and Zou, 2020; Torres and Holz, 2021; Shukal et al., 2022). Notably, co-treatment with EAPO partially mitigated these effects only at higher concentrations (Figures 8–13) underscoring its reliance on intact AMPK signaling. Together, these results identify AMPK as a central mediator of EAPO’s protective actions, positioning EAPO as a natural AMPK potentiator with therapeutic potential for targeting Müller cell dysfunction and hyperglycemia-induced neurodegeneration in DR.

## 5 Conclusion

This study demonstrates that the ethyl acetate fraction of *P. orientalis* (EAPO; IC_50_ = 0.323 mg/mL) restores rMc-1 viability under high-glucose conditions and attenuates neurodegenerative responses in a dose-dependent manner. Mechanistically, EAPO enhances AMPK activation and modulates VEGF/VEGFR2, AKT1/mTOR, and Raf-1/MEK1/2 signaling, thereby reducing neuronal damage and apoptosis. These effects are likely attributed to its bioactive constituents, including monoterpene esters, diterpenoids, fatty acids, phenolic glycosides, and chlorophyll derivatives. Collectively, these findings support *P. orientalis* as a potential natural AMPK activator and provide insight into AMPK-mediated neuroprotection in diabetic retinopathy. Nevertheless, the inclusion of osmotic controls such as mannitol would strengthen the current study, as high glucose may induce osmotic stress independent of metabolic effects. Given that the present work is limited to *in vitro* data, *in vivo* studies are required to validate its therapeutic potential. Future investigations ought to incorporate computational approaches, such as molecular docking, to evaluate interactions between key compounds (ferruginol, thunbergol, and isosteviol methyl ester) and relevant targets, including VEGF/VEGFR2, Raf-1/MEK, and AKT1/mTOR pathways.

## Data Availability

The datasets presented in this study can be found in online repositories. The names of the repository/repositories and accession number(s) can be found in the article/Supplementary Material.
