# Supplementary material for: Neuroprotective effects of Platycladus orientalis ethyl acetate fraction on retinal Müller cells: modulation of AKT1/mTOR and Raf-1/MEK1/2 pathways via AMPK activation
Source: Front Mol Biosci. 2026 May 22;13:1802106. doi: 10.3389/fmolb.2026.1802106 (PMC13236534; doi:10.3389/fmolb.2026.1802106)
Supplement: Supplementary file 1 [file DataSheet1.docx]

***Supplementary Material***

**Supplement 1.** List of chemicals used in the study

| **Chemicals** | **Brands** | **Manufacturing country** |
| --- | --- | --- |
| 5-aminoimidazole-4-carboxamide riboside (AICAR) | Sigma-Aldrich | Missouri, U.S.A |
| 3- (4,5-dimethylthiazol-2-yl)-2,5-diphenyltetrazolium bromide (MTT) | Merck Millipore | Massachusetts, U.S.A. |
| 4X protein loading buffer | Merck Millipore | Massachusetts, U.S.A. |
| Absolute ethanol | American Type Culture Collection | Massachusetts, U.S.A. |
| Acrylamide/bisacrylamide solution (40%, 37.5:1) | Nacalai Tesque | Kyoto, Japan |
| Ammonium persulfate | Sigma-Aldrich | Missouri, U.S.A. |
| Bovine Serum Albumin (BSA) | Sigma-Aldrich | Missouri, U.S.A |
| Chemiluminescent substrate | Nacalai Tesque | Kyoto, Japan |
| Chloroform | R & M Chemicals | London, U.K |
| D-glucose | Merck Millipore | Massachusetts, U.S.A. |
| Dimethyl sulfoxide (DMSO) | Merck Millipore | Massachusetts, U.S.A. |
| DNA ladder | Nacalai Tesque | Kyoto, Japan |
| Dorsomorphin (Compound C) | Sigma-Aldrich | Missouri, U.S.A |
| Ethyl acetate | R & M Chemicals | London, U.K |
| Glacial acetic acid | R & M Chemicals | London, U.K |
| Hexane | R & M Chemicals | London, U.K. |
| High-capacity cDNA reverse transcription kit | Thermo Fisher Scientific | Massachusetts, U.S.A. |
| HRP conjugated secondary antibody | LI-COR Biosciences | Nebraska, USA |
| Hydrochloric acid (HCl) | R & M Chemicals | London, U.K |
| Methanol | Merck Millipore | Massachusetts, U.S.A. |
| N,N,N,N-tetramethylethylenediamine | Sigma-Aldrich | Missouri, U.S.A. |
| n-butanol | R & M Chemicals | London, U.K |
| Non-fat dry milk | Nacalai Tesque | Kyoto, Japan |
| Nuclear and cytoplasmic extraction kit | Thermo Fisher Scientific | Massachusetts, U.S.A. |
| Penicillin-streptomycin | Nacalai Tesque | Kyoto, Japan |
| Phosphatase inhibitor cocktail | Nacalai Tesque | Kyoto, Japan |
| Pierce BCA protein assay kit | Thermo Fisher Scientific | Massachusetts, U.S.A. |
| Potassium acetate | R & M Chemicals | London, U.K |
| Primary antibodies | Thermo Fisher Scientific | Massachusetts, U.S.A. |
| Protein ladder | Nacalai Tesque | Kyoto, Japan |
| PVDF membranes | Merck Millipore | Massachusetts, U.S.A. |
| Retinal rat Muller cell | Kerafast | Boston, U.S.A |
| Ribonuclease-free deoxyribonuclease | R & M Chemicals | London, U.K |
| RIPA lysis buffer | Thermo Fisher Scientific | Massachusetts, U.S.A. |
| SensiFAST™ SYBR® qPCR kit | Meridian Bioscience | Ohio, USA |
| Sodium chloride | Nacalai Tesque | Kyoto, Japan |
| Sodium dodecyl sulfate (SDS) | Nacalai Tesque | Kyoto, Japan |
| Sodium phosphate monobasic | Sigma-Aldrich | Missouri, U.S.A |
| Tris(hydroxymethyl)aminomethane | Sigma-Aldrich | Missouri, U.S.A. |
| Trizol reagent | St. John’s Laboratory | London, United Kingdom |
| Trypsin-EDTA | Sigma-Aldrich | Missouri, U.S.A |
| Tween® 20 | Nacalai Tesque | Kyoto, Japan |

**Supplement 2.** The forward and reverse primers sequences for selected target genes

| **Gene** | **Forward primer (5’-3’)** | **Reverse primer (5’-3’)** |
| --- | --- | --- |
| β-actin  (housekeeping) | AGC CAT GTA CGT AGC CAT CC | ACC CTC ATA GAT GGG CAC AG |
| AKT-1 | GAT CCT GGT GAA GGA GAA GG | TTC TCG GAG TGC AAG TAG TC |
| mTOR | TTA TGC CAA CCT CCT AGC TG | GAG GTA ACA GGA TGG TGG AG |
| MEK 1 | CAT TGT CTC ACT GTG TTG CC | GGA GCT ACT GAA GGC TAG TG |
| MEK 2 | CCT CCA ACA TTC TGG TGA AC | AGC GTG TCC CTA CAA ATG AG |
| Raf-1 | CAA GTG GCA TGG AGA TGT TG | ATT TGG TCT CCT GGA CAT GC |
| VEGF | GAC TAT TCA GCG GAC TCA CC | CCG TTG GCA CGA TTT AAG AG |
| VEGFR2 | TAG CAC GAC AGA GAC TGT GAG G | TGA GGT GAG AGA GAT GGG TAG G |

**Supplement 3.** The primary and secondary antibodies for western blot

| **Catalogue number** | **Antibody** | **Titre** | **Volume (μl )** | **Centrifugation (rpm)** | **Source** |
| --- | --- | --- | --- | --- | --- |
| 710005 | AKT1 | 1: 500 | 50 | 3000 | Thermo Fisher Scientific, Massachusetts, USA |
| STJ91464 | β-actin | 1:10000 | 1 |  | St John’s Laboratory, U.K. |
| FNab03342 | GAPDH | 1: 5000 | 5 |  | Fine test, USA |
| GTX213110 | Horseradish peroxidase-conjugated anti-rabbit secondary antibodies | 1:1000 | 10 |  | GeneTex, U.S. |
| PA5-31917 | MEK1/2 | 1:1000 | 10 |  | Thermo Fisher Scientific, Massachusetts, USA |
| FNab05417 | mTOR | 1: 300 | 30 |  | Fine test, USA |
| 700392 | Phospho-AKT1 | 1: 1000 | 10 |  | Thermo Fisher Scientific, Massachusetts, USA |
| 44-506G | Phospho- Raf-1 | 1:1000 | 10 |  | Thermo Fisher Scientific, Massachusetts, USA |
| 44-454G | Phospho-MEK1/2 | 1:1000 | 10 |  | Thermo Fisher Scientific, Massachusetts, USA |
| 44-1125G | Phospho-mTOR | 1:1000 | 10 |  | Thermo Fisher Scientific, Massachusetts, USA |
| 44-1047G | Phospho-VEGFR2 | 1:1000 | 10 |  | Thermo Fisher Scientific, Massachusetts, USA |
| FNab07087 | Raf-1 | 1:1000 | 10 |  | Fine test, USA |
| STJ114190 | VEGF | 1:1000 | 10 |  | St John’s Laboratory, London, UK |
| MA5-15157 | VEGFR2 | 1:1000 | 10 |  | Thermo Fisher Scientific, Massachusetts, USA |


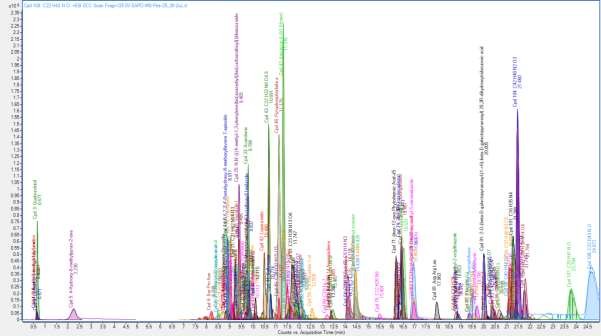


**Supplementary 4.** The chromatogram for the positive ionization of *P. orientalis* ethyl acetate fraction


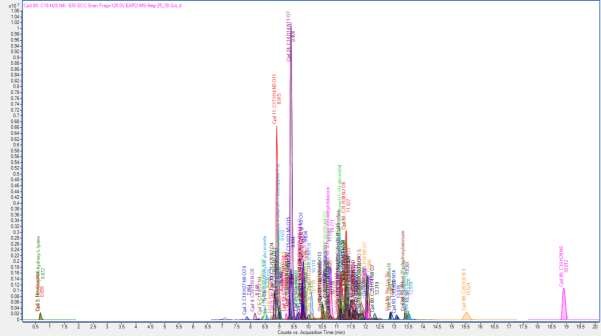


**Supplementary 5.** The chromatogram for the negative ionization of *P. orientalis* ethyl acetate fraction.


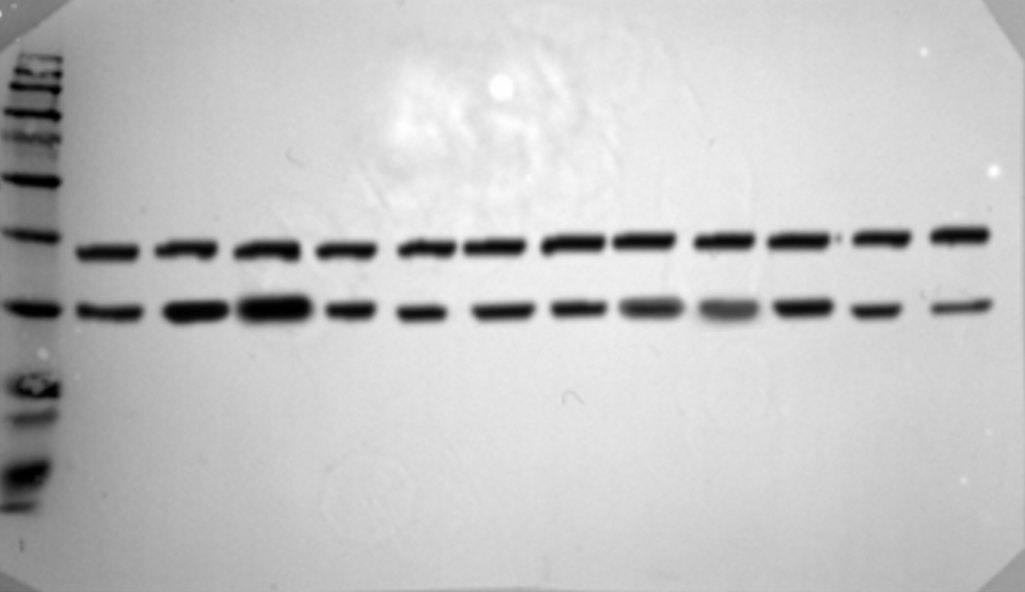
Whole cell lysate

**1 2 3 4 5 6 7 8 9 10 11 12 13**

**Lane 1: ladder**

**Lane 2 5.5mM glucose**

**Lane 3: 25mM glucose**

**Lane 4: 50mM glucose**

**Lane 5: 5.5 mM with 0.03125mg/ml ethyl acetate *P. orientalis* (EAPO)**

**Lane 6: 5.5 mM with 0.0625mg/ml ethyl acetate *P. orientalis* (EAPO)**

**Lane 7: 5.5 mM with 0.125mg/ml ethyl acetate *P. orientalis* (EAPO)**

**Lane 8: 25 mM with 0.03125mg/ml ethyl acetate *P. orientalis* (EAPO)**

**Lane 9: 25 mM with 0.0625mg/ml ethyl acetate *P. orientalis* (EAPO)**

**Lane 10: 25 mM with 0.125mg/ml ethyl acetate *P. orientalis* (EAPO)**

**Lane 11: 50 mM with 0.03125mg/ml ethyl acetate *P. orientalis* (EAPO)**

**Lane 12 : 50 mM with 0.0625mg/ml ethyl acetate *P. orientalis* (EAPO)**

**Lane 13 : 50 mM with 0.125mg/ml ethyl acetate *P. orientalis* (EAPO)**

**245**

**180**

**140**

**100**

**75**

**60**

**45**

**35**

**25**

**20**

**15**

**10**

**5**

Beta actin -42kDa

VEGF-35kDa

Cytoplasmic fraction

**1 2 3 4 5 6 7 8 9 10 11 12 13**

**Lane 1: ladder**

**Lane 2 5.5mM glucose**

**Lane 3: 25mM glucose**

**Lane 4: 50mM glucose**

**Lane 5: 5.5 mM with 0.03125mg/ml ethyl acetate *P. orientalis* (EAPO)**

**Lane 6: 5.5 mM with 0.0625mg/ml ethyl acetate *P. orientalis* (EAPO)**

**Lane 7: 5.5 mM with 0.125mg/ml ethyl acetate *P. orientalis* (EAPO)**

**Lane 8: 25 mM with 0.03125mg/ml ethyl acetate *P. orientalis* (EAPO)**

**Lane 9: 25 mM with 0.0625mg/ml ethyl acetate *P. orientalis* (EAPO)**

**Lane 10: 25 mM with 0.125mg/ml ethyl acetate *P. orientalis* (EAPO)**

**Lane 11: 50 mM with 0.03125mg/ml ethyl acetate *P. orientalis* (EAPO)**

**Lane 12 : 50 mM with 0.0625mg/ml ethyl acetate *P. orientalis* (EAPO)**

**Lane 13 : 50 mM with 0.125mg/ml ethyl acetate *P. orientalis* (EAPO)**

**245**

**180**

**140**

**100**

**75**

**60**

**45**

**35**

**25**

**20**

**15**

**10**

**5**


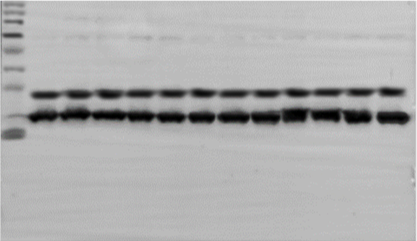


VEGF-35kDa

Beta actin -42kDa


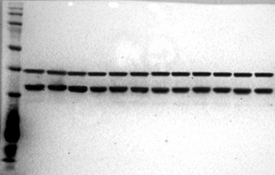
Nuclei fraction

**245**

**180**

**140**

**100**

**75**

**60**

**45**

**35**

**25**

**20**

**15**

**10**

**5**

**1 2 3 4 5 6 7 8 9 10 11 12 13**

**Lane 1: ladder**

**Lane 2 5.5mM glucose**

**Lane 3: 25mM glucose**

**Lane 4: 50mM glucose**

**Lane 5: 5.5 mM with 0.03125mg/ml ethyl acetate *P. orientalis* (EAPO)**

**Lane 6: 5.5 mM with 0.0625mg/ml ethyl acetate *P. orientalis* (EAPO)**

**Lane 7: 5.5 mM with 0.125mg/ml ethyl acetate *P. orientalis* (EAPO)**

**Lane 8: 25 mM with 0.03125mg/ml ethyl acetate *P. orientalis* (EAPO)**

**Lane 9: 25 mM with 0.0625mg/ml ethyl acetate *P. orientalis* (EAPO)**

**Lane 10: 25 mM with 0.125mg/ml ethyl acetate *P. orientalis* (EAPO)**

**Lane 11: 50 mM with 0.03125mg/ml ethyl acetate *P. orientalis* (EAPO)**

**Lane 12 : 50 mM with 0.0625mg/ml ethyl acetate *P. orientalis* (EAPO)**

**Lane 13 : 50 mM with 0.125mg/ml ethyl acetate *P. orientalis* (EAPO)**

Beta actin -42kDa

VEGF-35kDa

**Supplement 6.** Gel blot images for VEGF (35 kDa) and beta-actin (42 kDa) expression in respective whole cell lysate, cytoplasmic, and nuclei fractions of retinal Müller cells. (Corresponds to Figure 5A)

Whole cell lysate

**Lane 1: ladder**

**Lane 2 5.5mM glucose**

**Lane 3: 25mM glucose**

**Lane 4: 50mM glucose**

**Lane 5: 5.5 mM with 0.03125mg/ml ethyl acetate *P. orientalis* (EAPO)**

**Lane 6: 5.5 mM with 0.0625mg/ml ethyl acetate *P. orientalis* (EAPO)**

**Lane 7: 5.5 mM with 0.125mg/ml ethyl acetate *P. orientalis* (EAPO)**

**Lane 8: 25 mM with 0.03125mg/ml ethyl acetate *P. orientalis* (EAPO)**

**Lane 9: 25 mM with 0.0625mg/ml ethyl acetate *P. orientalis* (EAPO)**

**Lane 10: 25 mM with 0.125mg/ml ethyl acetate *P. orientalis* (EAPO)**

**Lane 11: 50 mM with 0.03125mg/ml ethyl acetate *P. orientalis* (EAPO)**

**Lane 12: 50 mM with 0.0625mg/ml ethyl acetate *P. orientalis* (EAPO)**

**Lane 13: 50 mM with 0.125mg/ml ethyl acetate *P. orientalis* (EAPO)**

**1 2 3 4 5 6 7 8 9 10 11 12 13**

**245**

**180**

**140**

**100**

**75**

**60**

**45**

**35**

**25**

**20**

**15**

**10**

**5**


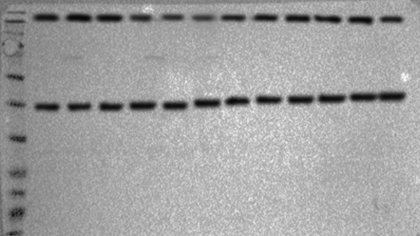


Beta actin -42kDa

VEGFR2-210kDa

Cytoplasmic fraction

**1 2 3 4 5 6 7 8 9 10 11 12 13**

**Lane 1: ladder**

**Lane 2 5.5mM glucose**

**Lane 3: 25mM glucose**

**Lane 4: 50mM glucose**

**Lane 5: 5.5 mM with 0.03125mg/ml ethyl acetate *P. orientalis* (EAPO)**

**Lane 6: 5.5 mM with 0.0625mg/ml ethyl acetate *P. orientalis* (EAPO)**

**Lane 7: 5.5 mM with 0.125mg/ml ethyl acetate *P. orientalis* (EAPO)**

**Lane 8: 25 mM with 0.03125mg/ml ethyl acetate *P. orientalis* (EAPO)**

**Lane 9: 25 mM with 0.0625mg/ml ethyl acetate *P. orientalis* (EAPO)**

**Lane 10: 25 mM with 0.125mg/ml ethyl acetate *P. orientalis* (EAPO)**

**Lane 11: 50 mM with 0.03125mg/ml ethyl acetate *P. orientalis* (EAPO)**

**Lane 12: 50 mM with 0.0625mg/ml ethyl acetate *P. orientalis* (EAPO)**

**Lane 13: 50 mM with 0.125mg/ml ethyl acetate *P. orientalis* (EAPO)**


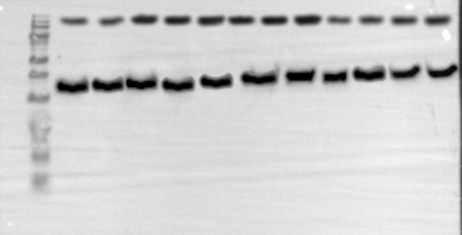


VEGFR2-210kDa

Beta actin -42kDa

**245**

**180**

**140**

**100**

**75**

**60**

**45**

**35**

**25**

**20**

**15**

**10**

**5**

Nuclei fraction

**1 2 3 4 5 6 7 8 9 10 11 12 13**

**Lane 1: ladder**

**Lane 2 5.5mM glucose**

**Lane 3: 25mM glucose**

**Lane 4: 50mM glucose**

**Lane 5: 5.5 mM with 0.03125mg/ml ethyl acetate *P. orientalis* (EAPO)**

**Lane 6: 5.5 mM with 0.0625mg/ml ethyl acetate *P. orientalis* (EAPO)**

**Lane 7: 5.5 mM with 0.125mg/ml ethyl acetate *P. orientalis* (EAPO)**

**Lane 8: 25 mM with 0.03125mg/ml ethyl acetate *P. orientalis* (EAPO)**

**Lane 9: 25 mM with 0.0625mg/ml ethyl acetate *P. orientalis* (EAPO)**

**Lane 10: 25 mM with 0.125mg/ml ethyl acetate *P. orientalis* (EAPO)**

**Lane 11: 50 mM with 0.03125mg/ml ethyl acetate *P. orientalis* (EAPO)**

**Lane 12: 50 mM with 0.0625mg/ml ethyl acetate *P. orientalis* (EAPO)**

**Lane 13: 50 mM with 0.125mg/ml ethyl acetate *P. orientalis* (EAPO)**


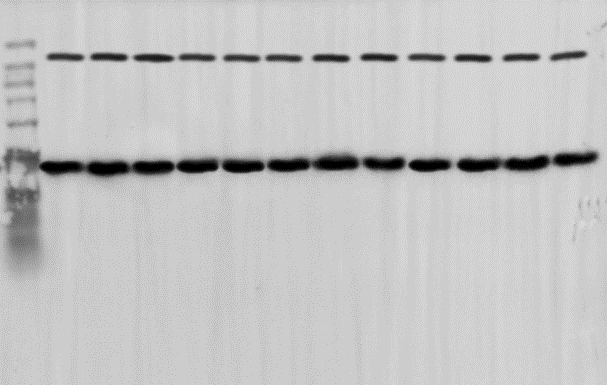


Beta actin -42kDa

VEGFR2-210kDa

**245**

**180**

**140**

**100**

**75**

**60**

**45**

**35**

**25**

**20**

**15**

**10**

**5**

**Supplement 7.** Gel blot images for VEGFR2 (210 kDa) and beta actin (42 kDa) expression in respective whole cell lysate, cytoplasmic, and nuclei fractions of retinal Müller cells. (Corresponds to Figure 5B)

Whole cell lysate

**1 2 3 4 5 6 7 8 9 10 11 12 13**

**Lane 1: ladder**

**Lane 2 5.5mM glucose**

**Lane 3: 25mM glucose**

**Lane 4: 50mM glucose**

**Lane 5: 5.5 mM with 0.03125mg/ml ethyl acetate *P. orientalis* (EAPO)**

**Lane 6: 5.5 mM with 0.0625mg/ml ethyl acetate *P. orientalis* (EAPO)**

**Lane 7: 5.5 mM with 0.125mg/ml ethyl acetate *P. orientalis* (EAPO)**

**Lane 8: 25 mM with 0.03125mg/ml ethyl acetate *P. orientalis* (EAPO)**

**Lane 9: 25 mM with 0.0625mg/ml ethyl acetate *P. orientalis* (EAPO)**

**Lane 10: 25 mM with 0.125mg/ml ethyl acetate *P. orientalis* (EAPO)**

**Lane 11: 50 mM with 0.03125mg/ml ethyl acetate *P. orientalis* (EAPO)**

**Lane 12: 50 mM with 0.0625mg/ml ethyl acetate *P. orientalis* (EAPO)**

**Lane 13: 50 mM with 0.125mg/ml ethyl acetate *P. orientalis* (EAPO)**

**245**

**180**

**140**

**100**

**75**

**60**

**45**

**35**

**25**

**20**

**15**

**10**

**5**


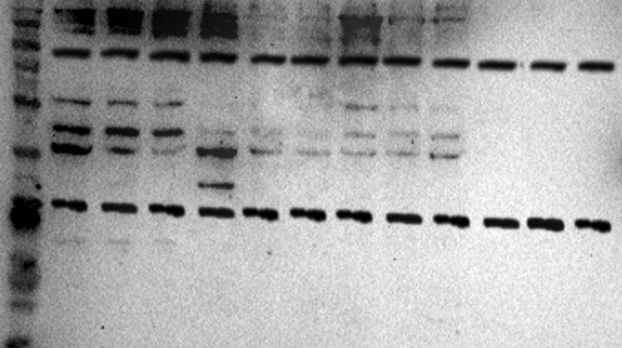


Beta actin -42kDa

Phospho-VEGFR2-130kDa


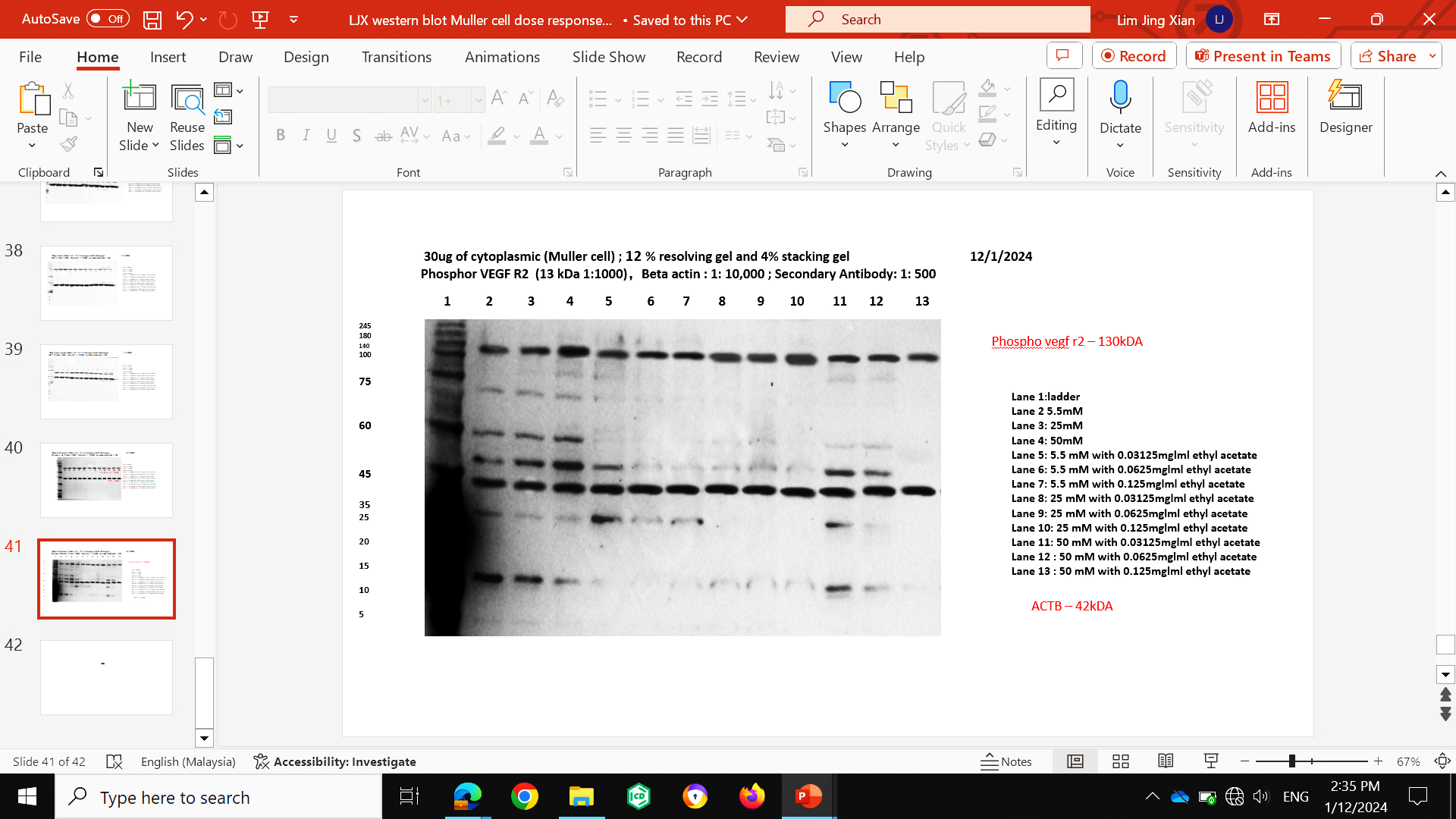
Cytoplasmic fraction

**245**

**180**

**140**

**100**

**75**

**60**

**45**

**35**

**25**

**20**

**15**

**10**

**5**

**1 2 3 4 5 6 7 8 9 10 11 12 13**

Phospho-VEGFR2-130kDa

**Lane 1: ladder**

**Lane 2 5.5mM glucose**

**Lane 3: 25mM glucose**

**Lane 4: 50mM glucose**

**Lane 5: 5.5 mM with 0.03125mg/ml ethyl acetate *P. orientalis* (EAPO)**

**Lane 6: 5.5 mM with 0.0625mg/ml ethyl acetate *P. orientalis* (EAPO)**

**Lane 7: 5.5 mM with 0.125mg/ml ethyl acetate *P. orientalis* (EAPO)**

**Lane 8: 25 mM with 0.03125mg/ml ethyl acetate *P. orientalis* (EAPO)**

**Lane 9: 25 mM with 0.0625mg/ml ethyl acetate *P. orientalis* (EAPO)**

**Lane 10: 25 mM with 0.125mg/ml ethyl acetate *P. orientalis* (EAPO)**

**Lane 11: 50 mM with 0.03125mg/ml ethyl acetate *P. orientalis* (EAPO)**

**Lane 12: 50 mM with 0.0625mg/ml ethyl acetate *P. orientalis* (EAPO)**

**Lane 13: 50 mM with 0.125mg/ml ethyl acetate *P. orientalis* (EAPO)**

Beta actin -42kDa


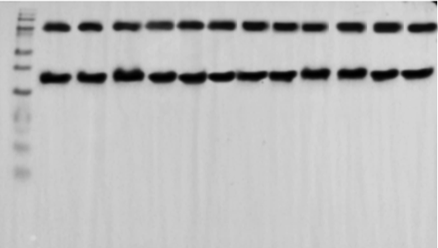
Nuclei fraction

**1 2 3 4 5 6 7 8 9 10 11 12 13**

**Lane 1: ladder**

**Lane 2 5.5mM glucose**

**Lane 3: 25mM glucose**

**Lane 4: 50mM glucose**

**Lane 5: 5.5 mM with 0.03125mg/ml ethyl acetate *P. orientalis* (EAPO)**

**Lane 6: 5.5 mM with 0.0625mg/ml ethyl acetate *P. orientalis* (EAPO)**

**Lane 7: 5.5 mM with 0.125mg/ml ethyl acetate *P. orientalis* (EAPO)**

**Lane 8: 25 mM with 0.03125mg/ml ethyl acetate *P. orientalis* (EAPO)**

**Lane 9: 25 mM with 0.0625mg/ml ethyl acetate *P. orientalis* (EAPO)**

**Lane 10: 25 mM with 0.125mg/ml ethyl acetate *P. orientalis* (EAPO)**

**Lane 11: 50 mM with 0.03125mg/ml ethyl acetate *P. orientalis* (EAPO)**

**Lane 12: 50 mM with 0.0625mg/ml ethyl acetate *P. orientalis* (EAPO)**

**Lane 13: 50 mM with 0.125mg/ml ethyl acetate *P. orientalis* (EAPO)**

**245**

**180**

**140**

**100**

**75**

**60**

**45**

**35**

**25**

**20**

**15**

**10**

**5**

Phospho-VEGFR2-130kDa

Beta actin -42kDa

**Supplement 8.** Gel blot images for phospho-VEGFR2 (130 kDa) and beta actin (42 kDa) expression in respective whole cell lysate, cytoplasmic, and nuclei fractions of retinal Müller cells. (Corresponds to Figure 5C)

Whole cell lysate

**1 2 3 4 5 6 7 8 9 10 11 12 13**

**245**

**180**

**140**

**100**

**75**

**60**

**45**

**35**

**25**

**20**

**15**

**10**

**5**


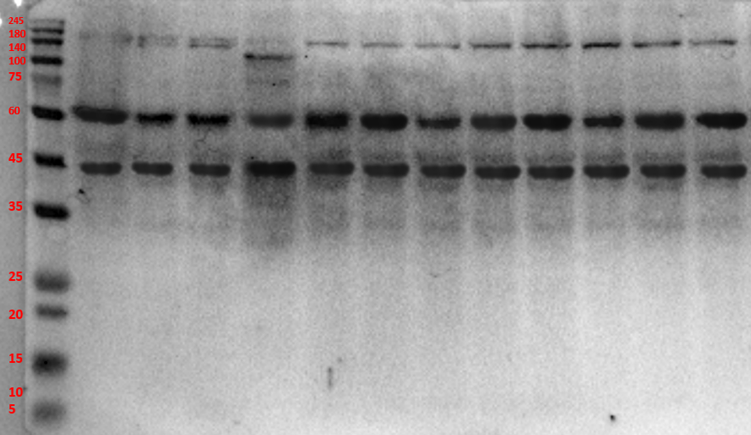


**Lane 1: ladder**

**Lane 2 5.5mM glucose**

**Lane 3: 25mM glucose**

**Lane 4: 50mM glucose**

**Lane 5: 5.5 mM with 0.03125mg/ml ethyl acetate *P. orientalis* (EAPO)**

**Lane 6: 5.5 mM with 0.0625mg/ml ethyl acetate *P. orientalis* (EAPO)**

**Lane 7: 5.5 mM with 0.125mg/ml ethyl acetate *P. orientalis* (EAPO)**

**Lane 8: 25 mM with 0.03125mg/ml ethyl acetate *P. orientalis* (EAPO)**

**Lane 9: 25 mM with 0.0625mg/ml ethyl acetate *P. orientalis* (EAPO)**

**Lane 10: 25 mM with 0.125mg/ml ethyl acetate *P. orientalis* (EAPO)**

**Lane 11: 50 mM with 0.03125mg/ml ethyl acetate *P. orientalis* (EAPO)**

**Lane 12: 50 mM with 0.0625mg/ml ethyl acetate *P. orientalis* (EAPO)**

**Lane 13: 50 mM with 0.125mg/ml ethyl acetate *P. orientalis* (EAPO)**

AKT1-56kDa

Beta actin -42kDa

Cytoplasmic fraction

**1 2 3 4 5 6 7 8 9 10 11 12 13**


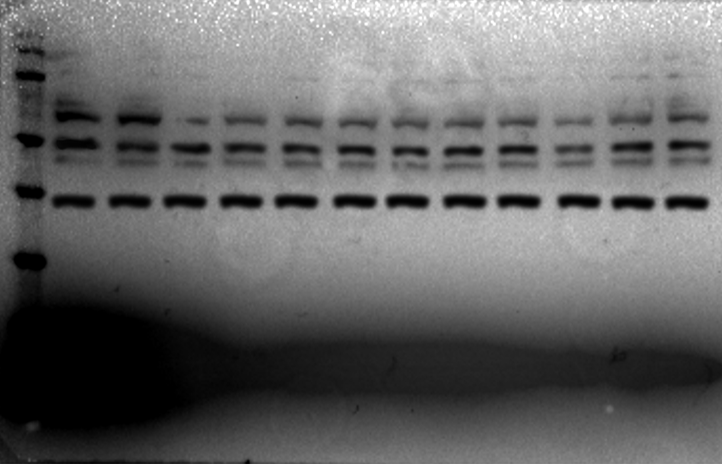


Beta actin -42kDa

AKT1-56kDa

**Lane 1: ladder**

**Lane 2 5.5mM glucose**

**Lane 3: 25mM glucose**

**Lane 4: 50mM glucose**

**Lane 5: 5.5 mM with 0.03125mg/ml ethyl acetate *P. orientalis* (EAPO)**

**Lane 6: 5.5 mM with 0.0625mg/ml ethyl acetate *P. orientalis* (EAPO)**

**Lane 7: 5.5 mM with 0.125mg/ml ethyl acetate *P. orientalis* (EAPO)**

**Lane 8: 25 mM with 0.03125mg/ml ethyl acetate *P. orientalis* (EAPO)**

**Lane 9: 25 mM with 0.0625mg/ml ethyl acetate *P. orientalis* (EAPO)**

**Lane 10: 25 mM with 0.125mg/ml ethyl acetate *P. orientalis* (EAPO)**

**Lane 11: 50 mM with 0.03125mg/ml ethyl acetate *P. orientalis* (EAPO)**

**Lane 12: 50 mM with 0.0625mg/ml ethyl acetate *P. orientalis* (EAPO)**

**Lane 13: 50 mM with 0.125mg/ml ethyl acetate *P. orientalis* (EAPO)**

**245**

**180**

**140**

**100**

**75**

**60**

**45**

**35**

**25**

**20**

**15**

**10**

**5**

Nuclei fraction

**1 2 3 4 5 6 7 8 9 10 11 12 13**


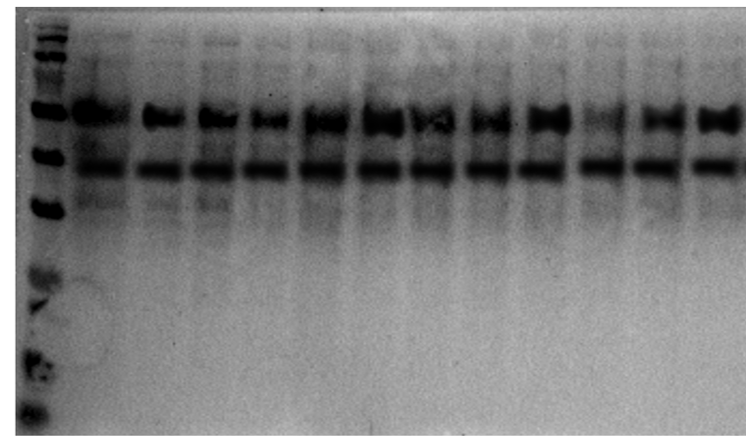


AKT1-56kDa

Beta actin -42kDa

**Lane 1: ladder**

**Lane 2 5.5mM glucose**

**Lane 3: 25mM glucose**

**Lane 4: 50mM glucose**

**Lane 5: 5.5 mM with 0.03125mg/ml ethyl acetate *P. orientalis* (EAPO)**

**Lane 6: 5.5 mM with 0.0625mg/ml ethyl acetate *P. orientalis* (EAPO)**

**Lane 7: 5.5 mM with 0.125mg/ml ethyl acetate *P. orientalis* (EAPO)**

**Lane 8: 25 mM with 0.03125mg/ml ethyl acetate *P. orientalis* (EAPO)**

**Lane 9: 25 mM with 0.0625mg/ml ethyl acetate *P. orientalis* (EAPO)**

**Lane 10: 25 mM with 0.125mg/ml ethyl acetate *P. orientalis* (EAPO)**

**Lane 11: 50 mM with 0.03125mg/ml ethyl acetate *P. orientalis* (EAPO)**

**Lane 12: 50 mM with 0.0625mg/ml ethyl acetate *P. orientalis* (EAPO)**

**Lane 13: 50 mM with 0.125mg/ml ethyl acetate *P. orientalis* (EAPO)**

**245**

**180**

**140**

**100**

**75**

**60**

**45**

**35**

**25**

**20**

**15**

**10**

**5**

**Supplement 9.** Gel blot images for AKT1 (56 kDa) and beta actin (42 kDa) expression in respective whole cell lysate, cytoplasmic, and nuclei fractions of retinal Müller cells (Corresponds to Figure 6A)

Whole cell lysate

**1 2 3 4 5 6 7 8 9 10 11 12 13**


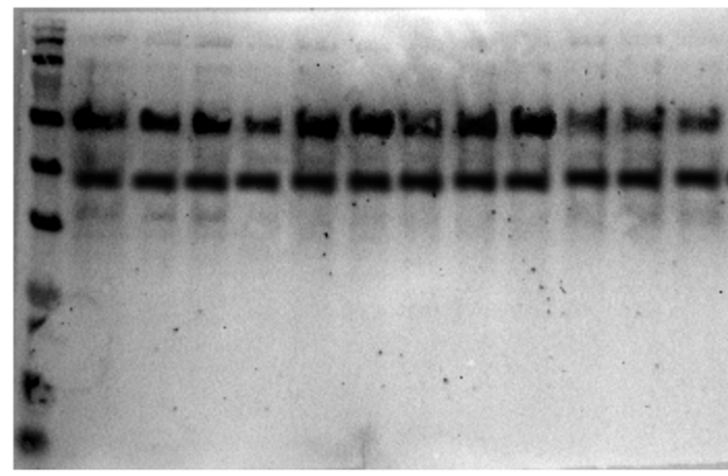


Phospho-AKT1-55kDa

**Lane 1: ladder**

**Lane 2 5.5mM glucose**

**Lane 3: 25mM glucose**

**Lane 4: 50mM glucose**

**Lane 5: 5.5 mM with 0.03125mg/ml ethyl acetate *P. orientalis* (EAPO)**

**Lane 6: 5.5 mM with 0.0625mg/ml ethyl acetate *P. orientalis* (EAPO)**

**Lane 7: 5.5 mM with 0.125mg/ml ethyl acetate *P. orientalis* (EAPO)**

**Lane 8: 25 mM with 0.03125mg/ml ethyl acetate *P. orientalis* (EAPO)**

**Lane 9: 25 mM with 0.0625mg/ml ethyl acetate *P. orientalis* (EAPO)**

**Lane 10: 25 mM with 0.125mg/ml ethyl acetate *P. orientalis* (EAPO)**

**Lane 11: 50 mM with 0.03125mg/ml ethyl acetate *P. orientalis* (EAPO)**

**Lane 12: 50 mM with 0.0625mg/ml ethyl acetate *P. orientalis* (EAPO)**

**Lane 13: 50 mM with 0.125mg/ml ethyl acetate *P. orientalis* (EAPO)**

**245**

**180**

**140**

**100**

**75**

**60**

**45**

**35**

**25**

**20**

**15**

**10**

**5**

Beta actin -42kDa

Cytoplasmic fraction

**1 2 3 4 5 6 7 8 9 10 11 12 13**

**Lane 1: ladder**

**Lane 2 5.5mM glucose**

**Lane 3: 25mM glucose**

**Lane 4: 50mM glucose**

**Lane 5: 5.5 mM with 0.03125mg/ml ethyl acetate *P. orientalis* (EAPO)**

**Lane 6: 5.5 mM with 0.0625mg/ml ethyl acetate *P. orientalis* (EAPO)**

**Lane 7: 5.5 mM with 0.125mg/ml ethyl acetate *P. orientalis* (EAPO)**

**Lane 8: 25 mM with 0.03125mg/ml ethyl acetate *P. orientalis* (EAPO)**

**Lane 9: 25 mM with 0.0625mg/ml ethyl acetate *P. orientalis* (EAPO)**

**Lane 10: 25 mM with 0.125mg/ml ethyl acetate *P. orientalis* (EAPO)**

**Lane 11: 50 mM with 0.03125mg/ml ethyl acetate *P. orientalis* (EAPO)**

**Lane 12: 50 mM with 0.0625mg/ml ethyl acetate *P. orientalis* (EAPO)**

**Lane 13: 50 mM with 0.125mg/ml ethyl acetate *P. orientalis* (EAPO)**


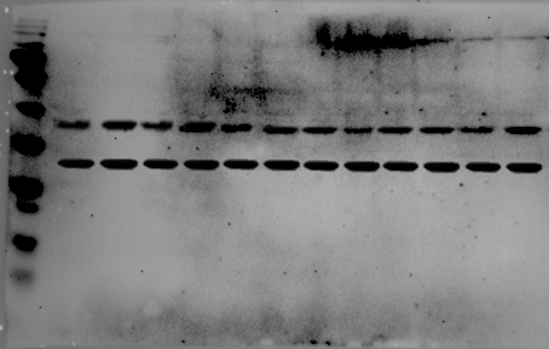


**245**

**180**

**140**

**100**

**75**

**60**

**45**

**35**

**25**

**20**

**15**

**10**

**5**

Phospho-AKT1-55kDa

Beta actin -42kDa


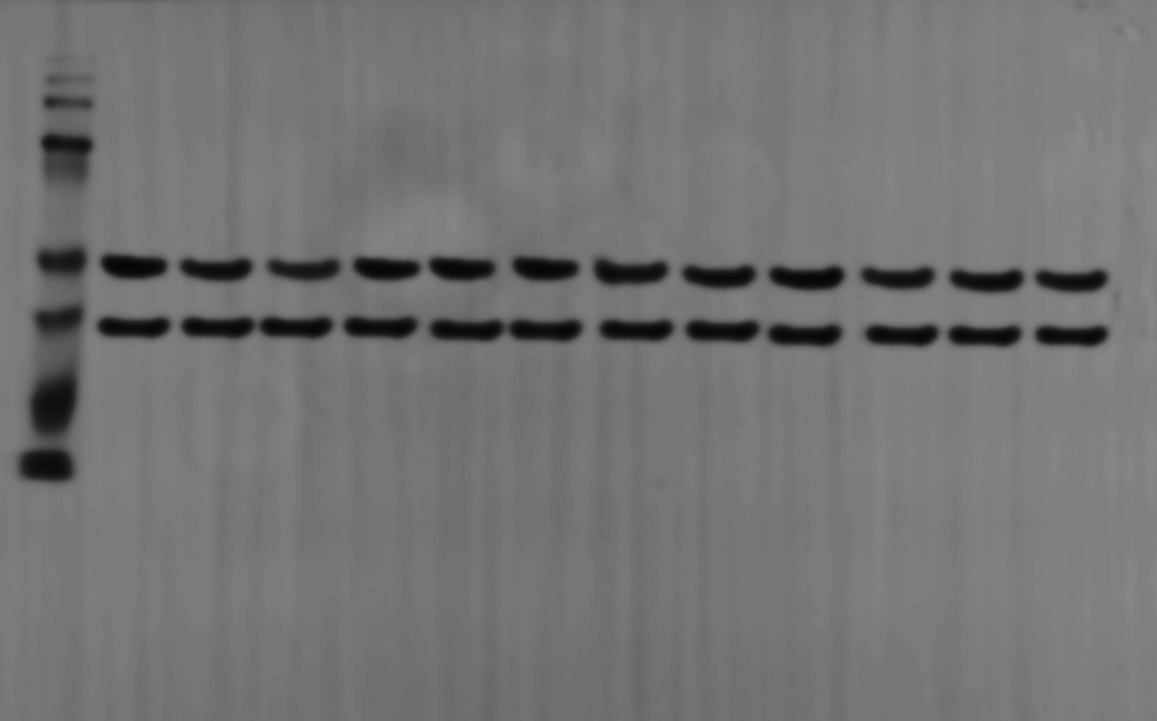
Nuclei fraction

**1 2 3 4 5 6 7 8 9 10 11 12 13**

**245**

**180**

**140**

**100**

**75**

**60**

**45**

**35**

**25**

**20**

**15**

**10**

**5**

**Lane 1: ladder**

**Lane 2 5.5mM glucose**

**Lane 3: 25mM glucose**

**Lane 4: 50mM glucose**

**Lane 5: 5.5 mM with 0.03125mg/ml ethyl acetate *P. orientalis* (EAPO)**

**Lane 6: 5.5 mM with 0.0625mg/ml ethyl acetate *P. orientalis* (EAPO)**

**Lane 7: 5.5 mM with 0.125mg/ml ethyl acetate *P. orientalis* (EAPO)**

**Lane 8: 25 mM with 0.03125mg/ml ethyl acetate *P. orientalis* (EAPO)**

**Lane 9: 25 mM with 0.0625mg/ml ethyl acetate *P. orientalis* (EAPO)**

**Lane 10: 25 mM with 0.125mg/ml ethyl acetate *P. orientalis* (EAPO)**

**Lane 11: 50 mM with 0.03125mg/ml ethyl acetate *P. orientalis* (EAPO)**

**Lane 12: 50 mM with 0.0625mg/ml ethyl acetate *P. orientalis* (EAPO)**

**Lane 13: 50 mM with 0.125mg/ml ethyl acetate *P. orientalis* (EAPO)**

Phospho-AKT1-55kDa

Beta actin -42kDa

**Supplement 10.** Gel blot images for phospho-AKT1 (55 kDa) and beta actin (42 kDa) expression in respective whole cell lysate, cytoplasmic, and nuclei fractions of retinal Müller cells. (Corresponds to Figure 6B)

Whole cell lysate

**1 2 3 4 5 6 7 8 9 10 11 12 13**

**Lane 1: ladder**

**Lane 2 5.5mM glucose**

**Lane 3: 25mM glucose**

**Lane 4: 50mM glucose**

**Lane 5: 5.5 mM with 0.03125mg/ml ethyl acetate *P. orientalis* (EAPO)**

**Lane 6: 5.5 mM with 0.0625mg/ml ethyl acetate *P. orientalis* (EAPO)**

**Lane 7: 5.5 mM with 0.125mg/ml ethyl acetate *P. orientalis* (EAPO)**

**Lane 8: 25 mM with 0.03125mg/ml ethyl acetate *P. orientalis* (EAPO)**

**Lane 9: 25 mM with 0.0625mg/ml ethyl acetate *P. orientalis* (EAPO)**

**Lane 10: 25 mM with 0.125mg/ml ethyl acetate *P. orientalis* (EAPO)**

**Lane 11: 50 mM with 0.03125mg/ml ethyl acetate *P. orientalis* (EAPO)**

**Lane 12: 50 mM with 0.0625mg/ml ethyl acetate *P. orientalis* (EAPO)**

**Lane 13: 50 mM with 0.125mg/ml ethyl acetate *P. orientalis* (EAPO)**


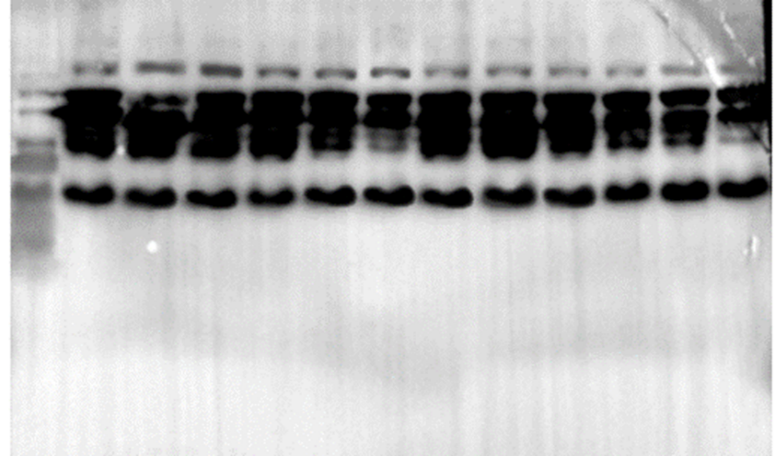


mTOR-289kDa

Beta actin -42kDa

**245**

**180**

**140**

**100**

**75**

**60**

**45**

**35**

**25**

**20**

**15**

**10**

**5**

Cytoplasmic fraction

**1 2 3 4 5 6 7 8 9 10 11 12 13**


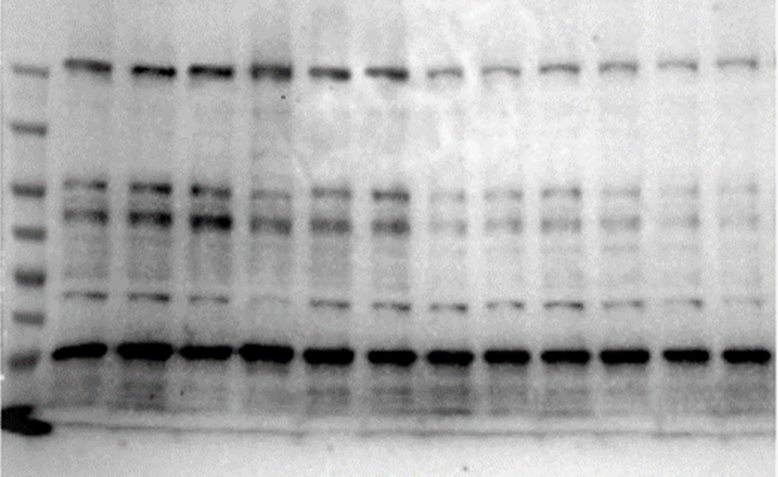


mTOR-289kDa

Beta actin -42kDa

**Lane 1: ladder**

**Lane 2 5.5mM glucose**

**Lane 3: 25mM glucose**

**Lane 4: 50mM glucose**

**Lane 5: 5.5 mM with 0.03125mg/ml ethyl acetate *P. orientalis* (EAPO)**

**Lane 6: 5.5 mM with 0.0625mg/ml ethyl acetate *P. orientalis* (EAPO)**

**Lane 7: 5.5 mM with 0.125mg/ml ethyl acetate *P. orientalis* (EAPO)**

**Lane 8: 25 mM with 0.03125mg/ml ethyl acetate *P. orientalis* (EAPO)**

**Lane 9: 25 mM with 0.0625mg/ml ethyl acetate *P. orientalis* (EAPO)**

**Lane 10: 25 mM with 0.125mg/ml ethyl acetate *P. orientalis* (EAPO)**

**Lane 11: 50 mM with 0.03125mg/ml ethyl acetate *P. orientalis* (EAPO)**

**Lane 12: 50 mM with 0.0625mg/ml ethyl acetate *P. orientalis* (EAPO)**

**Lane 13: 50 mM with 0.125mg/ml ethyl acetate *P. orientalis* (EAPO)**

**245**

**180**

**140**

**100**

**75**

**60**

**45**

**35**

**25**

**20**

**15**

**10**

**5**

Nuclei fraction

**1 2 3 4 5 6 7 8 9 10 11 12 13**


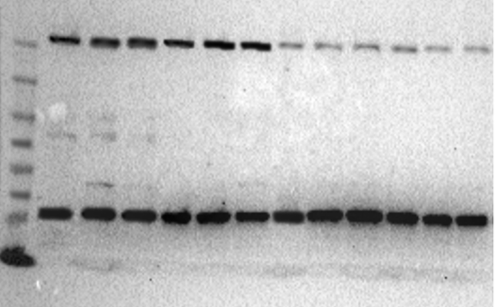


**245**

**180**

**140**

**100**

**75**

**60**

**45**

**35**

**25**

**20**

**15**

**10**

**5**

mTOR-289kDa

Beta actin -42kDa

**Lane 1: ladder**

**Lane 2 5.5mM glucose**

**Lane 3: 25mM glucose**

**Lane 4: 50mM glucose**

**Lane 5: 5.5 mM with 0.03125mg/ml ethyl acetate *P. orientalis* (EAPO)**

**Lane 6: 5.5 mM with 0.0625mg/ml ethyl acetate *P. orientalis* (EAPO)**

**Lane 7: 5.5 mM with 0.125mg/ml ethyl acetate *P. orientalis* (EAPO)**

**Lane 8: 25 mM with 0.03125mg/ml ethyl acetate *P. orientalis* (EAPO)**

**Lane 9: 25 mM with 0.0625mg/ml ethyl acetate *P. orientalis* (EAPO)**

**Lane 10: 25 mM with 0.125mg/ml ethyl acetate *P. orientalis* (EAPO)**

**Lane 11: 50 mM with 0.03125mg/ml ethyl acetate *P. orientalis* (EAPO)**

**Lane 12: 50 mM with 0.0625mg/ml ethyl acetate *P. orientalis* (EAPO)**

**Lane 13: 50 mM with 0.125mg/ml ethyl acetate *P. orientalis* (EAPO)**

**Supplement 11.** Gel blot images for mTOR (289 kDa) and beta actin (42 kDa) expression in respective whole cell lysate, cytoplasmic, and nuclei fractions of retinal Müller cells. (Corresponds to Figure 6C)

Whole cell lysate

**1 2 3 4 5 6 7 8 9 10 11 12 13**


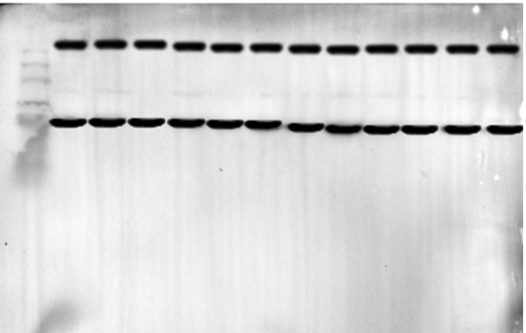


**245**

**180**

**140**

**100**

**75**

**60**

**45**

**35**

**25**

**20**

**15**

**10**

**5**

Phospho-mTOR-288kDa

Beta actin -42kDa

**Lane 1: ladder**

**Lane 2 5.5mM glucose**

**Lane 3: 25mM glucose**

**Lane 4: 50mM glucose**

**Lane 5: 5.5 mM with 0.03125mg/ml ethyl acetate *P. orientalis* (EAPO)**

**Lane 6: 5.5 mM with 0.0625mg/ml ethyl acetate *P. orientalis* (EAPO)**

**Lane 7: 5.5 mM with 0.125mg/ml ethyl acetate *P. orientalis* (EAPO)**

**Lane 8: 25 mM with 0.03125mg/ml ethyl acetate *P. orientalis* (EAPO)**

**Lane 9: 25 mM with 0.0625mg/ml ethyl acetate *P. orientalis* (EAPO)**

**Lane 10: 25 mM with 0.125mg/ml ethyl acetate *P. orientalis* (EAPO)**

**Lane 11: 50 mM with 0.03125mg/ml ethyl acetate *P. orientalis* (EAPO)**

**Lane 12: 50 mM with 0.0625mg/ml ethyl acetate *P. orientalis* (EAPO)**

**Lane 13: 50 mM with 0.125mg/ml ethyl acetate *P. orientalis* (EAPO)**

Cytoplasmic fraction

**1 2 3 4 5 6 7 8 9 10 11 12 13**


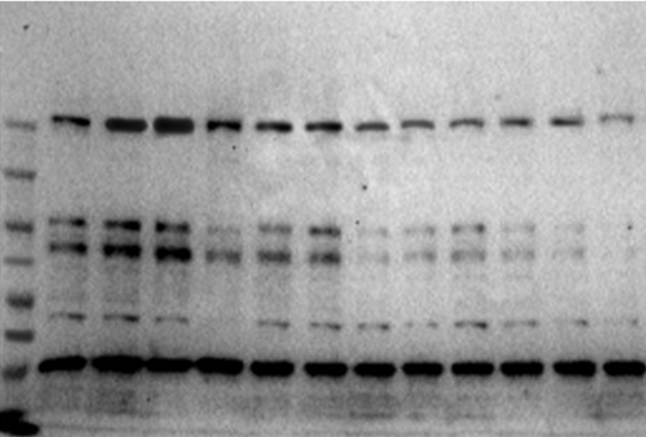


**245**

**180**

**140**

**100**

**75**

**60**

**45**

**35**

**25**

**20**

**15**

**10**

**5**

Beta actin -42kDa

Phospho-mTOR-288kDa

**Lane 1: ladder**

**Lane 2 5.5mM glucose**

**Lane 3: 25mM glucose**

**Lane 4: 50mM glucose**

**Lane 5: 5.5 mM with 0.03125mg/ml ethyl acetate *P. orientalis* (EAPO)**

**Lane 6: 5.5 mM with 0.0625mg/ml ethyl acetate *P. orientalis* (EAPO)**

**Lane 7: 5.5 mM with 0.125mg/ml ethyl acetate *P. orientalis* (EAPO)**

**Lane 8: 25 mM with 0.03125mg/ml ethyl acetate *P. orientalis* (EAPO)**

**Lane 9: 25 mM with 0.0625mg/ml ethyl acetate *P. orientalis* (EAPO)**

**Lane 10: 25 mM with 0.125mg/ml ethyl acetate *P. orientalis* (EAPO)**

**Lane 11: 50 mM with 0.03125mg/ml ethyl acetate *P. orientalis* (EAPO)**

**Lane 12: 50 mM with 0.0625mg/ml ethyl acetate *P. orientalis* (EAPO)**

**Lane 13: 50 mM with 0.125mg/ml ethyl acetate *P. orientalis* (EAPO)**


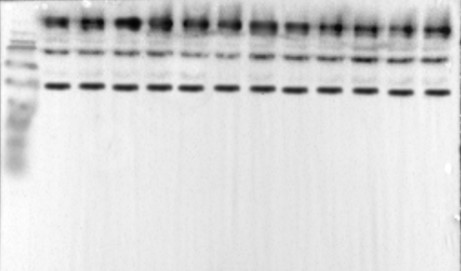
Nuclei fraction

**Lane 1: ladder**

**Lane 2 5.5mM glucose**

**Lane 3: 25mM glucose**

**Lane 4: 50mM glucose**

**Lane 5: 5.5 mM with 0.03125mg/ml ethyl acetate *P. orientalis* (EAPO)**

**Lane 6: 5.5 mM with 0.0625mg/ml ethyl acetate *P. orientalis* (EAPO)**

**Lane 7: 5.5 mM with 0.125mg/ml ethyl acetate *P. orientalis* (EAPO)**

**Lane 8: 25 mM with 0.03125mg/ml ethyl acetate *P. orientalis* (EAPO)**

**Lane 9: 25 mM with 0.0625mg/ml ethyl acetate *P. orientalis* (EAPO)**

**Lane 10: 25 mM with 0.125mg/ml ethyl acetate *P. orientalis* (EAPO)**

**Lane 11: 50 mM with 0.03125mg/ml ethyl acetate *P. orientalis* (EAPO)**

**Lane 12: 50 mM with 0.0625mg/ml ethyl acetate *P. orientalis* (EAPO)**

**Lane 13: 50 mM with 0.125mg/ml ethyl acetate *P. orientalis* (EAPO)**

**1 2 3 4 5 6 7 8 9 10 11 12 13**

Phospho-mTOR-288kDa

**245**

**180**

**140**

**100**

**75**

**60**

**45**

**35**

**25**

**20**

**15**

**10**

**5**

Beta actin -42kDa

**Supplement 12.** Gel blot images for phospho-mTOR (288 kDa) and beta actin (42 kDa) expression in respective whole cell lysate, cytoplasmic, and nuclei fractions of retinal Müller cells. (Corresponds to Figure 6D)

Whole cell lysate

**1 2 3 4 5 6 7 8 9 10 11 12 13**

**245**

**180**

**140**

**100**

**75**

**60**

**45**

**35**

**25**

**20**

**15**

**10**

**5**


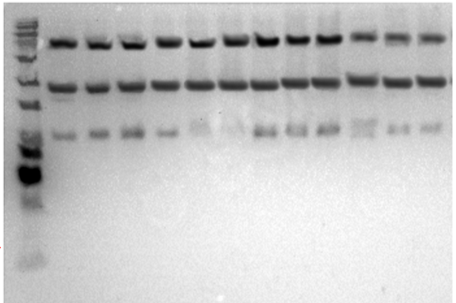


Beta actin -42kDa

Raf-1-75kDa

**Lane 1: ladder**

**Lane 2 5.5mM glucose**

**Lane 3: 25mM glucose**

**Lane 4: 50mM glucose**

**Lane 5: 5.5 mM with 0.03125mg/ml ethyl acetate *P. orientalis* (EAPO)**

**Lane 6: 5.5 mM with 0.0625mg/ml ethyl acetate *P. orientalis* (EAPO)**

**Lane 7: 5.5 mM with 0.125mg/ml ethyl acetate *P. orientalis* (EAPO)**

**Lane 8: 25 mM with 0.03125mg/ml ethyl acetate *P. orientalis* (EAPO)**

**Lane 9: 25 mM with 0.0625mg/ml ethyl acetate *P. orientalis* (EAPO)**

**Lane 10: 25 mM with 0.125mg/ml ethyl acetate *P. orientalis* (EAPO)**

**Lane 11: 50 mM with 0.03125mg/ml ethyl acetate *P. orientalis* (EAPO)**

**Lane 12: 50 mM with 0.0625mg/ml ethyl acetate *P. orientalis* (EAPO)**

**Lane 13: 50 mM with 0.125mg/ml ethyl acetate *P. orientalis* (EAPO)**

Cytoplasmic fraction

**1 2 3 4 5 6 7 8 9 10 11 12 13**


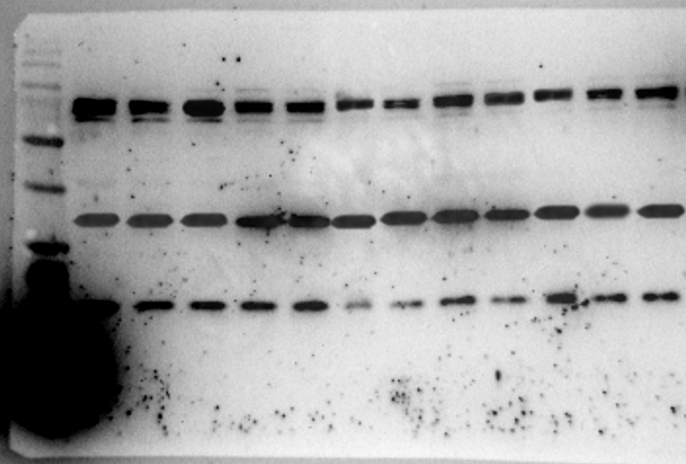


**Lane 1: ladder**

**Lane 2 5.5mM glucose**

**Lane 3: 25mM glucose**

**Lane 4: 50mM glucose**

**Lane 5: 5.5 mM with 0.03125mg/ml ethyl acetate *P. orientalis* (EAPO)**

**Lane 6: 5.5 mM with 0.0625mg/ml ethyl acetate *P. orientalis* (EAPO)**

**Lane 7: 5.5 mM with 0.125mg/ml ethyl acetate *P. orientalis* (EAPO)**

**Lane 8: 25 mM with 0.03125mg/ml ethyl acetate *P. orientalis* (EAPO)**

**Lane 9: 25 mM with 0.0625mg/ml ethyl acetate *P. orientalis* (EAPO)**

**Lane 10: 25 mM with 0.125mg/ml ethyl acetate *P. orientalis* (EAPO)**

**Lane 11: 50 mM with 0.03125mg/ml ethyl acetate *P. orientalis* (EAPO)**

**Lane 12: 50 mM with 0.0625mg/ml ethyl acetate *P. orientalis* (EAPO)**

**Lane 13: 50 mM with 0.125mg/ml ethyl acetate *P. orientalis* (EAPO)**

Beta actin -42kDa

Raf-1-75kDa

**245**

**180**

**140**

**100**

**75**

**60**

**45**

**35**

**25**

**20**

**15**

**10**

**5**

Nuclei fraction

**1 2 3 4 5 6 7 8 9 10 11 12 13**

**245**

**180**

**140**

**100**

**75**

**60**

**45**

**35**

**25**

**20**

**15**

**10**

**5**


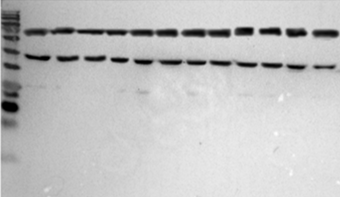


Raf-1-75kDa

Beta actin -42kDa

**Lane 1: ladder**

**Lane 2 5.5mM glucose**

**Lane 3: 25mM glucose**

**Lane 4: 50mM glucose**

**Lane 5: 5.5 mM with 0.03125mg/ml ethyl acetate *P. orientalis* (EAPO)**

**Lane 6: 5.5 mM with 0.0625mg/ml ethyl acetate *P. orientalis* (EAPO)**

**Lane 7: 5.5 mM with 0.125mg/ml ethyl acetate *P. orientalis* (EAPO)**

**Lane 8: 25 mM with 0.03125mg/ml ethyl acetate *P. orientalis* (EAPO)**

**Lane 9: 25 mM with 0.0625mg/ml ethyl acetate *P. orientalis* (EAPO)**

**Lane 10: 25 mM with 0.125mg/ml ethyl acetate *P. orientalis* (EAPO)**

**Lane 11: 50 mM with 0.03125mg/ml ethyl acetate *P. orientalis* (EAPO)**

**Lane 12: 50 mM with 0.0625mg/ml ethyl acetate *P. orientalis* (EAPO)**

**Lane 13: 50 mM with 0.125mg/ml ethyl acetate *P. orientalis* (EAPO)**

**Supplement 13.** Gel blot images for Raf-1 (75 kDa) and beta actin (42 kDa) expression in respective whole cell lysate, cytoplasmic, and nuclei fractions of retinal Müller cells. (Corresponds to Figure 7A)

Whole cell lysate

**1 2 3 4 5 6 7 8 9 10 11 12 13**

**245**

**180**

**140**

**100**

**75**

**60**

**45**

**35**

**25**

**20**

**15**

**10**

**5**


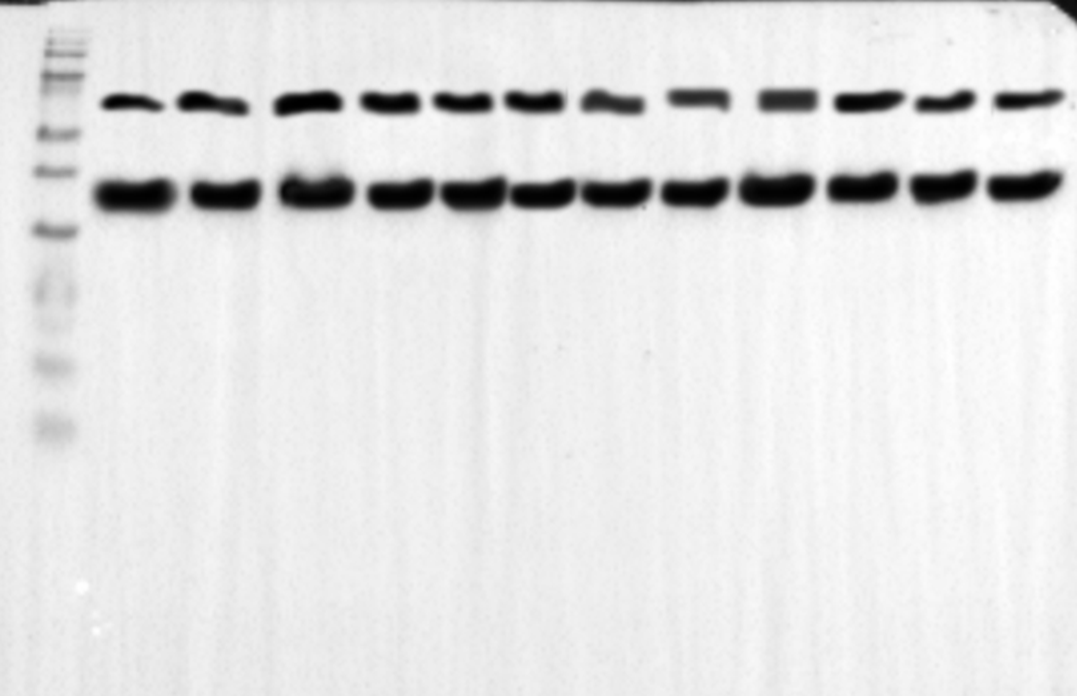


**Lane 1: ladder**

**Lane 2 5.5mM glucose**

**Lane 3: 25mM glucose**

**Lane 4: 50mM glucose**

**Lane 5: 5.5 mM with 0.03125mg/ml ethyl acetate *P. orientalis* (EAPO)**

**Lane 6: 5.5 mM with 0.0625mg/ml ethyl acetate *P. orientalis* (EAPO)**

**Lane 7: 5.5 mM with 0.125mg/ml ethyl acetate *P. orientalis* (EAPO)**

**Lane 8: 25 mM with 0.03125mg/ml ethyl acetate *P. orientalis* (EAPO)**

**Lane 9: 25 mM with 0.0625mg/ml ethyl acetate *P. orientalis* (EAPO)**

**Lane 10: 25 mM with 0.125mg/ml ethyl acetate *P. orientalis* (EAPO)**

**Lane 11: 50 mM with 0.03125mg/ml ethyl acetate *P. orientalis* (EAPO)**

**Lane 12: 50 mM with 0.0625mg/ml ethyl acetate *P. orientalis* (EAPO)**

**Lane 13: 50 mM with 0.125mg/ml ethyl acetate *P. orientalis* (EAPO)**

**Lane 1: ladder**

**Lane 2 5.5mM glucose**

**Lane 3: 25mM glucose**

**Lane 4: 50mM glucose**

**Lane 5: 5.5 mM with 0.03125mg/ml ethyl acetate *P. orientalis* (EAPO)**

**Lane 6: 5.5 mM with 0.0625mg/ml ethyl acetate *P. orientalis* (EAPO)**

**Lane 7: 5.5 mM with 0.125mg/ml ethyl acetate *P. orientalis* (EAPO)**

**Lane 8: 25 mM with 0.03125mg/ml ethyl acetate *P. orientalis* (EAPO)**

**Lane 9: 25 mM with 0.0625mg/ml ethyl acetate *P. orientalis* (EAPO)**

**Lane 10: 25 mM with 0.125mg/ml ethyl acetate *P. orientalis* (EAPO)**

**Lane 11: 50 mM with 0.03125mg/ml ethyl acetate *P. orientalis* (EAPO)**

**Lane 12: 50 mM with 0.0625mg/ml ethyl acetate *P. orientalis* (EAPO)**

**Lane 13: 50 mM with 0.125mg/ml ethyl acetate *P. orientalis* (EAPO)**

Phospho-Raf-1-72kDa

Beta actin -42kDa

Cytoplasmic fraction

**1 2 3 4 5 6 7 8 9 10 11 12 13**


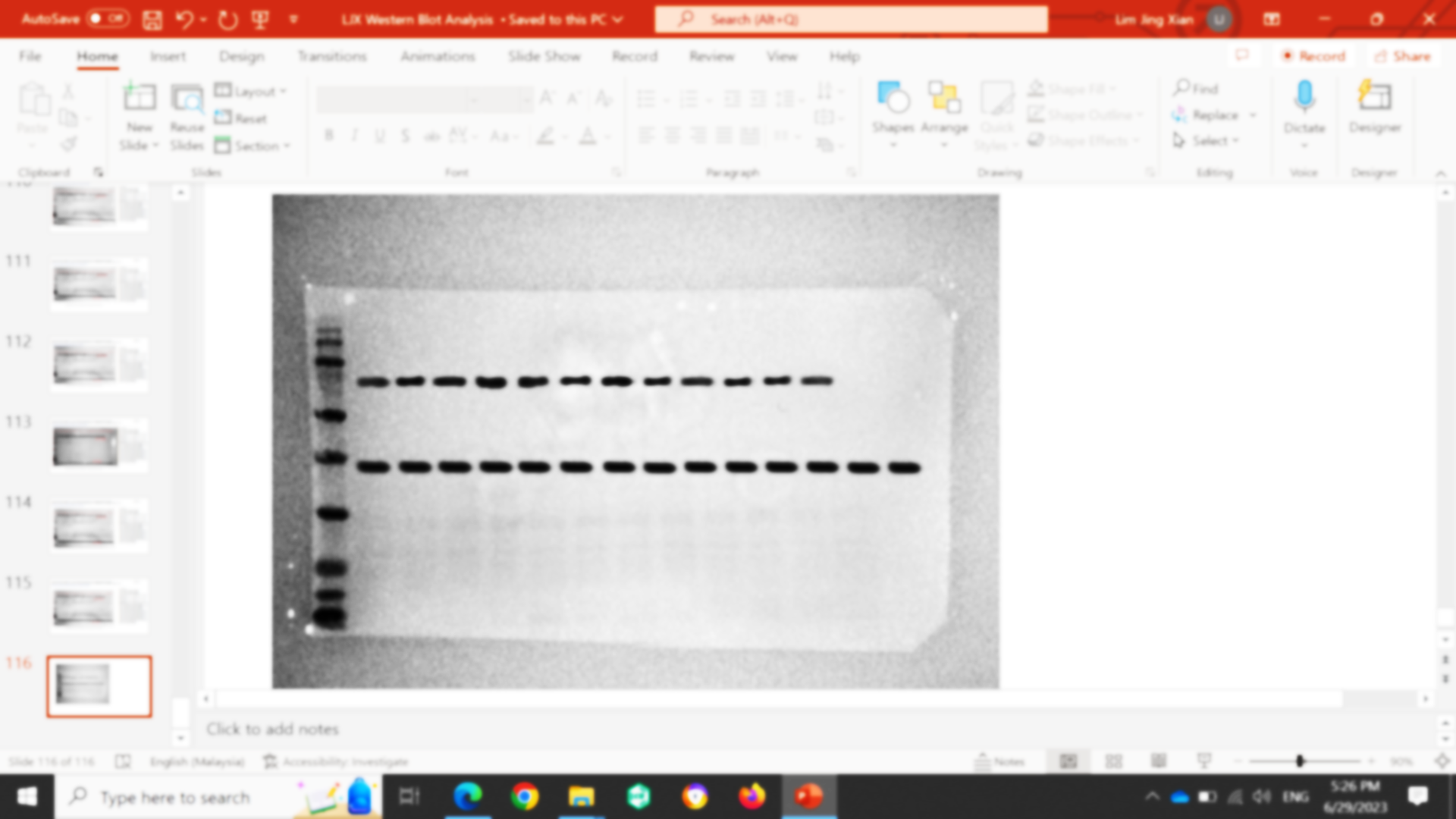


**Lane 1: ladder**

**Lane 2 5.5mM glucose**

**Lane 3: 25mM glucose**

**Lane 4: 50mM glucose**

**Lane 5: 5.5 mM with 0.03125mg/ml ethyl acetate *P. orientalis* (EAPO)**

**Lane 6: 5.5 mM with 0.0625mg/ml ethyl acetate *P. orientalis* (EAPO)**

**Lane 7: 5.5 mM with 0.125mg/ml ethyl acetate *P. orientalis* (EAPO)**

**Lane 8: 25 mM with 0.03125mg/ml ethyl acetate *P. orientalis* (EAPO)**

**Lane 9: 25 mM with 0.0625mg/ml ethyl acetate *P. orientalis* (EAPO)**

**Lane 10: 25 mM with 0.125mg/ml ethyl acetate *P. orientalis* (EAPO)**

**Lane 11: 50 mM with 0.03125mg/ml ethyl acetate *P. orientalis* (EAPO)**

**Lane 12: 50 mM with 0.0625mg/ml ethyl acetate *P. orientalis* (EAPO)**

**Lane 13: 50 mM with 0.125mg/ml ethyl acetate *P. orientalis* (EAPO)**

**245**

**180**

**140**

**100**

**75**

**60**

**45**

**35**

**25**

**20**

**15**

**10**

**5**

Phospho-Raf-1-72kDa

Beta actin -42kDa

Nuclei fraction

**1 2 3 4 5 6 7 8 9 10 11 12 13**


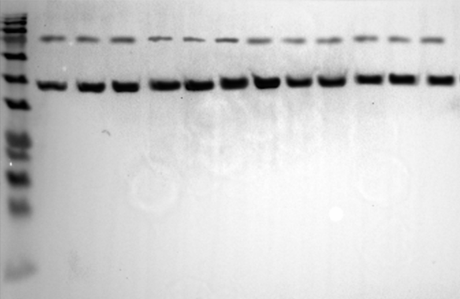


**245**

**180**

**140**

**100**

**75**

**60**

**45**

**35**

**25**

**20**

**15**

**10**

**5**

Phospho-Raf-1-72kDa

Beta actin -42kDa

**Supplement 14.** Gel blot images for phospho-Raf-1 (72 kDa) and beta actin (42 kDa) expression in respective whole cell lysate, cytoplasmic, and nuclei fractions of retinal Müller cells. (Corresponds to Figure 7B)


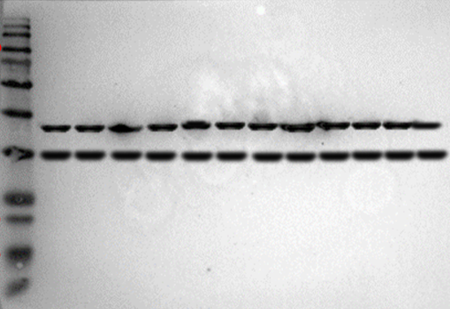
Whole cell lysate

**1 2 3 4 5 6 7 8 9 10 11 12 13**

**245**

**180**

**140**

**100**

**75**

**60**

**45**

**35**

**25**

**20**

**15**

**10**

**5**

**Lane 1: ladder**

**Lane 2 5.5mM glucose**

**Lane 3: 25mM glucose**

**Lane 4: 50mM glucose**

**Lane 5: 5.5 mM with 0.03125mg/ml ethyl acetate *P. orientalis* (EAPO)**

**Lane 6: 5.5 mM with 0.0625mg/ml ethyl acetate *P. orientalis* (EAPO)**

**Lane 7: 5.5 mM with 0.125mg/ml ethyl acetate *P. orientalis* (EAPO)**

**Lane 8: 25 mM with 0.03125mg/ml ethyl acetate *P. orientalis* (EAPO)**

**Lane 9: 25 mM with 0.0625mg/ml ethyl acetate *P. orientalis* (EAPO)**

**Lane 10: 25 mM with 0.125mg/ml ethyl acetate *P. orientalis* (EAPO)**

**Lane 11: 50 mM with 0.03125mg/ml ethyl acetate *P. orientalis* (EAPO)**

**Lane 12: 50 mM with 0.0625mg/ml ethyl acetate *P. orientalis* (EAPO)**

**Lane 13: 50 mM with 0.125mg/ml ethyl acetate *P. orientalis* (EAPO)**

MEK1/2– 45kDa

GAPDH-35kDa


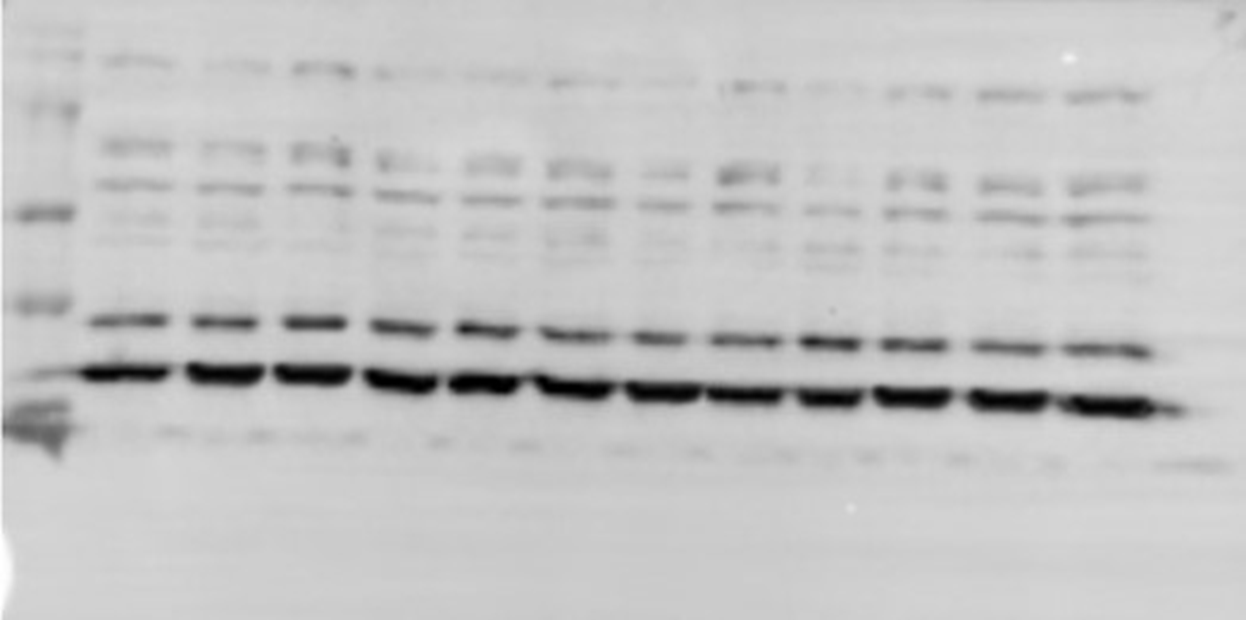
Cytoplasmic fraction

**1 2 3 4 5 6 7 8 9 10 11 12 13**

**Lane 1: ladder**

**Lane 2 5.5mM glucose**

**Lane 3: 25mM glucose**

**Lane 4: 50mM glucose**

**Lane 5: 5.5 mM with 0.03125mg/ml ethyl acetate *P. orientalis* (EAPO)**

**Lane 6: 5.5 mM with 0.0625mg/ml ethyl acetate *P. orientalis* (EAPO)**

**Lane 7: 5.5 mM with 0.125mg/ml ethyl acetate *P. orientalis* (EAPO)**

**Lane 8: 25 mM with 0.03125mg/ml ethyl acetate *P. orientalis* (EAPO)**

**Lane 9: 25 mM with 0.0625mg/ml ethyl acetate *P. orientalis* (EAPO)**

**Lane 10: 25 mM with 0.125mg/ml ethyl acetate *P. orientalis* (EAPO)**

**Lane 11: 50 mM with 0.03125mg/ml ethyl acetate *P. orientalis* (EAPO)**

**Lane 12: 50 mM with 0.0625mg/ml ethyl acetate *P. orientalis* (EAPO)**

**Lane 13: 50 mM with 0.125mg/ml ethyl acetate *P. orientalis* (EAPO)**

**245**

**180**

**140**

**100**

**75**

**60**

**45**

**35**

**25**

**20**

**15**

**10**

**5**

MEK1/2– 45kDa

GAPDH-35kDa

Nuclei fraction

**1 2 3 4 5 6 7 8 9 10 11 12 13**

**245**

**180**

**140**

**100**

**75**

**60**

**45**

**35**

**25**

**20**

**15**

**10**

**5**


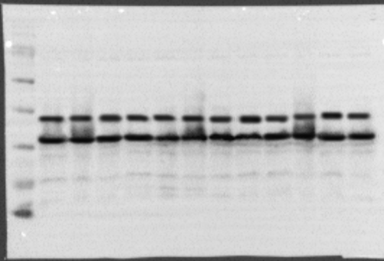


GAPDH-35kDa

**Lane 1: ladder**

**Lane 2 5.5mM glucose**

**Lane 3: 25mM glucose**

**Lane 4: 50mM glucose**

**Lane 5: 5.5 mM with 0.03125mg/ml ethyl acetate *P. orientalis* (EAPO)**

**Lane 6: 5.5 mM with 0.0625mg/ml ethyl acetate *P. orientalis* (EAPO)**

**Lane 7: 5.5 mM with 0.125mg/ml ethyl acetate *P. orientalis* (EAPO)**

**Lane 8: 25 mM with 0.03125mg/ml ethyl acetate *P. orientalis* (EAPO)**

**Lane 9: 25 mM with 0.0625mg/ml ethyl acetate *P. orientalis* (EAPO)**

**Lane 10: 25 mM with 0.125mg/ml ethyl acetate *P. orientalis* (EAPO)**

**Lane 11: 50 mM with 0.03125mg/ml ethyl acetate *P. orientalis* (EAPO)**

**Lane 12: 50 mM with 0.0625mg/ml ethyl acetate *P. orientalis* (EAPO)**

**Lane 13: 50 mM with 0.125mg/ml ethyl acetate *P. orientalis* (EAPO)**

MEK1/2– 45kDa

**Supplement 15.** Gel blot images for MEK1/2 (45 kDa) and GAPDH (36 kDa) expression in respective whole cell lysate, cytoplasmic, and nuclei fractions of retinal Müller cells. (Corresponds to Figure 7C)

Whole cell lysate

**245**

**180**

**140**

**100**

**75**

**60**

**45**

**35**

**25**

**20**

**15**

**10**

**5**

**1 2 3 4 5 6 7 8 9 10 11 12 13**


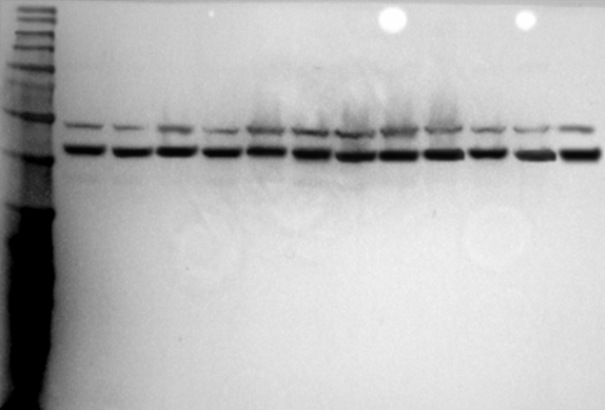


GAPDH-35kDa

Phospho-MEK1/2– 43kDa

**Lane 1: ladder**

**Lane 2 5.5mM glucose**

**Lane 3: 25mM glucose**

**Lane 4: 50mM glucose**

**Lane 5: 5.5 mM with 0.03125mg/ml ethyl acetate *P. orientalis* (EAPO)**

**Lane 6: 5.5 mM with 0.0625mg/ml ethyl acetate *P. orientalis* (EAPO)**

**Lane 7: 5.5 mM with 0.125mg/ml ethyl acetate *P. orientalis* (EAPO)**

**Lane 8: 25 mM with 0.03125mg/ml ethyl acetate *P. orientalis* (EAPO)**

**Lane 9: 25 mM with 0.0625mg/ml ethyl acetate *P. orientalis* (EAPO)**

**Lane 10: 25 mM with 0.125mg/ml ethyl acetate *P. orientalis* (EAPO)**

**Lane 11: 50 mM with 0.03125mg/ml ethyl acetate *P. orientalis* (EAPO)**

**Lane 12: 50 mM with 0.0625mg/ml ethyl acetate *P. orientalis* (EAPO)**

**Lane 13: 50 mM with 0.125mg/ml ethyl acetate *P. orientalis* (EAPO)**

Cytoplasmic fraction

**245**

**180**

**140**

**100**

**75**

**60**

**45**

**35**

**25**

**20**

**15**

**10**

**5**

**1 2 3 4 5 6 7 8 9 10 11 12 13**


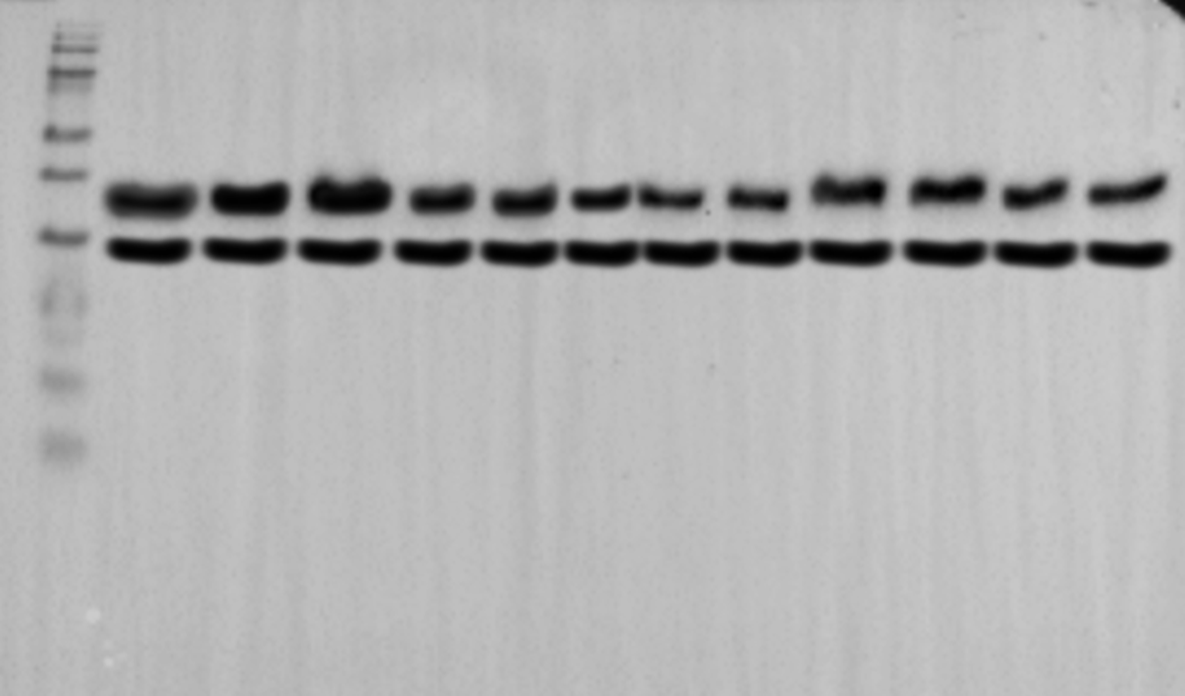


**Lane 1: ladder**

**Lane 2 5.5mM glucose**

**Lane 3: 25mM glucose**

**Lane 4: 50mM glucose**

**Lane 5: 5.5 mM with 0.03125mg/ml ethyl acetate *P. orientalis* (EAPO)**

**Lane 6: 5.5 mM with 0.0625mg/ml ethyl acetate *P. orientalis* (EAPO)**

**Lane 7: 5.5 mM with 0.125mg/ml ethyl acetate *P. orientalis* (EAPO)**

**Lane 8: 25 mM with 0.03125mg/ml ethyl acetate *P. orientalis* (EAPO)**

**Lane 9: 25 mM with 0.0625mg/ml ethyl acetate *P. orientalis* (EAPO)**

**Lane 10: 25 mM with 0.125mg/ml ethyl acetate *P. orientalis* (EAPO)**

**Lane 11: 50 mM with 0.03125mg/ml ethyl acetate *P. orientalis* (EAPO)**

**Lane 12: 50 mM with 0.0625mg/ml ethyl acetate *P. orientalis* (EAPO)**

**Lane 13: 50 mM with 0.125mg/ml ethyl acetate *P. orientalis* (EAPO)**

Phospho-MEK1/2– 43kDa

GAPDH-35kDa


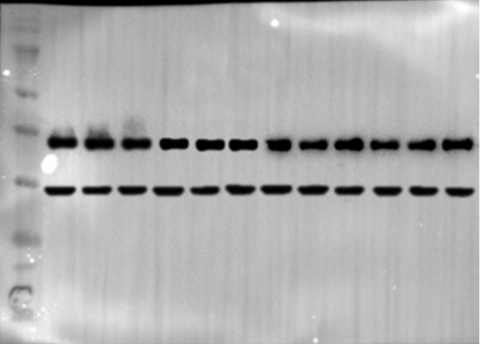


**245**

**180**

**140**

**100**

**75**

**60**

**45**

**35**

**25**

**20**

**15**

**10**

**5**

**1 2 3 4 5 6 7 8 9 10 11 12 13**

Nuclei fraction

**Lane 1: ladder**

**Lane 2 5.5mM glucose**

**Lane 3: 25mM glucose**

**Lane 4: 50mM glucose**

**Lane 5: 5.5 mM with 0.03125mg/ml ethyl acetate *P. orientalis* (EAPO)**

**Lane 6: 5.5 mM with 0.0625mg/ml ethyl acetate *P. orientalis* (EAPO)**

**Lane 7: 5.5 mM with 0.125mg/ml ethyl acetate *P. orientalis* (EAPO)**

**Lane 8: 25 mM with 0.03125mg/ml ethyl acetate *P. orientalis* (EAPO)**

**Lane 9: 25 mM with 0.0625mg/ml ethyl acetate *P. orientalis* (EAPO)**

**Lane 10: 25 mM with 0.125mg/ml ethyl acetate *P. orientalis* (EAPO)**

**Lane 11: 50 mM with 0.03125mg/ml ethyl acetate *P. orientalis* (EAPO)**

**Lane 12: 50 mM with 0.0625mg/ml ethyl acetate *P. orientalis* (EAPO)**

**Lane 13: 50 mM with 0.125mg/ml ethyl acetate *P. orientalis* (EAPO)**

Phospho-MEK1/2– 43kDa

GAPDH-35kDa

**Supplement 16.** Gel blot images for phospho-MEK1/2 (43 kDa) and GAPDH (36 kDa) expression in respective whole cell lysate, cytoplasmic, and nuclei fractions of retinal Müller cells. (Corresponds to Figure 7D)


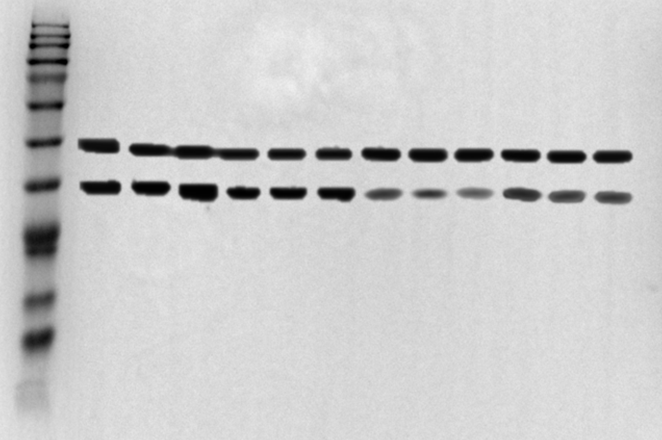
Whole cell lysate - AICAR treatment

**245**

**180**

**140**

**100**

**75**

**60**

**45**

**35**

**25**

**20**

**15**

**10**

**5**

**1 2 3 4 5 6 7 8 9 10 11 12 13**

**Lane 1: ladder**

**Lane 2 5.5mM glucose + AICAR**

**Lane 3: 25mM glucose+ AICAR**

**Lane 4: 50mM glucose+ AICAR**

**Lane 5: 5.5 mM with 0.03125mg/ml ethyl acetate *P. orientalis* (EAPO) +AICAR**

**Lane 6: 5.5 mM with 0.0625mg/ml ethyl acetate *P. orientalis* (EAPO) +AICAR**

**Lane 7: 5.5 mM with 0.125mg/ml ethyl acetate *P. orientalis* (EAPO) +AICAR**

**Lane 8: 25 mM with 0.03125mg/ml ethyl acetate *P. orientalis* (EAPO) +AICAR**

**Lane 9: 25 mM with 0.0625mg/ml ethyl acetate *P. orientalis* (EAPO) +AICAR**

**Lane 10: 25 mM with 0.125mg/ml ethyl acetate *P. orientalis* (EAPO) +AICAR**

**Lane 11: 50 mM with 0.03125mg/ml ethyl acetate *P. orientalis* (EAPO) +AICAR**

**Lane 12: 50 mM with 0.0625mg/ml ethyl acetate *P. orientalis* (EAPO) +AICAR**

**Lane 13: 50 mM with 0.125mg/ml ethyl acetate *P. orientalis* (EAPO) +AICAR**

Beta actin – 42kDa

VEGF-35kDa


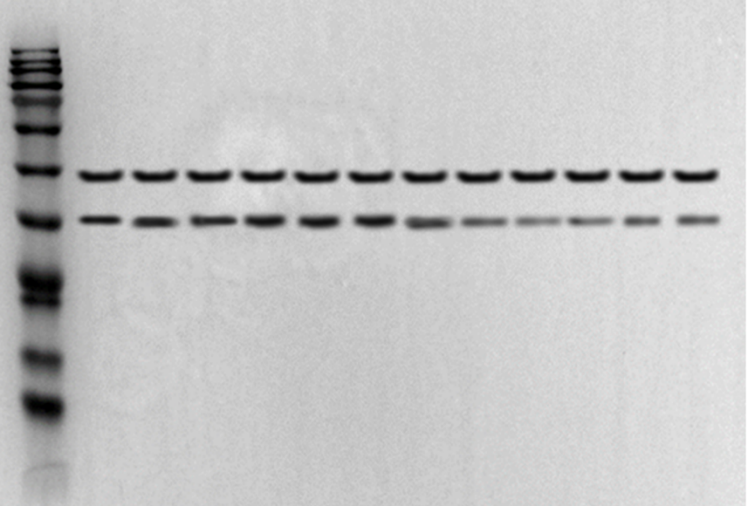
Whole cell lysate - compound C treatment

**1 2 3 4 5 6 7 8 9 10 11 12 13**

**245**

**180**

**140**

**100**

**75**

**60**

**45**

**35**

**25**

**20**

**15**

**10**

**5**

**Lane 1: ladder**

**Lane 2 5.5mM glucose +compound C**

**Lane 3: 25mM glucose+ compound C**

**Lane 4: 50mM glucose+ compound C**

**Lane 5: 5.5 mM with 0.03125mg/ml ethyl acetate *P. orientalis* (EAPO) + compound C**

**Lane 6: 5.5 mM with 0.0625mg/ml ethyl acetate *P. orientalis* (EAPO) + compound C**

**Lane 7: 5.5 mM with 0.125mg/ml ethyl acetate *P. orientalis* (EAPO) + compound C**

**Lane 8: 25 mM with 0.03125mg/ml ethyl acetate *P. orientalis* (EAPO) + compound C**

**Lane 9: 25 mM with 0.0625mg/ml ethyl acetate *P. orientalis* (EAPO) + compound C**

**Lane 10: 25 mM with 0.125mg/ml ethyl acetate *P. orientalis* (EAPO) + compound C**

**Lane 11: 50 mM with 0.03125mg/ml ethyl acetate *P. orientalis* (EAPO)+ compound C**

**Lane 12: 50 mM with 0.0625mg/ml ethyl acetate *P. orientalis* (EAPO) + compound C**

**Lane 13: 50 mM with 0.125mg/ml ethyl acetate *P. orientalis* (EAPO) + compound C**

Beta actin – 42kDa

VEGF-35kDa

**Supplement 17.** Gel blot images for VEGF (35 kDa) and beta actin (42 kDa) expression in the whole cell lysate of retinal Müller cells treated with AICAR or compound C. (Corresponds to Figure 8A and 8B)

Whole cell lysate – AICAR treatment


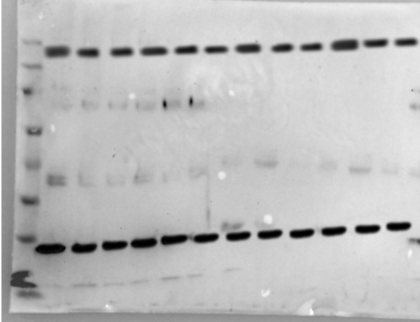


**1 2 3 4 5 6 7 8 9 10 11 12 13**

**245**

**180**

**140**

**100**

**75**

**60**

**45**

**35**

**25**

**20**

**15**

**10**

**5**

**Lane 1: ladder**

**Lane 2 5.5mM glucose +AICAR**

**Lane 3: 25mM glucose + AICAR**

**Lane 4: 50mM glucose + AICAR**

**Lane 5: 5.5 mM with 0.03125mg/ml ethyl acetate *P. orientalis* (EAPO) +AICAR**

**Lane 6: 5.5 mM with 0.0625mg/ml ethyl acetate *P. orientalis* (EAPO) +AICAR**

**Lane 7: 5.5 mM with 0.125mg/ml ethyl acetate *P. orientalis* (EAPO) +AICAR**

**Lane 8: 25 mM with 0.03125mg/ml ethyl acetate *P. orientalis* (EAPO) +AICAR**

**Lane 9: 25 mM with 0.0625mg/ml ethyl acetate *P. orientalis* (EAPO) +AICAR**

**Lane 10: 25 mM with 0.125mg/ml ethyl acetate *P. orientalis* (EAPO) +AICAR**

**Lane 11: 50 mM with 0.03125mg/ml ethyl acetate *P. orientalis* (EAPO) +AICAR**

**Lane 12: 50 mM with 0.0625mg/ml ethyl acetate *P. orientalis* (EAPO) +AICAR**

**Lane 13: 50 mM with 0.125mg/ml ethyl acetate *P. orientalis* (EAPO) +AICAR**

VEGFR2-210kDa

Beta actin – 42kDa


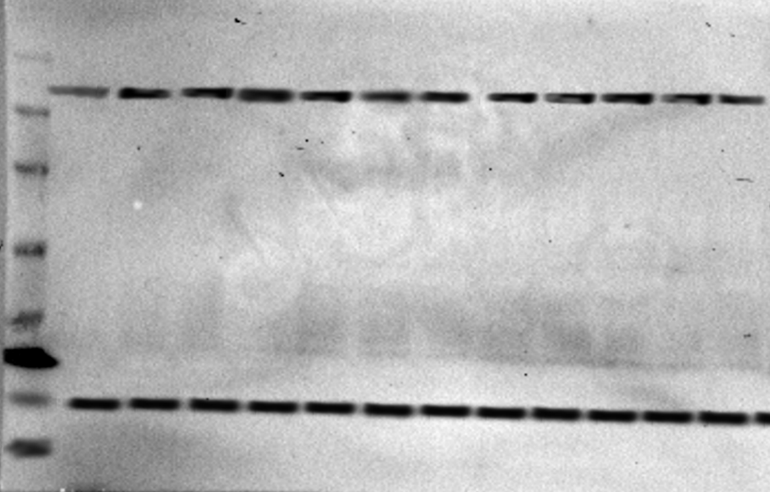
Whole cell lysate - compound C treatment

**245**

**180**

**140**

**100**

**75**

**60**

**45**

**35**

**1 2 3 4 5 6 7 8 9 10 11 12 13**

**Lane 1: ladder**

**Lane 2 5.5mM glucose +compound C**

**Lane 3: 25mM glucose+ compound C**

**Lane 4: 50mM glucose+ compound C**

**Lane 5: 5.5 mM with 0.03125mg/ml ethyl acetate *P. orientalis* (EAPO) + compound C**

**Lane 6: 5.5 mM with 0.0625mg/ml ethyl acetate *P. orientalis* (EAPO) + compound C**

**Lane 7: 5.5 mM with 0.125mg/ml ethyl acetate *P. orientalis* (EAPO) + compound C**

**Lane 8: 25 mM with 0.03125mg/ml ethyl acetate *P. orientalis* (EAPO) + compound C**

**Lane 9: 25 mM with 0.0625mg/ml ethyl acetate *P. orientalis* (EAPO) + compound C**

**Lane 10: 25 mM with 0.125mg/ml ethyl acetate *P. orientalis* (EAPO) + compound C**

**Lane 11: 50 mM with 0.03125mg/ml ethyl acetate *P. orientalis* (EAPO)+ compound C**

**Lane 12: 50 mM with 0.0625mg/ml ethyl acetate *P. orientalis* (EAPO) + compound C**

**Lane 13: 50 mM with 0.125mg/ml ethyl acetate *P. orientalis* (EAPO) + compound C**

VEGFR2-210kDa

Beta actin – 42kDa

**Supplement 18.** Gel blot images for VEGFR2 (210 kDa) and beta actin (42 kDa) expression in the whole cell lysate of retinal Müller cells treated with AICAR or compound C. (Corresponds to Figure 9A and 9B)


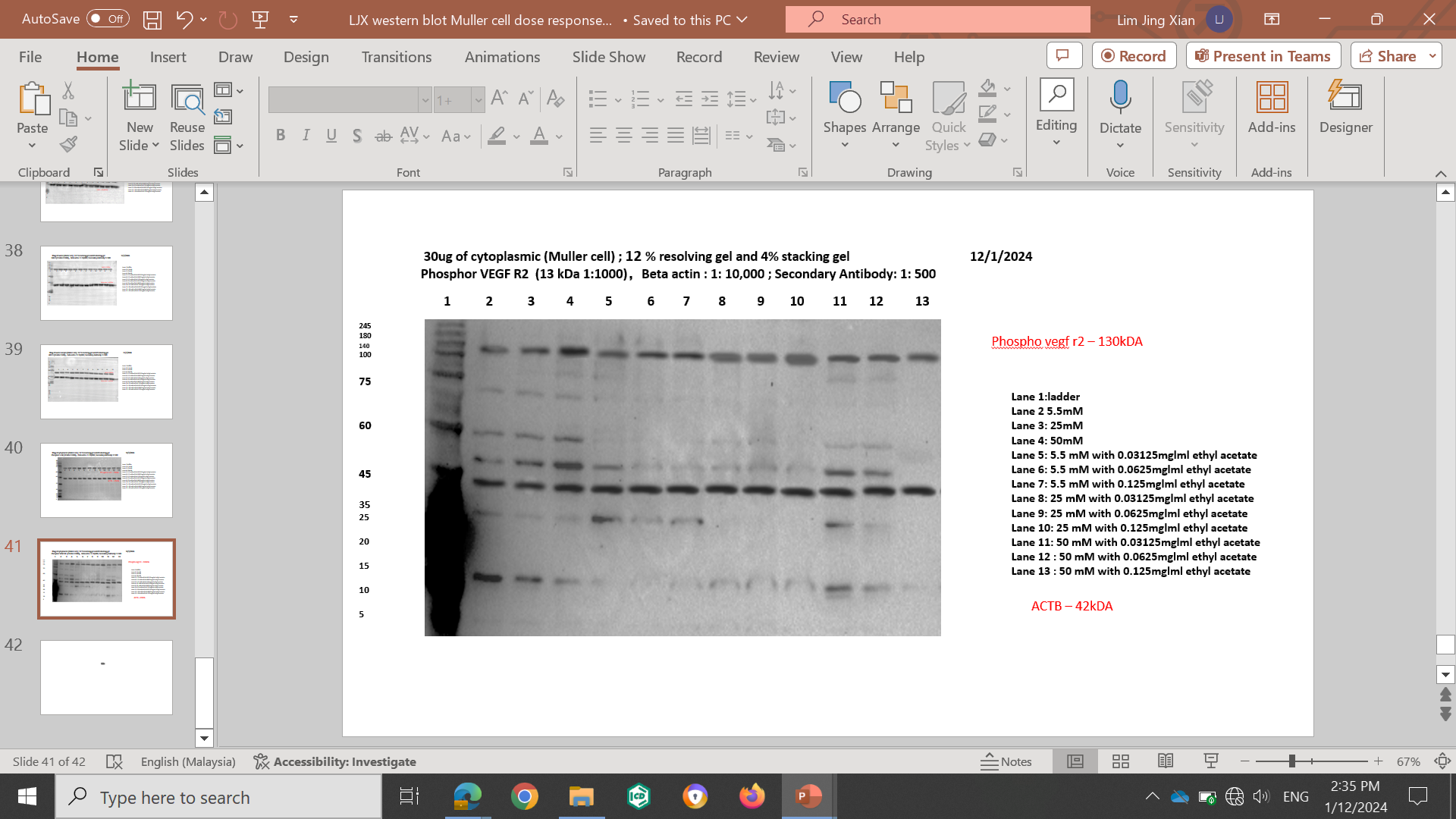
Whole cell lysate – AICAR treatment

**245**

**180**

**140**

**100**

**75**

**60**

**45**

**35**

**25**

**20**

**15**

**10**

**5**

**1 2 3 4 5 6 7 8 9 10 11 12 13**

**Lane 1: ladder**

**Lane 2 5.5mM glucose +AICAR**

**Lane 3: 25mM glucose + AICAR**

**Lane 4: 50mM glucose + AICAR**

**Lane 5: 5.5 mM with 0.03125mg/ml ethyl acetate *P. orientalis* (EAPO) +AICAR**

**Lane 6: 5.5 mM with 0.0625mg/ml ethyl acetate *P. orientalis* (EAPO) +AICAR**

**Lane 7: 5.5 mM with 0.125mg/ml ethyl acetate *P. orientalis* (EAPO) +AICAR**

**Lane 8: 25 mM with 0.03125mg/ml ethyl acetate *P. orientalis* (EAPO) +AICAR**

**Lane 9: 25 mM with 0.0625mg/ml ethyl acetate *P. orientalis* (EAPO) +AICAR**

**Lane 10: 25 mM with 0.125mg/ml ethyl acetate *P. orientalis* (EAPO) +AICAR**

**Lane 11: 50 mM with 0.03125mg/ml ethyl acetate *P. orientalis* (EAPO) +AICAR**

**Lane 12: 50 mM with 0.0625mg/ml ethyl acetate *P. orientalis* (EAPO) +AICAR**

**Lane 13: 50 mM with 0.125mg/ml ethyl acetate *P. orientalis* (EAPO) +AICAR**

Phospho-VEGFR2-130kDa

Beta actin – 42kDa

Whole cell lysate - compound C treatment

**1 2 3 4 5 6 7 8 9 10 11 12 13**

**245**

**180**

**140**

**100**

**75**

**60**

**45**

**35**

**25**

**20**

**15**

**10**

**5**

**Lane 1: ladder**

**Lane 2 5.5mM glucose +compound C**

**Lane 3: 25mM glucose+ compound C**

**Lane 4: 50mM glucose+ compound C**

**Lane 5: 5.5 mM with 0.03125mg/ml ethyl acetate *P. orientalis* (EAPO) + compound C**

**Lane 6: 5.5 mM with 0.0625mg/ml ethyl acetate *P. orientalis* (EAPO) + compound C**

**Lane 7: 5.5 mM with 0.125mg/ml ethyl acetate *P. orientalis* (EAPO) + compound C**

**Lane 8: 25 mM with 0.03125mg/ml ethyl acetate *P. orientalis* (EAPO) + compound C**

**Lane 9: 25 mM with 0.0625mg/ml ethyl acetate *P. orientalis* (EAPO) + compound C**

**Lane 10: 25 mM with 0.125mg/ml ethyl acetate *P. orientalis* (EAPO) + compound C**

**Lane 11: 50 mM with 0.03125mg/ml ethyl acetate *P. orientalis* (EAPO)+ compound C**

**Lane 12: 50 mM with 0.0625mg/ml ethyl acetate *P. orientalis* (EAPO) + compound C**

**Lane 13: 50 mM with 0.125mg/ml ethyl acetate *P. orientalis* (EAPO) + compound C**


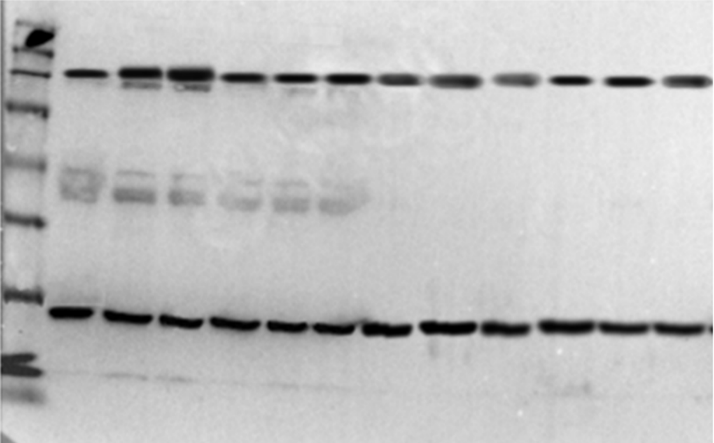


Beta actin – 42kDa

Phospho-VEGFR2-130kDa

**Supplement 19.** Gel blot images for phospho-VEGFR2 (130 kDa) and beta actin (42 kDa) expression in the whole cell lysate of retinal Müller cells treated with AICAR or compound C. (Corresponds to Figure 9C and 9D)

Whole cell lysate – AICAR treatment

**1 2 3 4 5 6 7 8 9 10 11 12 13**


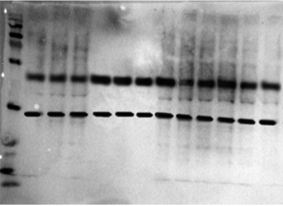


**245**

**180**

**140**

**100**

**75**

**60**

**45**

**35**

**25**

**20**

**15**

**10**

Beta actin – 42kDa

AKT1-56kDa

**Lane 1: ladder**

**Lane 2 5.5mM glucose +AICAR**

**Lane 3: 25mM glucose + AICAR**

**Lane 4: 50mM glucose + AICAR**

**Lane 5: 5.5 mM with 0.03125mg/ml ethyl acetate *P. orientalis* (EAPO) +AICAR**

**Lane 6: 5.5 mM with 0.0625mg/ml ethyl acetate *P. orientalis* (EAPO) +AICAR**

**Lane 7: 5.5 mM with 0.125mg/ml ethyl acetate *P. orientalis* (EAPO) +AICAR**

**Lane 8: 25 mM with 0.03125mg/ml ethyl acetate *P. orientalis* (EAPO) +AICAR**

**Lane 9: 25 mM with 0.0625mg/ml ethyl acetate *P. orientalis* (EAPO) +AICAR**

**Lane 10: 25 mM with 0.125mg/ml ethyl acetate *P. orientalis* (EAPO) +AICAR**

**Lane 11: 50 mM with 0.03125mg/ml ethyl acetate *P. orientalis* (EAPO) +AICAR**

**Lane 12: 50 mM with 0.0625mg/ml ethyl acetate *P. orientalis* (EAPO) +AICAR**

**Lane 13: 50 mM with 0.125mg/ml ethyl acetate *P. orientalis* (EAPO) +AICAR**

Whole cell lysate - compound C treatment

**1 2 3 4 5 6 7 8 9 10 11 12 13**


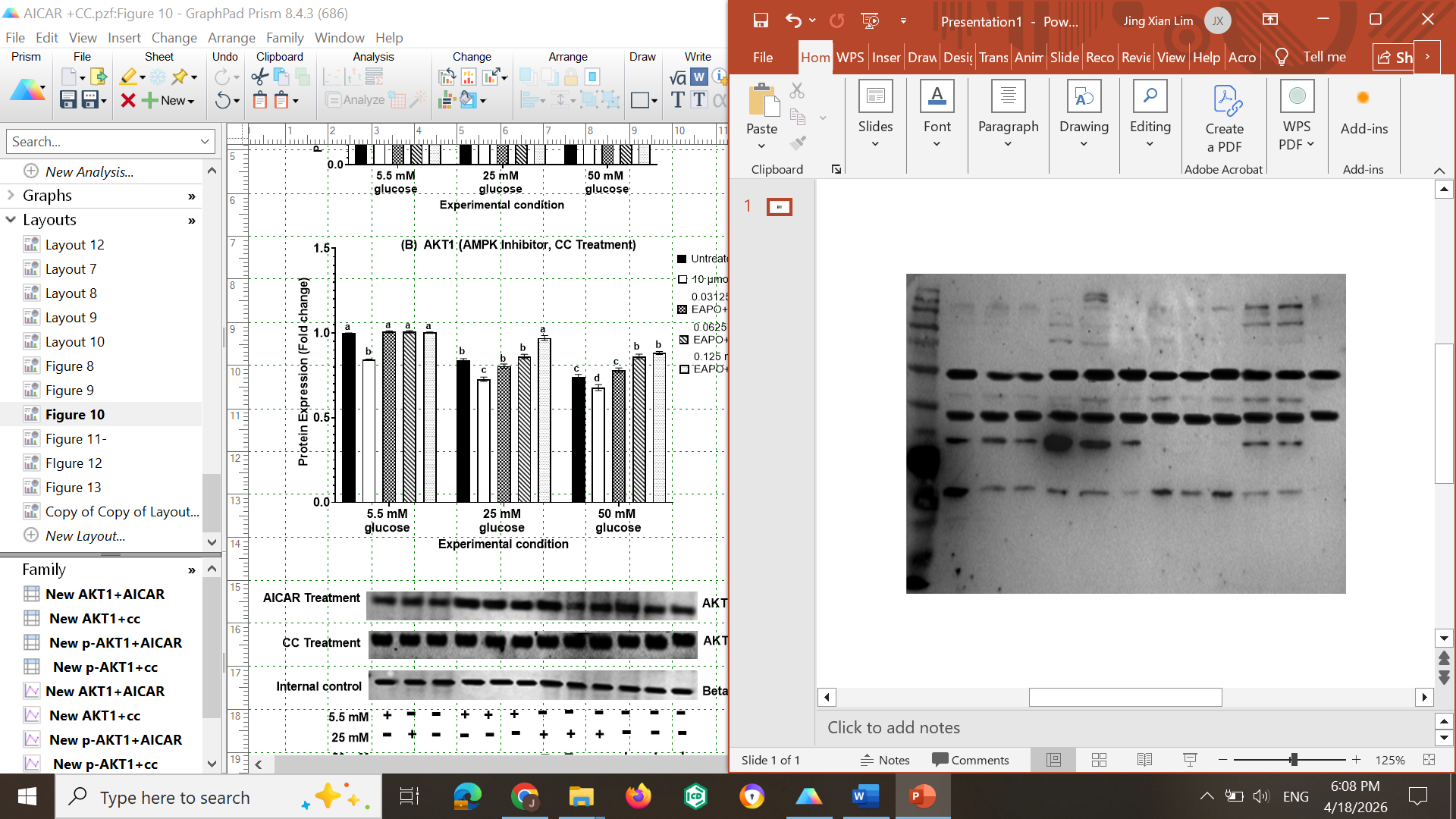


**245**

**180**

**140**

**100**

**75**

**60**

**45**

**35**

**25**

**20**

**15**

**10**

**Lane 1: ladder**

**Lane 2 5.5mM glucose +compound C**

**Lane 3: 25mM glucose+ compound C**

**Lane 4: 50mM glucose+ compound C**

**Lane 5: 5.5 mM with 0.03125mg/ml ethyl acetate *P. orientalis* (EAPO) + compound C**

**Lane 6: 5.5 mM with 0.0625mg/ml ethyl acetate *P. orientalis* (EAPO) + compound C**

**Lane 7: 5.5 mM with 0.125mg/ml ethyl acetate *P. orientalis* (EAPO) + compound C**

**Lane 8: 25 mM with 0.03125mg/ml ethyl acetate *P. orientalis* (EAPO) + compound C**

**Lane 9: 25 mM with 0.0625mg/ml ethyl acetate *P. orientalis* (EAPO) + compound C**

**Lane 10: 25 mM with 0.125mg/ml ethyl acetate *P. orientalis* (EAPO) + compound C**

**Lane 11: 50 mM with 0.03125mg/ml ethyl acetate *P. orientalis* (EAPO)+ compound C**

**Lane 12: 50 mM with 0.0625mg/ml ethyl acetate *P. orientalis* (EAPO) + compound C**

**Lane 13: 50 mM with 0.125mg/ml ethyl acetate *P. orientalis* (EAPO) + compound C**

AKT1-56kDa

Beta actin – 42kDa

**Supplement 20.** Gel blot images for AKT1 (56 kDa) and beta actin (42 kDa) expression in the whole cell lysate of retinal Müller cells treated with AICAR or compound C. (Corresponds to Figure 10A and 10B)

Whole cell lysate – AICAR treatment

**245**

**180**

**140**

**100**

**75**

**60**

**45**

**35**

**25**

**20**

**15**

**10**

**1 2 3 4 5 6 7 8 9 10 11 12 13**


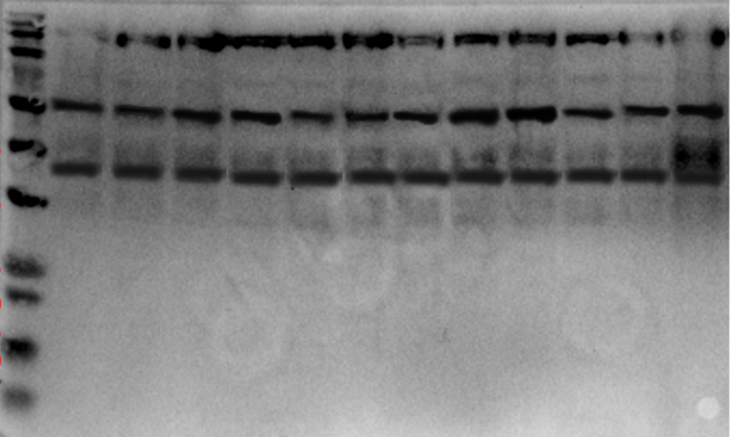


Phospho-AKT1-55kDa

Beta actin – 42kDa

**Lane 1: ladder**

**Lane 2 5.5mM glucose +AICAR**

**Lane 3: 25mM glucose + AICAR**

**Lane 4: 50mM glucose + AICAR**

**Lane 5: 5.5 mM with 0.03125mg/ml ethyl acetate *P. orientalis* (EAPO) +AICAR**

**Lane 6: 5.5 mM with 0.0625mg/ml ethyl acetate *P. orientalis* (EAPO) +AICAR**

**Lane 7: 5.5 mM with 0.125mg/ml ethyl acetate *P. orientalis* (EAPO) +AICAR**

**Lane 8: 25 mM with 0.03125mg/ml ethyl acetate *P. orientalis* (EAPO) +AICAR**

**Lane 9: 25 mM with 0.0625mg/ml ethyl acetate *P. orientalis* (EAPO) +AICAR**

**Lane 10: 25 mM with 0.125mg/ml ethyl acetate *P. orientalis* (EAPO) +AICAR**

**Lane 11: 50 mM with 0.03125mg/ml ethyl acetate *P. orientalis* (EAPO) +AICAR**

**Lane 12: 50 mM with 0.0625mg/ml ethyl acetate *P. orientalis* (EAPO) +AICAR**

**Lane 13: 50 mM with 0.125mg/ml ethyl acetate *P. orientalis* (EAPO) +AICAR**

Whole cell lysate - compound C treatment


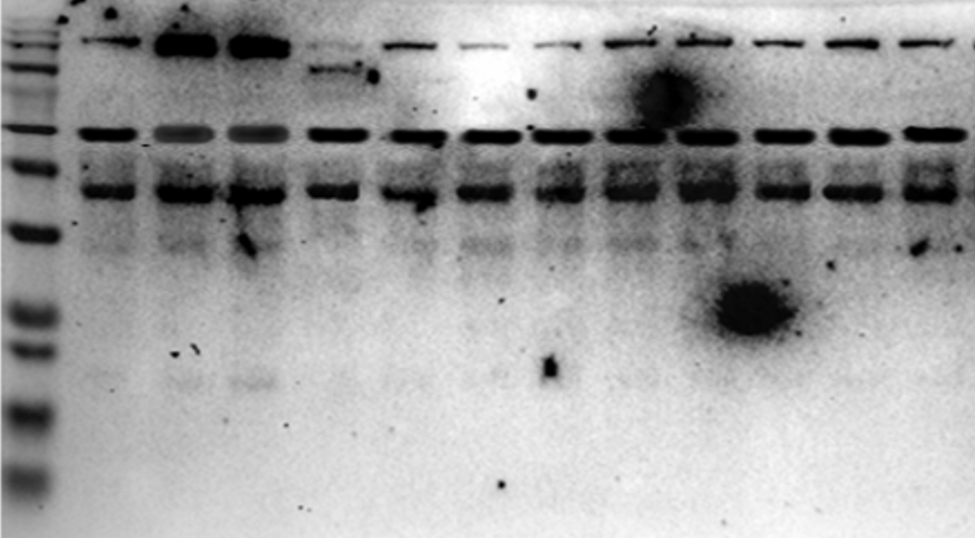


**1 2 3 4 5 6 7 8 9 10 11 12 13**

**245**

**180**

**140**

**100**

**75**

**60**

**45**

**35**

**25**

**20**

**15**

**10**

**5**

**Lane 1: ladder**

**Lane 2 5.5mM glucose +compound C**

**Lane 3: 25mM glucose+ compound C**

**Lane 4: 50mM glucose+ compound C**

**Lane 5: 5.5 mM with 0.03125mg/ml ethyl acetate *P. orientalis* (EAPO) + compound C**

**Lane 6: 5.5 mM with 0.0625mg/ml ethyl acetate *P. orientalis* (EAPO) + compound C**

**Lane 7: 5.5 mM with 0.125mg/ml ethyl acetate *P. orientalis* (EAPO) + compound C**

**Lane 8: 25 mM with 0.03125mg/ml ethyl acetate *P. orientalis* (EAPO) + compound C**

**Lane 9: 25 mM with 0.0625mg/ml ethyl acetate *P. orientalis* (EAPO) + compound C**

**Lane 10: 25 mM with 0.125mg/ml ethyl acetate *P. orientalis* (EAPO) + compound C**

**Lane 11: 50 mM with 0.03125mg/ml ethyl acetate *P. orientalis* (EAPO)+ compound C**

**Lane 12: 50 mM with 0.0625mg/ml ethyl acetate *P. orientalis* (EAPO) + compound C**

**Lane 13: 50 mM with 0.125mg/ml ethyl acetate *P. orientalis* (EAPO) + compound C**

Phospho-AKT1-55kDa

Beta actin – 42kDa

**Supplement 21.** Gel blot images for phospho-AKT1 (55 kDa) and beta actin (42 kDa) expression in the whole cell lysate of retinal Müller cells treated with AICAR or compound C. (Corresponds to Figure 10C and 10D)

Whole cell lysate – AICAR treatment

**1 2 3 4 5 6 7 8 9 10 11 12 13**


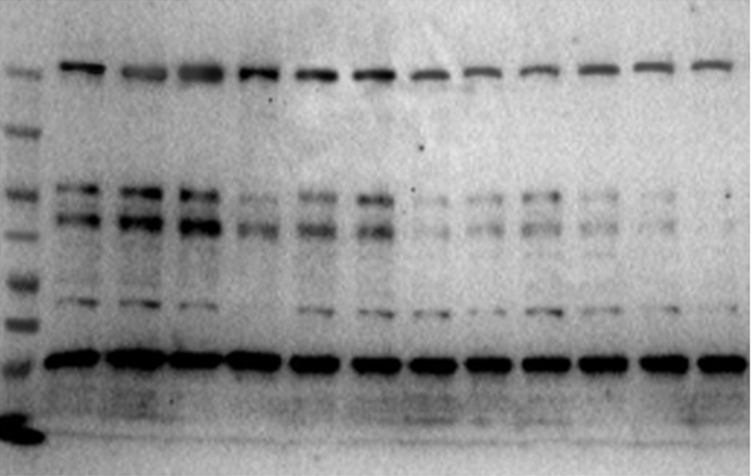


**245**

**180**

**140**

**100**

**75**

**60**

**45**

**35**

**25**

**20**

**15**

**10**

**5**

**Lane 1: ladder**

**Lane 2 5.5mM glucose +AICAR**

**Lane 3: 25mM glucose + AICAR**

**Lane 4: 50mM glucose + AICAR**

**Lane 5: 5.5 mM with 0.03125mg/ml ethyl acetate *P. orientalis* (EAPO) +AICAR**

**Lane 6: 5.5 mM with 0.0625mg/ml ethyl acetate *P. orientalis* (EAPO) +AICAR**

**Lane 7: 5.5 mM with 0.125mg/ml ethyl acetate *P. orientalis* (EAPO) +AICAR**

**Lane 8: 25 mM with 0.03125mg/ml ethyl acetate *P. orientalis* (EAPO) +AICAR**

**Lane 9: 25 mM with 0.0625mg/ml ethyl acetate *P. orientalis* (EAPO) +AICAR**

**Lane 10: 25 mM with 0.125mg/ml ethyl acetate *P. orientalis* (EAPO) +AICAR**

**Lane 11: 50 mM with 0.03125mg/ml ethyl acetate *P. orientalis* (EAPO) +AICAR**

**Lane 12: 50 mM with 0.0625mg/ml ethyl acetate *P. orientalis* (EAPO) +AICAR**

**Lane 13: 50 mM with 0.125mg/ml ethyl acetate *P. orientalis* (EAPO) +AICAR**

mTOR-289kDa

Beta actin – 42kDa

Whole cell lysate - compound C treatment

**1 2 3 4 5 6 7 8 9 10 11 12 13**


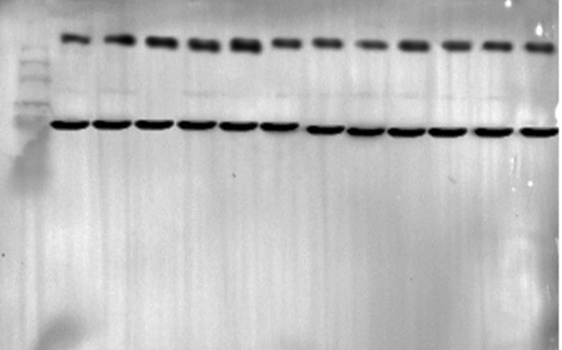


**245**

**180**

**140**

**100**

**75**

**60**

**45**

**35**

**25**

**20**

Beta actin – 42kDa

mTOR-289kDa

**Lane 1: ladder**

**Lane 2 5.5mM glucose +compound C**

**Lane 3: 25mM glucose+ compound C**

**Lane 4: 50mM glucose+ compound C**

**Lane 5: 5.5 mM with 0.03125mg/ml ethyl acetate *P. orientalis* (EAPO) + compound C**

**Lane 6: 5.5 mM with 0.0625mg/ml ethyl acetate *P. orientalis* (EAPO) + compound C**

**Lane 7: 5.5 mM with 0.125mg/ml ethyl acetate *P. orientalis* (EAPO) + compound C**

**Lane 8: 25 mM with 0.03125mg/ml ethyl acetate *P. orientalis* (EAPO) + compound C**

**Lane 9: 25 mM with 0.0625mg/ml ethyl acetate *P. orientalis* (EAPO) + compound C**

**Lane 10: 25 mM with 0.125mg/ml ethyl acetate *P. orientalis* (EAPO) + compound C**

**Lane 11: 50 mM with 0.03125mg/ml ethyl acetate *P. orientalis* (EAPO)+ compound C**

**Lane 12: 50 mM with 0.0625mg/ml ethyl acetate *P. orientalis* (EAPO) + compound C**

**Lane 13: 50 mM with 0.125mg/ml ethyl acetate *P. orientalis* (EAPO) + compound C**

**Supplement 22.** Gel blot images for mTOR (289 kDa) and beta actin (42 kDa) expression in the whole cell lysate of retinal Müller cells treated with AICAR or compound C. (Corresponds to Figure 11A and 11B)

Whole cell lysate -AICAR treatment

**1 2 3 4 5 6 7 8 9 10 11 12 13**


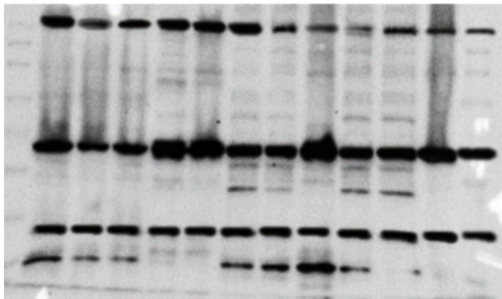


**245**

**180**

**140**

**100**

**75**

**60**

**45**

**35**

**25**

**20**

**15**

**10**

**5**

Phospho-mTOR-288kDa

Beta actin – 42kDa

**Lane 1: ladder**

**Lane 2 5.5mM glucose +AICAR**

**Lane 3: 25mM glucose + AICAR**

**Lane 4: 50mM glucose + AICAR**

**Lane 5: 5.5 mM with 0.03125mg/ml ethyl acetate *P. orientalis* (EAPO) +AICAR**

**Lane 6: 5.5 mM with 0.0625mg/ml ethyl acetate *P. orientalis* (EAPO) +AICAR**

**Lane 7: 5.5 mM with 0.125mg/ml ethyl acetate *P. orientalis* (EAPO) +AICAR**

**Lane 8: 25 mM with 0.03125mg/ml ethyl acetate *P. orientalis* (EAPO) +AICAR**

**Lane 9: 25 mM with 0.0625mg/ml ethyl acetate *P. orientalis* (EAPO) +AICAR**

**Lane 10: 25 mM with 0.125mg/ml ethyl acetate *P. orientalis* (EAPO) +AICAR**

**Lane 11: 50 mM with 0.03125mg/ml ethyl acetate *P. orientalis* (EAPO) +AICAR**

**Lane 12: 50 mM with 0.0625mg/ml ethyl acetate *P. orientalis* (EAPO) +AICAR**

**Lane 13: 50 mM with 0.125mg/ml ethyl acetate *P. orientalis* (EAPO) +AICAR**


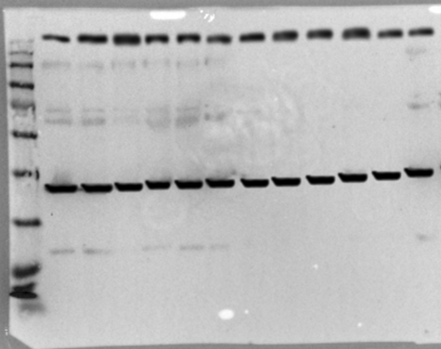
Whole cell lysate- compound C treatment

**1 2 3 4 5 6 7 8 9 10 11 12 13**

Phospho-mTOR-288kDa

Beta actin – 42kDa

**Lane 1: ladder**

**Lane 2 5.5mM glucose +compound C**

**Lane 3: 25mM glucose+ compound C**

**Lane 4: 50mM glucose+ compound C**

**Lane 5: 5.5 mM with 0.03125mg/ml ethyl acetate *P. orientalis* (EAPO) + compound C**

**Lane 6: 5.5 mM with 0.0625mg/ml ethyl acetate *P. orientalis* (EAPO) + compound C**

**Lane 7: 5.5 mM with 0.125mg/ml ethyl acetate *P. orientalis* (EAPO) + compound C**

**Lane 8: 25 mM with 0.03125mg/ml ethyl acetate *P. orientalis* (EAPO) + compound C**

**Lane 9: 25 mM with 0.0625mg/ml ethyl acetate *P. orientalis* (EAPO) + compound C**

**Lane 10: 25 mM with 0.125mg/ml ethyl acetate *P. orientalis* (EAPO) + compound C**

**Lane 11: 50 mM with 0.03125mg/ml ethyl acetate *P. orientalis* (EAPO)+ compound C**

**Lane 12: 50 mM with 0.0625mg/ml ethyl acetate *P. orientalis* (EAPO) + compound C**

**Lane 13: 50 mM with 0.125mg/ml ethyl acetate *P. orientalis* (EAPO) + compound C**

**245**

**180**

**140**

**100**

**75**

**60**

**45**

**35**

**25**

**20**

**15**

**Supplement 23.** Gel blot images for phospho-mTOR (288 kDa) and beta actin (42 kDa) expression in the whole cell lysate of retinal Müller cells treated with AICAR or compound C. (Corresponds to Figure 11C and 11D)


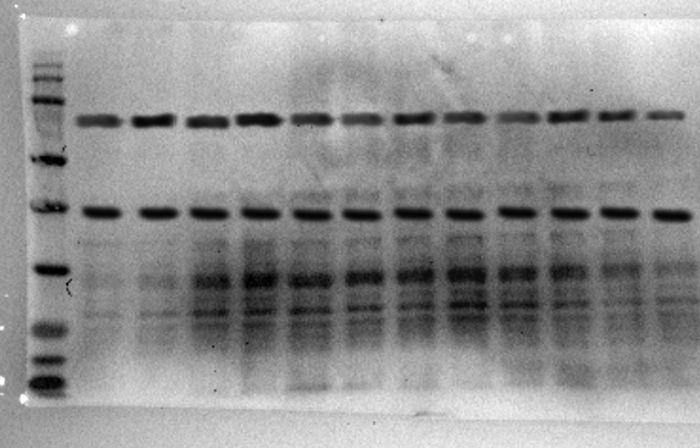
Whole cell lysate – AICAR treatment

**Lane 1: ladder**

**Lane 2 5.5mM glucose +AICAR**

**Lane 3: 25mM glucose + AICAR**

**Lane 4: 50mM glucose + AICAR**

**Lane 5: 5.5 mM with 0.03125mg/ml ethyl acetate *P. orientalis* (EAPO) +AICAR**

**Lane 6: 5.5 mM with 0.0625mg/ml ethyl acetate *P. orientalis* (EAPO) +AICAR**

**Lane 7: 5.5 mM with 0.125mg/ml ethyl acetate *P. orientalis* (EAPO) +AICAR**

**Lane 8: 25 mM with 0.03125mg/ml ethyl acetate *P. orientalis* (EAPO) +AICAR**

**Lane 9: 25 mM with 0.0625mg/ml ethyl acetate *P. orientalis* (EAPO) +AICAR**

**Lane 10: 25 mM with 0.125mg/ml ethyl acetate *P. orientalis* (EAPO) +AICAR**

**Lane 11: 50 mM with 0.03125mg/ml ethyl acetate *P. orientalis* (EAPO) +AICAR**

**Lane 12: 50 mM with 0.0625mg/ml ethyl acetate *P. orientalis* (EAPO) +AICAR**

**Lane 13: 50 mM with 0.125mg/ml ethyl acetate *P. orientalis* (EAPO) +AICAR**

**1 2 3 4 5 6 7 8 9 10 11 12 13**

**245**

**180**

**140**

**100**

**75**

**60**

**45**

**35**

**25**

**20**

**15**

**10**

**5**

Raf-1-75kDa

Beta actin – 42kDa

Whole cell lysate - compound C treatment

**1 2 3 4 5 6 7 8 9 10 11 12 13**


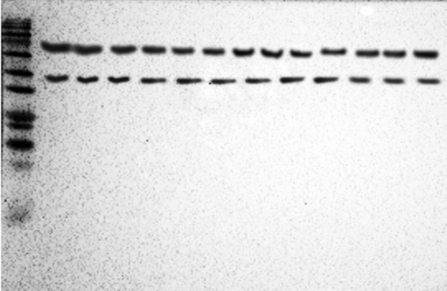


**Lane 1: ladder**

**Lane 2 5.5mM glucose +compound C**

**Lane 3: 25mM glucose+ compound C**

**Lane 4: 50mM glucose+ compound C**

**Lane 5: 5.5 mM with 0.03125mg/ml ethyl acetate *P. orientalis* (EAPO) + compound C**

**Lane 6: 5.5 mM with 0.0625mg/ml ethyl acetate *P. orientalis* (EAPO) + compound C**

**Lane 7: 5.5 mM with 0.125mg/ml ethyl acetate *P. orientalis* (EAPO) + compound C**

**Lane 8: 25 mM with 0.03125mg/ml ethyl acetate *P. orientalis* (EAPO) + compound C**

**Lane 9: 25 mM with 0.0625mg/ml ethyl acetate *P. orientalis* (EAPO) + compound C**

**Lane 10: 25 mM with 0.125mg/ml ethyl acetate *P. orientalis* (EAPO) + compound C**

**Lane 11: 50 mM with 0.03125mg/ml ethyl acetate *P. orientalis* (EAPO)+ compound C**

**Lane 12: 50 mM with 0.0625mg/ml ethyl acetate *P. orientalis* (EAPO) + compound C**

**Lane 13: 50 mM with 0.125mg/ml ethyl acetate *P. orientalis* (EAPO) + compound C**

**245**

**180**

**140**

**100**

**75**

**60**

**45**

**35**

**25**

**20**

**15**

**10**

**5**

Raf-1-75kDa

Beta actin – 42kDa

**Supplement 24.** Gel blot images for Raf-1 (75 kDa) and beta actin (42 kDa) expression in the whole cell lysate of retinal Müller cells treated with AICAR or compound C. (Corresponds to Figure 12A and 12B)

Whole cell lysate - AICAR treatment

**245**

**180**

**140**

**100**

**75**

**60**

**45**

**35**

**25**

**20**

**15**

**10**

**5**

**1 2 3 4 5 6 7 8 9 10 11 12 13**


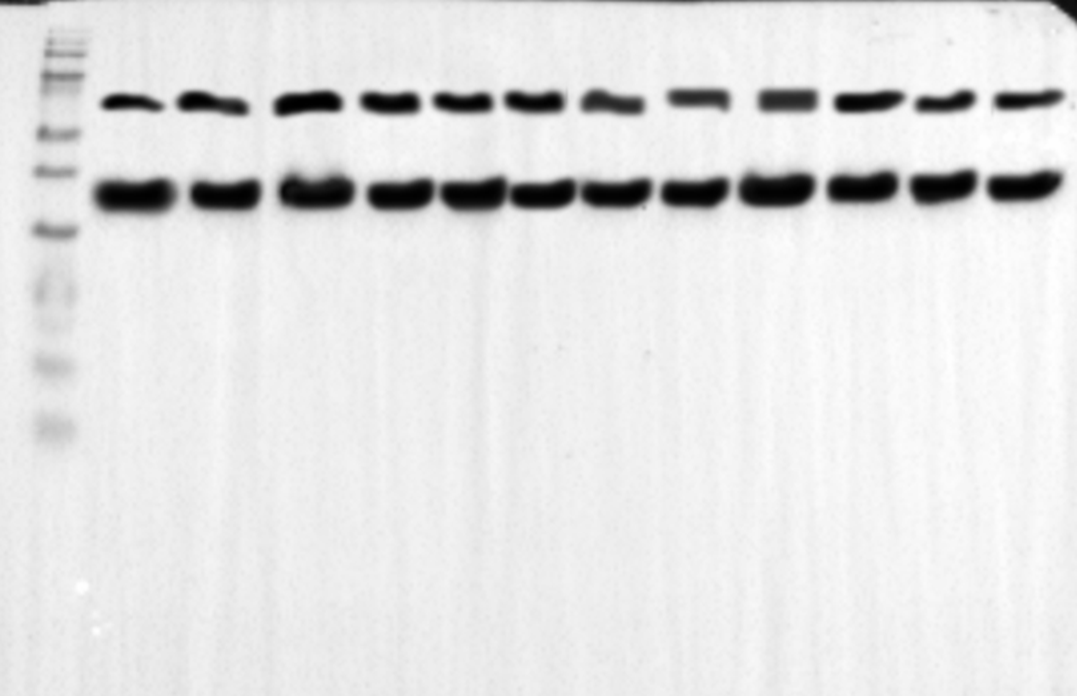


**Lane 1: ladder**

**Lane 2 5.5mM glucose +AICAR**

**Lane 3: 25mM glucose + AICAR**

**Lane 4: 50mM glucose + AICAR**

**Lane 5: 5.5 mM with 0.03125mg/ml ethyl acetate *P. orientalis* (EAPO) +AICAR**

**Lane 6: 5.5 mM with 0.0625mg/ml ethyl acetate *P. orientalis* (EAPO) +AICAR**

**Lane 7: 5.5 mM with 0.125mg/ml ethyl acetate *P. orientalis* (EAPO) +AICAR**

**Lane 8: 25 mM with 0.03125mg/ml ethyl acetate *P. orientalis* (EAPO) +AICAR**

**Lane 9: 25 mM with 0.0625mg/ml ethyl acetate *P. orientalis* (EAPO) +AICAR**

**Lane 10: 25 mM with 0.125mg/ml ethyl acetate *P. orientalis* (EAPO) +AICAR**

**Lane 11: 50 mM with 0.03125mg/ml ethyl acetate *P. orientalis* (EAPO) +AICAR**

**Lane 12: 50 mM with 0.0625mg/ml ethyl acetate *P. orientalis* (EAPO) +AICAR**

**Lane 13: 50 mM with 0.125mg/ml ethyl acetate *P. orientalis* (EAPO) +AICAR**

Phospho-Raf-1-72kDa

Beta actin – 42kDa

Whole cell lysate - compound C treatment

**1 2 3 4 5 6 7 8 9 10 11 12 13**


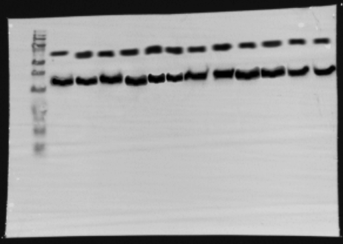


**245**

**180**

**140**

**100**

**75**

**60**

**45**

**35**

**25**

**20**

**15**

**10**

**5**

**Lane 1: ladder**

**Lane 2 5.5mM glucose +compound C**

**Lane 3: 25mM glucose+ compound C**

**Lane 4: 50mM glucose+ compound C**

**Lane 5: 5.5 mM with 0.03125mg/ml ethyl acetate *P. orientalis* (EAPO) + compound C**

**Lane 6: 5.5 mM with 0.0625mg/ml ethyl acetate *P. orientalis* (EAPO) + compound C**

**Lane 7: 5.5 mM with 0.125mg/ml ethyl acetate *P. orientalis* (EAPO) + compound C**

**Lane 8: 25 mM with 0.03125mg/ml ethyl acetate *P. orientalis* (EAPO) + compound C**

**Lane 9: 25 mM with 0.0625mg/ml ethyl acetate *P. orientalis* (EAPO) + compound C**

**Lane 10: 25 mM with 0.125mg/ml ethyl acetate *P. orientalis* (EAPO) + compound C**

**Lane 11: 50 mM with 0.03125mg/ml ethyl acetate *P. orientalis* (EAPO)+ compound C**

**Lane 12: 50 mM with 0.0625mg/ml ethyl acetate *P. orientalis* (EAPO) + compound C**

**Lane 13: 50 mM with 0.125mg/ml ethyl acetate *P. orientalis* (EAPO) + compound C**

Phospho-Raf-1-72kDa

Beta actin – 42kDa

**Supplement 25.** Gel blot images for phospho-Raf-1 (72 kDa) and beta actin (42 kDa) expression in the whole cell lysate of retinal Müller cells treated with AICAR or compound C. (Corresponds to Figure 12C and 12D).

Whole cell lysate-AICAR treatment


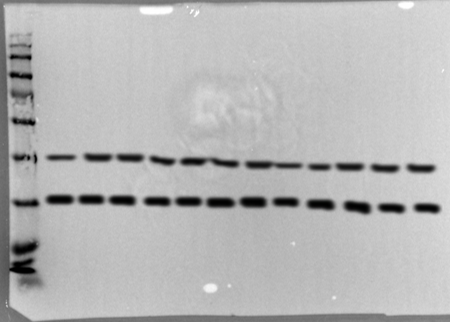


**1 2 3 4 5 6 7 8 9 10 11 12 13**

**245**

**180**

**140**

**100**

**75**

**60**

**45**

**35**

**25**

**20**

**15**

**10**

**Lane 1: ladder**

**Lane 2 5.5mM glucose +AICAR**

**Lane 3: 25mM glucose + AICAR**

**Lane 4: 50mM glucose + AICAR**

**Lane 5: 5.5 mM with 0.03125mg/ml ethyl acetate *P. orientalis* (EAPO) +AICAR**

**Lane 6: 5.5 mM with 0.0625mg/ml ethyl acetate *P. orientalis* (EAPO) +AICAR**

**Lane 7: 5.5 mM with 0.125mg/ml ethyl acetate *P. orientalis* (EAPO) +AICAR**

**Lane 8: 25 mM with 0.03125mg/ml ethyl acetate *P. orientalis* (EAPO) +AICAR**

**Lane 9: 25 mM with 0.0625mg/ml ethyl acetate *P. orientalis* (EAPO) +AICAR**

**Lane 10: 25 mM with 0.125mg/ml ethyl acetate *P. orientalis* (EAPO) +AICAR**

**Lane 11: 50 mM with 0.03125mg/ml ethyl acetate *P. orientalis* (EAPO) +AICAR**

**Lane 12: 50 mM with 0.0625mg/ml ethyl acetate *P. orientalis* (EAPO) +AICAR**

**Lane 13: 50 mM with 0.125mg/ml ethyl acetate *P. orientalis* (EAPO) +AICAR**

MEK1/2– 45kDa

GAPDH – 36kDa

Whole cell lysate - compound C treatment


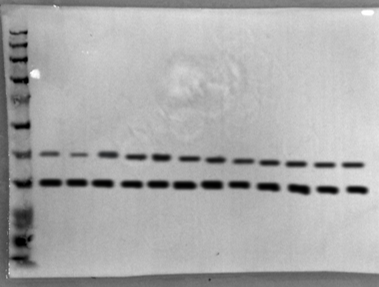


**1 2 3 4 5 6 7 8 9 10 11 12 13**

**245**

**180**

**140**

**100**

**75**

**60**

**45**

**35**

**25**

**20**

**15**

**10**

**5**

**Lane 1: ladder**

**Lane 2 5.5mM glucose +compound C**

**Lane 3: 25mM glucose+ compound C**

**Lane 4: 50mM glucose+ compound C**

**Lane 5: 5.5 mM with 0.03125mg/ml ethyl acetate *P. orientalis* (EAPO) + compound C**

**Lane 6: 5.5 mM with 0.0625mg/ml ethyl acetate *P. orientalis* (EAPO) + compound C**

**Lane 7: 5.5 mM with 0.125mg/ml ethyl acetate *P. orientalis* (EAPO) + compound C**

**Lane 8: 25 mM with 0.03125mg/ml ethyl acetate *P. orientalis* (EAPO) + compound C**

**Lane 9: 25 mM with 0.0625mg/ml ethyl acetate *P. orientalis* (EAPO) + compound C**

**Lane 10: 25 mM with 0.125mg/ml ethyl acetate *P. orientalis* (EAPO) + compound C**

**Lane 11: 50 mM with 0.03125mg/ml ethyl acetate *P. orientalis* (EAPO)+ compound C**

**Lane 12: 50 mM with 0.0625mg/ml ethyl acetate *P. orientalis* (EAPO) + compound C**

**Lane 13: 50 mM with 0.125mg/ml ethyl acetate *P. orientalis* (EAPO) + compound C**

MEK1/2– 45kDa

GAPDH – 36kDa

**Supplement 26.** Gel blot images for MEK1/2 (45 kDa) and GAPDH (36 kDa) expression in the whole cell lysate of retinal Müller cells treated with AICAR or compound C. (Corresponds to Figure 13A and 13B)

Whole cell lysate - AICAR treatment

**Lane 1: ladder**

**Lane 2 5.5mM glucose +AICAR**

**Lane 3: 25mM glucose + AICAR**

**Lane 4: 50mM glucose + AICAR**

**Lane 5: 5.5 mM with 0.03125mg/ml ethyl acetate *P. orientalis* (EAPO) +AICAR**

**Lane 6: 5.5 mM with 0.0625mg/ml ethyl acetate *P. orientalis* (EAPO) +AICAR**

**Lane 7: 5.5 mM with 0.125mg/ml ethyl acetate *P. orientalis* (EAPO) +AICAR**

**Lane 8: 25 mM with 0.03125mg/ml ethyl acetate *P. orientalis* (EAPO) +AICAR**

**Lane 9: 25 mM with 0.0625mg/ml ethyl acetate *P. orientalis* (EAPO) +AICAR**

**Lane 10: 25 mM with 0.125mg/ml ethyl acetate *P. orientalis* (EAPO) +AICAR**

**Lane 11: 50 mM with 0.03125mg/ml ethyl acetate *P. orientalis* (EAPO) +AICAR**

**Lane 12: 50 mM with 0.0625mg/ml ethyl acetate *P. orientalis* (EAPO) +AICAR**

**Lane 13: 50 mM with 0.125mg/ml ethyl acetate *P. orientalis* (EAPO) +AICAR**

**245**

**180**

**140**

**100**

**75**

**60**

**45**

**35**

**25**

**20**

**1 2 3 4 5 6 7 8 9 10 11 12 13**


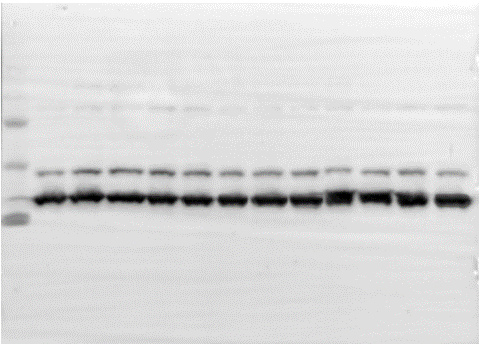


Phospho-MEK1/2– 43kDa

GAPDH – 36kDa

Whole cell lysate - compound C treatment

**245**

**180**

**140**

**100**

**75**

**60**

**45**

**35**

**25**

**20**

**15**

**1 2 3 4 5 6 7 8 9 10 11 12 13**

**Lane 1: ladder**

**Lane 2 5.5mM glucose +compound C**

**Lane 3: 25mM glucose+ compound C**

**Lane 4: 50mM glucose+ compound C**

**Lane 5: 5.5 mM with 0.03125mg/ml ethyl acetate *P. orientalis* (EAPO) + compound C**

**Lane 6: 5.5 mM with 0.0625mg/ml ethyl acetate *P. orientalis* (EAPO) + compound C**

**Lane 7: 5.5 mM with 0.125mg/ml ethyl acetate *P. orientalis* (EAPO) + compound C**

**Lane 8: 25 mM with 0.03125mg/ml ethyl acetate *P. orientalis* (EAPO) + compound C**

**Lane 9: 25 mM with 0.0625mg/ml ethyl acetate *P. orientalis* (EAPO) + compound C**

**Lane 10: 25 mM with 0.125mg/ml ethyl acetate *P. orientalis* (EAPO) + compound C**

**Lane 11: 50 mM with 0.03125mg/ml ethyl acetate *P. orientalis* (EAPO)+ compound C**

**Lane 12: 50 mM with 0.0625mg/ml ethyl acetate *P. orientalis* (EAPO) + compound C**

**Lane 13: 50 mM with 0.125mg/ml ethyl acetate *P. orientalis* (EAPO) + compound C**


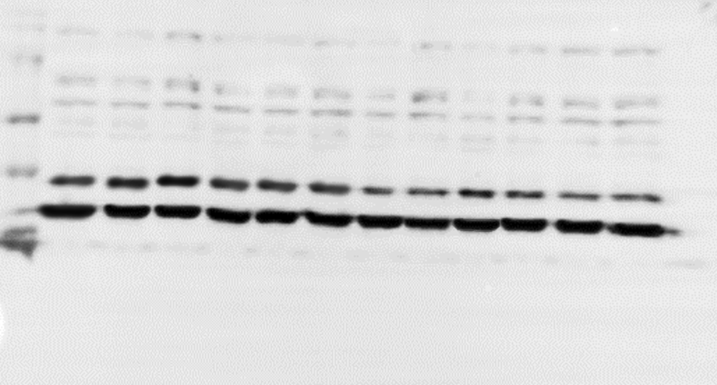


Phospho-MEK1/2– 43kDa

GAPDH – 36kDa

**Supplement 27.** Gel blot images for phospho-MEK1/2 (43 kDa) and GAPDH (36 kDa) expression in the whole cell lysate of retinal Müller cells treated with AICAR or compound C. (Corresponds to Figure 13C and 13D).
